# Supplementary material for: Genome-wide analysis of growth-regulating factors (GRFs) in Triticum aestivum
Source: PeerJ. 2021 Jan 19;9:e10701. doi: 10.7717/peerj.10701 (PMC7821759; doi:10.7717/peerj.10701)
Supplement: File S1 [file peerj-09-10701-s009.docx]

Supplemental File 1

**The gene sequences used in this research.**

Including CDS and protein sequences of TaGRFs, AeGRFs, TdGRFs, TuGRFs and protein sequences of other plant GRF genes.

1. **Protein sequences**

>TaGRF1-2A

MAAEGEDKKDANSVGGGGGGGENTLEAAEEAILQAVGQEPGQELEGEVEESADREGNGDDAGKEDSGCKD

LVLVEDPVLVEDPEEGFRYKSDVGFFKHGKSDLFIVMLAFLNTDDAQNIRFWVYHCFAVIVVFMYKAELS

VATAALQEEMRALFASVPEGAGASFTAMQLQELEQQSRVYQYMAARVPVPTHLVFPIWKSVTGASSEGAQ

NYPTLMGLATLCLDFGKSPEPEPGRCRRTDGKKWRCWRKTIPNEKYCERHMHRGRKRPVQVIVEDDEPDS

ASGSKSSSGKVTEGGKKTDDKSSSSKKLAVAAPAAVEFT

>TaGRF1-2B

MAAEGEDKKDANSVGGGGGENTLESAEEAILQAVGQEPGQELEGEAEESADREGNGDDAGKEDSGCKDLV

LVEDPVLVEDPEEAFQEEMRALLASVPEGAGASFTAMQLQELEQQSRVYQYMAARVPVPTHLVFPIWKSV

TGASSEGAQNYPTLMGLATLCLDFGKSPEPEPGRCRRTDGKKWRCWRKTIPNEKYCERHMHRGRKRPVQV

IVEDDEPDSASGSKSSSGKVTEGGKKTDDKSSSSKKLAVAAPAAVEFT

>TaGRF1-2D

MAAEGEDKKDANSVGGGGGGENTLEAAEEAILQAVGQEPGQELEGEAEESADREGNGDDTGKEDSGCKDL

VLVEDPVLVEDPEEAAATAALQEEMRALLASVPEGAGASFTAMQLQELEQQSRVYQYMAARVPVPTHLVF

PIWKSVTGASSEGAQNYPTLMGLATLCLDFGKSPEPEPGRCRRTDGKKWRCWRKTIPNEKYCERHMHRGR

KRPVQVIVEDDEPDSASGSKSSSGKVTEGGKKTENKSSSSKKLAVAAPAAVEFT

>TaGRF2-2A

MTVGKGAGTVTMAAPSPLVLGLGLGVGGSSSDSGRGDAEASAATRPSALTFMQRQELEHQVLIYRYFAAN

APVPVHLVLPIWKSVAASSSAPQRFPSLAGLGSMCYDHRSSMEPEPDRCRRTDGKKWRCSRGVVPGHKYC

ERHVHRGRSRARKPVEAAAATSAVPIRAMHAADAQGATSAHAAPPQRLGFSSPAGVYLAHGTARAT

>TaGRF2-2B

MTERRQEHSPPSKLPRLSGPDADDNDGAGTVTMAAPSPLVLGLGLGVGGSSSDSGRGDAEASAATRPSAL

TFMQRQELEHQVLIYRYFAANAPVPVHLVLPIWKSVAASSSAPQRFPSLAGLGSMCYDHRSSMEPEPDRC

RRTDGKKWRCSRGVMPGHKYCERHVHRGRGRARKPVEAAPATSAIPIRAMHAADAQGATSAHAAPPQRLG

FSSPAGVYLAHGTARAT

>TaGRF2-2D

MTERRQEHSPPSKLPRLSGPDADADDNDGAGTVTMAAPSPLVLGLGLGVGGSCSDSGRGDAEASAATRPS

ALTFMQRQELEHQVLIYRYFAANAPVPVHLVLPIWKSVAASSSAPQRFPSLAGLGSMCYDHRSSMEPEPD

RCRRTDGKKWRCSRGVVPGHKYCERHVHRGRGRARKPVEAAAATSAIPIRAMHAADAQGATSAHAAPPQR

LGFSSPAGVYLAHGTARAT

>TaGRF3-2A

MAMPFASLSPAADHHRSSPIFPFCRSSPLYSAGEEAAQQQQQQQHAMSGARWAAARPATFTAAQYEELEQ

QALIYKYLVAGVPVPPDLLLPIRRGFDSLASRFYHHHALGYGSYFGKKLDPEPGRCRRTDGKKWRCSKEA

AQDSKYCERHMHRGRNRSRKPVETQLVSHSQQLQQQAPAAAFHGHSPYPAIATGAGAPGSFALGSTAQLH

MDNAAAPYATAGAAGNKDFRYSAYGFRTSAMEDHNQFISAAMDTAMDNYSWRLLPAQNSSFSLSSYPMLS

TLSDLDQSAICSLAKTEREPLSFFGVGGGFDDDESAVKQENQTLRPFFDEWPKDRDSWPELQDHDSNHNN

EAFSATKLSISIPVTSSDFSTTAGSRSPHGIYSR

>TaGRF3-2B

MAMPFASLSPAADHHRSSPIFPFCRSSPLYSVGEETAHQQQQQHTMSGARWAARPATFTAAQYEELEQQA

LIYKYLVAGVPVPPDLLLPIRRGFDSLASRFYHHHALGYGSYFGKKLDPEPGRCRRTDGKKWRCSKEAAQ

DSKYCERHMHRGRNRSRKPVETQLVATPHHSHSQQLQQHAPAASAAAFHSHSPYPAIASGGGGSFAVGSA

QLHMDNAASPYATAGAAGNKDFRYSAYGFRTSAMEEHNQFISAAMETAMENYSCRLMPAQNSSFSLASYP

MLGTLGDLDQSAICSLAKTEREPLSFFGGGGGFDDDDSAVKQENQTLRPFFDEWPKDRDSWPELQDHDAN

NSSNAFSATKLSISIPVTSSDFSTTAGSRSPNGIYSR

>TaGRF3-2D

MAMPFASLSPAADHHRSSPIFPFCRSSPLYSAGEEAAQQQQQHAMSGARWAAMRPATFTAAQYQELEQQA

LIYKYLVAGVPVPPDLLLPIRRDFDSLASRFYHHHALGYGSYFGKKLDPEPGRCRRTDGKKWRCSKEAAQ

DSKYCERHMHRGRNRSRKPVETQLVATPHSHSHSQQLQQHAPAATAAAFHSHSPYPAIATGGGGGAAGSF

GLGSAQLHMDNAAAPYATAGAAGNKDFRYSAYGFRTSALEEHNQFISAAMDTAMDNYSWRLMPAQNSAFS

LSSYPMLGTLGDLDQSAICSLAKTEREPLSFGGGGGFEDDESAVKQENQTLRPFFDEWPKDRDSWPELQD

HDSNHNSNAFSATKLSISIPVTSSDFSTTAGSRSPHGIYSR

>TaGRF4-4A

MDLGGVLMAAADAGVGGGELGMLGSRLLKHGRGNAAAAEADERHEHGWGGGRPAAKQARVAGDSDAVSEA

VKAAAPYLLGTCSPGHGREKMLSFSSSQQPPSCPSAAAAAQAALPLYYGTPASCLGLSSVSLSASIQGAM

ARVRGPFTPSQWMELEHQALIYKYLAANIAVPHSLLVPIRRSVTSLYPSAYFGSSTLGWGPFQLGYSGSA

DLEPGRCRRTDGKKWRCSRDAVADQKYCERHMNRGRHRSRKHVEGQPGHAAKAMPATVAAAAAQPGALAT

GGGGGATAGAAICHEQQPLKSYSASTIDPCSLQYNREMASKQQHECEQVQDSDTLSMLTSMSARNTNTGS

MFPFSKEHHNHNPFEVTSSRPDYGLVSSDSLMSSPHSSLENVNLLTSQRALSSEQQSSLSLQHFADWPRT

PSQQGQGGGGLSWPDAENMQLAHQRTQQLSVSAAPMASSDLSSASTSPIHEKLMLSPLKLSREYSPIGLS

VAATAAAAAKDEGEANWMPMFRDSSMGGPLGEALNKNNGGNMEAKNYLSASLNLMTDAWDSSPLESSPVG

VLQRTAFGSVSSSTGSSPRQEYHGVYDGNPRDDLGSIVVNHPSIRLM

>TaGRF4-4B

MDLGGVLMAAADAGVGGGDLGMLGSRLLKHGRGNAAAAEADEHGWGSGRPPAKQARVAASAASGDSDAVS

EAVKAAAPYLLGTCSPGHGREKMLSFSSSQPPSCPSSAAAAAAQAALPLYYGTPASCSGLSSVSLSASIQ

NAMARVRGPFTPSQWMELEHQALIYKYLAANIAVPHNLLVPIRRSVTSLYPSAYFGSSTLGWGPFQLGYS

GSADLEPGRCRRTDGKKWRCSRDAVADQKYCERHMNRGRHRSRKHVEGQPGHAAKAMPATVAAAAAQPGA

LATGGGGGATAGAAAICHEQQPLKNYAANTIDPCSLQYNREMVSKQQQHECEQVQDSDTLSMLTSMSARN

TNTGSMFPFSKEHHNHNPFEVTSSRPDYGLVSSDSLMSSPHSSLENVNLLTSHSQRALSNEQQSSLSLQH

FADWPRTPSQQGQGGGGLSWPDAEDMQAHQRTQLSVSAAPMASPDLSSASTSPIHEKLMLSPLKLSREYS

PIGLSIAATAAAAKDEGEANWMPMFRDSSMGGPLGEALNKNNGGNMEAKNYLSASLNLMTDAWDSSPLES

SPVGVLQRTAFGSVSSSTGSSPRQEYHGVYDGNPRDDLGSIVVNHPSIRLM

>TaGRF4-4D

MDLGGVLMAAADAGVGGGDLGMLGSRLLKHGRGNEADEHGWGGGRPASKQARVAGDSDAVSEAVKAAAPY

LLGTCSPGHGREKMLSFSSSQPASCPSAAQAALPLYYGTPASCSGLSSVRGPFTPSQWMELEHQALIYKY

LAANIAVPHNLVVPIRRSVTSLYPSAYFGSSTLGWGPFQLGYSGSADLEPGRCRRTDGKKWRCSRDAVAD

QKYCERHMNRGRHRSRKHVEGQPGHAAKAMPATAAAAAQPGALATGGGGGAPAGAAICHEQQPLKNYAAS

TIDPCSLQYNREMVSKQQHECEQVQDSDTLSMLTSMSARNTNAGSMFPFSKEHHNHNPFEVTSSRPDYGL

VSSDSLMSSPHSSLENVNLLTSQRALSSEQQSSLSLQHFADWPRTPSQQGQGGGLSWPDAEDMQAHQRTQ

LSVSAVPMASSDLSSASTSPIHEKLMLSPLKLSREYSPIGLSVAATAAVAKDEGEANWMPMFRDSSMGGP

LGEALNKNNGGNMEAKSYLSASLNLMTDAWDSSPLESSPVGVLQRTAFGSVSSSTGSSPRQEYHGVYDGN

PRDDLGSIVVNHPSIRLM

>TaGRF5-4A

MELGQVLGYTPPATKDARSGGGFTQAAACPYPYPSPFLDEQKMLSFSKAAAAHQPPSGMDFGRSNEQRLL

LARSKMPFTPSQWMELEHQALIYKYLNAKAPIPSGLLISISKSFRPSSDRMPWRPVYQGFTNADSDPEPG

RCRRTDGKKWRCSKEAMAEHKYCERHINRNRHRSRKPVENQTRKNAKETPAAGSISAAVSQGGCKKAKAG

DELKPGSVSYWTDNLNRAMVSKARGNNPEEGNSAPLLNSTNQQHTLSLFSQLKQQSKPDKFSPAVDSESI

SSNTVLKPWERSNQQSSKDVSSTTLHDRGCLQSVLQDFSMHKNDKIESQKNNASVPSTFYSSTEGRHISC

LASNMMQVQEDCISSSWEIPQGGPLGEILTNSKNTDDLTNKCESRSYGWLLSLDEHEM

>TaGRF5-4D

MLPELTAAAMELGQVXXYTPPATKDARSGGGFAQAAACPYPYPSPFLDEQKMLSFSKAAAPPSSGMDFGR

SNEQRLLLARSKMPFTPSQWMELEHQALIYKYLNAKAPIPSSLLISISKSFRPSSDRMPWRPVYQGFTNA

DSDPEPGRCRRTDGKKWRCSKEAMAEHKYCERHINRNRHRSRKPVENQTRKNAKETPAAGSLSAAVSQGG

CKKAKAGDELKPGSVSYWTDNLNRAMVSKARGNNPEEGNSAPLLNSTNQQHTLSLFSQLKQQSKPDKFSP

AVDSESISSNTVLKPWERSNQQSSKDVSSTTLHDRGCLQSVLQDFSMHKNDKIEAQKNNASVPSTFYSPT

EGQHISCLASNMMQVQEDCISSSWEIPQGGPLGEILTNSKNTDDLTNKCESRSYGWLLSLDEHEM

>TaGRF6-4A

MLSSSAAMGMGLGGYGQQQQQQMQMQMQRGAGPVFTPAQWAELEQQALIYKYLMAGVPVPPDLLLPIRPH

HPAAGAAGTTFSFASPAASPFYHHHHPSMSYYAYYGKKLDPEPWRCRRTDGKKWRCSKEAHPDSKYCERH

MHRGRNRSRKPVESKSASPAHQSQQPPLSAVTSAARDAEPLPSLPAGAKTHGLSLGGAGSSQMHVDASSY

GGKYSLGAKSDVGELSFFSGASGNNNRGFTIDSPTDSSWHSMGSSLPPYQLSKPRDSGLMQGGFSYSHYE

PSQELGQVTIASLSHSQEQDCRSLGGGGGGGGGGGGGLMGNVKQENQPLRPFFDEWPGRRDSWSEMDDER

SNGTSFSTTQLSISIPMPRCD

>TaGRF7-6A

MKKLELDDGMVAGNGDAGQRPGVATLMPPAATHVPVASTMGAAGGLFTATQWVELQRQSLIYNHMAASLP

IPSYLLFSNINPAAAAAAAAAPSQAAPPYYCCYNTPLLVHHYQAQQAAQMSMLLQCMQQAVARRRCGRTD

GKKWRCARDAEPDQKYCQRHLNRVGRARPPPSARKQQHQHAAAAVVAHHRDRDKSAMTAPAAIHTSHGNK

SSGNTMLREDDDDYSRGLLDFTGGVCLAEQRENRLSLNYDNIAELYCNRQVTATPTATASAAASATAMDD

DAIHHGAAATWVGIGGPLGEALGLAVEIQWPAGST

>TaGRF8-6A

MADEKEADSLQPPSKQPRLSSADSNAGAVTMAVSSPLGLGLGLGLGGDSRGEQQAFEARAAAKSALTFMQ

QQELEHQVLIYRYFAAGAPVPVHLVLPIWKSVAASSFGPHRFPSLIGLGSLCFDYRSSMEPEPGRCRRTD

GKKWRCSRDVVQGHKYCERHVHRGRGRSRKPVEGAPSARAHSDSTATAPPCAIGFSPAGILHATHSAAAR

AT

>TaGRF8-6B

MADEKEADSLQPPSKQPRLSSADSNAGAVTTAVSSPLGLGLGLGLGGDSRGEQQAFEARAAAAAAKSALT

FMQQQELEHQVLIYRYFAAGAPVPVHLVLPIWKSVAASSFGPHRFPSLIGLGSLCFDYRSSMEPEPGRCR

RTDGKKWRCSRDVVQGHKYCERHVHRGRGRSRKPVEGASAAPAHSGSPTTAPPRAIGFSPAGILHATHSA

T

>TaGRF8-6D

MADEKEADSLQPPSKQPRLSSADSNAGAVTMAVSSPLGLGLGLGLGGDSRGEQQAFQARAAAAAAKSALT

FMQQQELEHQVLIYRYFAAGAPVPVNLVLPIWKSIAASSFGPHRFPSLIGLGSLCFDYRSSMEPEPGRCR

RTDGKKWRCSRDVLQGHKYCERHVHRGRGRSRKPVEGAPAAPAHSGSSTTAPPRAIGFSPAGILHATHSA

AARAT

>TaGRF9-6A

MAMPYASLSPAGDRRSSPAATATASLLPFCRSSPFSAGGNGGMGEEAPMDGRWMARPVPFTAAQYEELEH

QALIYKYLVAGVPVPPDLVLPIRRGIESLAARFYHNPLAIGYGSYLGKKVDPEPGRCRRTDGKKWRCAKE

AASDSKYCERHMHRGRNRSRKPVETQLVPHSQPPAASAVPPLATGFHGHSLYPAVGGGTNGGGGGGNNGM

SMPGTFSSALGPPQQHMGNNAASPYAALGGAGTCKDFRYTAYGIRSLADEQSQLMTEAMNTSVENPWRLP

PSSQTTTFPLSSYSPQLGATSDLGQNNSSNNNSGVKAERQQQQQPLSFPGCGDFGGGDSAKQENQTLRPF

FDEWPKTRDSWSDLTDDNSNVASFSATQLSISIPMTSPDFSAASSQSPNGMLFAGEMY

>TaGRF9-6B

MAMPYASLSPAGDRRSSPAATASLLPFCRSSPFSAGNGGMGEEARMAGRWMARPAPFTAAQYEELEHQAL

IYKYLVAGVPVPPDLVLPIRRGIETLAARFYHNPLAIGYGSYLGKKVDPEPGRCRRTDGKKWRCAKEAAS

DSKYCERHMHRGRNRSRKPVETQLVSHSQPPAASVVPPLATGFHNHSLYPAIGGTNGGGGGGNNGMPNTF

SSALGPPQQHMGNNASSPYAALGGAGTCKDFRYTAYGIRSLADEHSQLMTEAMNTSVENPWRLPPSSQTT

TFPLSSYAPQLGATSDLGQNNNSSSSNSAVKSERQQQQQPLSFPGCGDFGGGGAMDSAKQENQTLRPFFD

EWPKTRDSWSDLTDDNSSLASFSATQLSISIPMTSSDFSAASSQSPNGMLFAGEMY

>TaGRF9-6D

MAMPYASLSPAGDRRSSPAATASLLPFCRSSPFSAGGGNGGMGEEARMDGRWMARPVPFTAAQYEELEHQ

ALIYKYLVAGVPVPPDLVLPIRRGIESLAARFYHNPLAIGYGSYLGKKVDPEPGRCRRTDGKKWRCAKEA

ASDSKYCERHMHRGRNRSRKPVETQLVPHTQPPAASAVPPLATGFHSHSLYPAIGGSTNGGGGGGNNGMS

MPSTFSSALGPPQQHMGSNAASPYAALGGAGTCKDFRYTAYGIRSLADEHSQLMTEAMNTSVENPWRLPP

SSQTTSFPLSSYAPQLGATSDLGQNNNHNNSSSNSAVKSERQQPLSFPGCGDFGGGGMDSAKQENQTLRP

FFDEWPKTRDSWSDLTDDNSSLASFSATQLSISIPMTSSDFSAASSQSPNGMLFAGEMY

>TaGRF10-6A

MMMMGGRAGAGGVGAGGGRCPFTATQWQELEHQALIYKYMASGVPIPSDLLLPLRRSFLLDSALATSPSL

AFPPQAALGWGCFGMGFGRKAEDPEPGRCRRTDGKKWRCSKEAYPDSKYCEKHMHRGKNRSRKPVEMSLA

TPPPPPSSSASSSSSNVHSAVNVATTTTSPAPSYHRHAAATHDTTPYHALYGGPYSSAGRQQHASAYHHA

AQVSPFHLHLDTTHPHPPPSYYSTMDHSKDSYAYGHSVKEVHGGGEHAFFSSDVSTDRDHHHHQHQHHAS

AGGNGQWQFKQLGGMEPKQHNPTSLFPGYGNNAAYAIDLSSKEEDEEKERRQQQQHCFLLGADLRLDKPS

SGHGDSADQKPLRPFFDEWPHEKTGSKGSWMGLEGETQLSISIANELPITTTSRYHHGE

>TaGRF10-6B

MMMMGGRAGAGGVGAGGGRCPFTATQWQELEHQALIYKYMASGVPIPSDLLLPLRRSFLLDSALATSPSL

AFPPQAALGWGCFGMGFGRKAEDPEPGRCRRTDGKKWRCSKEAYPDSKYCEKHMHRGKNRSRKPVEMSLA

TPPPPPSSSASSSSSNVHSAVNAATTTTSPAPSYHRHAAATHDTTPYHTLYGGPYSSAGRQQHASAYHHA

AQVSPFHLHLDTTHPHPPPSYYSTMDHSKDSYAYGHSVKEVHGGGEHAFFSSDVTTDRDHHHHQHHAGAG

GNGQWQFKQLGGMEPKQHNPTSLFPGCGGYGNNAAYAIDLSSKEEDEEKERRQQQQHCFLLGADLRLDKP

SSGHGDSADQKPLRPFFDEWPHEKTGSKGSWMGLEGETQLSISIANELPITTTSRYHHGE

>TaGRF10-6D

MMMMGGRAGAGGVGAGGGRCPFTATQWQELEHQALIYKYMASGVPIPSDLLLPLRRSFLLDSALATSPSL

AFPPQAALGWGCFGMGFGRKAEDPEPGRCRRTDGKKWRCSKEAYPDSKYCEKHMHRGKNRSRKPVEMSLA

TPPPPPSSSASSSSSNVHSAVNVATTTSSPAPSYHRHAAATHDTTPYHALYGGPYSSAGRQQHASAYHHA

AQVSPFHLHLDTTHPHPPPSYYSSMDHSKDSYAYGHSVKEVHGGGEHAFFSSDVTTDRDHHHHHHQHQHH

ASAGGNGQWQFKQLGGMEPKQHNPTSLFPGCGGYGNNAAYAIDLSSKEEDEEKERRQQQQHCFLLGADLR

LDKPSSGHGDSADQKPLRPFFDEWPHEKTGSKGSWMGLEGETQLSISIANELPITTTSRYHHGE

>TaGRF11-7A

MLSSSAAMGMGLGGYGQQQQQQQMQMQMQMQRGAGPVFTPAQWAELEQQALIYKYLMAGVPVPPDLLLPI

RPHPAGAGATFSFANPAASPFYHHHHPSMSYYAYYGKKLDPEPWRCRRTDGKKWRCSKEAHPDSKYCERH

MHRGRNRSRKPVESKSASPAHQSQQPPLSAVTSATRDAEPLPSLPAGAKTHGLSLGGAGSSQMHVDASSY

GNKYSLGAKSDVGELSFFSGASGNNNRGFTIDSPTDSSWHSMGSSLPPYQLSKPRDSGLMQGGFSYSHFE

PSQELGQVTIASLSHSQEQDRRSFGGGGGGGGGGAGLMGNVKQENQPLRPFFDEWPGRRDSWSEMDDERS

NGTSFSTTQLSISIPMPRCD

>TaGRF11-7D

MLSSSAAMGMGLGGYGQQQQQQMQMQMQRGAEPVFTPAQWAELEQQALIYKYLMAGVPVPPDLLLPIRPH

PAGAAGTTFSFANPAASPFYHHHHPSMSYYAYYGKKLDPEPWRCRRTDGKKWRCSKEAHPDSKYCERHMH

RGRNRSRKPVESKSASPAHQSQQPQLSAVTSAARDAEPLPSLPAGAKTHGLSLGGAGSSQMHVDASSYGG

KYSLGAKSDVGELSFFSGASGNNNRGFTIDSPTDSSWHSMGSSLTPYQLSKPRDSGLMQGGFSYSHFEPS

QELGQVTIASLSHSQEQDRRSFGGGGGGGGGGAGLMGNVKQENQPLRPFFDEWPGRRDSWSEMDDERSNG

TSFSTTQLSISIPMPRCD

>TaGRF12-7A

MMLGGHGGGGGRCLFTASQWRELEHQALIYKYMAAGSQVPHELVLPLRHRDAAAFAGIDTAPSVACYPPP

QPSLGWGLYGAGAQYARKPEDPEPGRCRRTDGKKWRCSREAYGESKYCDRHMHRGKNRSRKPVEPMSSSS

VSSPAASYRQTTLSMSPPTPADTPSYGHGHLRAAASQSQINPLQLHLDTPSPPPSYHRYAPAQQYGGSFF

PSRQQVQEEAEAEARRRQHFLALGADLSLDKPDATTAASSTTEEKPLRRFFDEWPRDGNAVEVRPWNMGH

RDETLLSMSIPTTTASHPDLAAYRHHNDE

>TaGRF12-7B

MMLGGHGGGGGGGRCLFTASQWRELEHQALIYKYMAAGSQVPHELVLPLRHRDAAFAAIDTAPSLACYPP

PQPSLGWGLYGAGSQYARKPEDPEPGRCRRTDGKKWRCSREAYGESKYCDRHMHRGKNRSRKPVEPMSSA

SSVSSPAASYRHTALSMSPPTPADTPSYGHGHGHDHLRAAAGQSQINPLQLHLDTPSPPPSYHRYAPAQQ

YGGSFFPSRQQVQEEEARRRQHFLALGADLSLDKPDATTAASSTTEEKPLRRFFDEWPRDGNAVEGRPWN

MGHRDETLLSMSIPTTTASHPDLAAASRYHHHHNDE

>TaGRF12-7D

MMLGGHGGGGGGRCLFTASQWRELEHQALIYKYMAAGSQVPHELVLPLRHRDAAAFAAIDTVPSLACYPP

PQPSLGWGLYGAGAQYARKPEDPEPGRCRRTDGKKWRCSREAYGESKYCDRHMHRGKNRSRKPVEPMSSS

SVSSPAASYRQTALSMSPPTPADTPTYGHGHGHDHLRAAAGQSQSQINPLQLHLDTPSPPPSYHRYAPAQ

QYGGSFFQNRQQVQEEAEAEARRRQHFLALGADLSLDKPDATTAASSTTEEKPLRRFFDEWPRDGNAVEG

RPWNMGHRDETLLSMSIPTTTATHPDLAAASRYHHHHNDE

>AeGRF1

ILPQSESSVSPASPSDPIPAFCPLLLRGPFATMAAEGEDKKDANSVGGGGGGENTLEAAE

EAILQAVGQEPGQELEGEAEESADREGNGDDTGKEDSGCKDLVLVEDPVLVEDPEEATAA

LQEEMRALLASVPEGAGASFTAMQLQELEQQSRVYQYMAARVPVPTHLVFPIWKSVTGAS

SEGAQNYPTLMGLATLCLDFGKSPEPEPGRCRRTDGKKWRCWRKTIPNEKYCERHMHRGR

KRPVQVIVEDDEPDSASGSKSSSGKVTEGGKKTDNKSSSSKKLAVAAPAAVEFT

>AeGRF2

MAAPSPLVLGLGLGVGGSCSDSGRGDAEASAATRPSALTFMQRQELEHQVLIYRYFAANA

PVPVHLVLPIWKSVAASSSAPQRFPSLAGLGSMCYDHRSSMEPEPDRCRRTDGKKWRCSR

GVVPGHKYCERHVHRGRGRARKPVEAAAATSAIPIRAMHAADAQGATSAHAAPPQRLGFS

SPAGVYLAHGTARAT

>AeGRF3

MSGARWAAMRPATFTAAQYQELEQQALIYKYLVAGVPVPPDLLLPIRRGFDSLASRFYHH

HALGYGSYFGKKLDPEPGRCRRTDGKKWRCSKEAAQDSKYCERHMHRGRNRSRKPVETQL

VATPHSHSHSQQLQQHAPAATAAAFHSHSPYPAIASGGGGGAAGSFGLGSAQLHMDNAAA

PYATAGAAGNKDFRYSAYGFRTSALEEHNQFISAAMDTAMDNYSWRLMPSQPSSFSLASY

PMLGTLGDLDQSAICSLAKTEREPLSFGGGGGFEDDESAVKQENQTLRPFFDEWPKDRDS

WPELQDHDSNHNSNAFSATKLSISIPVTSSDFSTTAGSRSPHGIYSR

>AeGRF4

MELGQVLGYTPPATKDARSGGGFAQAAACPYPYPSPFLDEQKMLSFSKAAAPPSSGMDFG

RSNEQRLLLARSKMPFTPSQWMELEHQALIYKYLNAKAPIPSSLLISISKSFRPSSDRMP

WRPVYQGFTNADSDPEPGRCRRTDGKKWRCSKEAMAEHKYCERHINRNRHRSRKPVENQT

RKNAKETPAAGSLSAAVSQGGCKKAKAGDELKPGSVSYWTDNLNRAMVSKARGNNPEEGN

SAPLLNSTNQQHTLSLFSQLKQQSKPDKFSPAVDSESISSNTVLKPWERSNQQSSKDVSS

TTLHDRGCLQSVLQDFSMHKNDKIEAQKNNASVPSTFYSPTEGRHISCLASNMMQVQEDC

ISSSWEIPQGGPLGEILTNSKNTDDLTNKCESRSYGWLLSLDEHEM

>AeGRF5

MDLGGVLMAAADAGVGGGDLGMLGSRLLKHGRGNEADEHGWGGGRPASKQARVAGDSDAV

SEAVKAAAPYLLGTCSPGHGREKMLSFSSSQPASCPSAAQAALPLYYGTPASCSGLSSVR

GPFTPSQWMELEHQALIYKYLAANIAVPHNLVVPIRRSVTSLYPSAYFGSSTLGWGPFQL

GYSGSADLEPGRCRRTDGKKWRCSRDAVADQKYCERHMNRGRHRSRKHVEGQPGHAAKAM

PATAAAAAQPGALATGGGGGAPAGAAICHEQQPLKNYAASTIDPCSLQYNREMVSKQQHE

CEQVQDSDTLSMLTSMSARNTNAGSMFPFSKEHHNHNPFEVTSSRPDYGLVSSDSLMSSP

HSSLENVNLLTSQRALSSEQQSSLSLQHFADWPRTPSQQGQGGGLSWPDAEDMQAHQRTQ

LSVSAVPMASSDLSSASTSPIHEKLMLSPLKLSREYSPIGLSVAATAAVAKDEGEANWMP

MFRDSSMGGPLGEALNKNNGGNMEAKSYLSASLNLMTDAWDSSPLESSPVGVLQRTAFGS

VSSSTGSSPRQEYHGVYDGNPRDDLGSIVVNHPSIRLM

>AeGRF6

MADEKEADSLQPPSKQPRLSSADSNAGAVTMAVSSPLGLGLGLGLGGDSRGEQQAFQARA

AAAAAKSALTFMQQQELEHQVLIYRYFAAGAPVPVHLVLPIWKSIAASSFGPHRFPSLIG

LGSLCFDYRSSMEPEPGRCRRTDGKKWRCSRDVLQGHKYCERHVHRGRGRSRKPVEGAPA

APAHSGSSTTAPPRAIGFSPAGILHATHSAAARAT

>AeGRF7

MFVCSGGGNGGMGEEARMDGRWMARPVPFTAAQYEELEHQALIYKYLVAGVPVPPDLVLP

IRRGIESLAARFYHNPLAIGYGSYLGKKVDPEPGRCRRTDGKKWRCAKEAASDSKYCERH

MHRGRNRSRKPVETQLVPHTQPPAASAVPPLATGFHSHSLYPAIGGSTNGGGGGGNNGMS

MPSTFSSALGPPQQHMGSNAASPYAALGGAGTCKDFRYTAYGIRSLADEHSQLMTEAMNT

SVENPWRLPPSSQTTSFPLSSYAPQLGATSDLGQNNNHNNSSSNSAVKSERQQPLSFPGC

GDFGGGGMDSAKQENQTLRPFFDEWPKTRDSWSDLTDDNSSLASFSATQLSISIPMTSSD

FSAASSQSPNGMLFAGEMY

>AeGRF8

MMMMGGRAGAGGVGAGGGRCPFTATQWQELEHQALIYKYMASGVPIPSDLLLPLRRSFLL

DSALATSPSLAFPPQAALGWGCFGMGFGRKAEDPEPGRCRRTDGKKWRCSKEAYPDSKYC

EKHMHRGKNRSRKPVEMSLATPPPPPSSSASSSSSNVHSAVNVATTTSSPAPSYHRHAAA

THDTTPYHALYGGPYSSAGRQQHASAYHHAAQVSPFHLHLDTTHPHPPPSYYSSMDHSKD

SYAYGHSVKEVHGGGEHAFFSSDVTTDRDHHHHHHQHQHHASAGGNGQWQFKQLGGMEPK

QHNPTSLFPGCGGYGNNAAYAIDLSSKEEDEEKERRQQQQHCFLLGADLRLDKPSSGHGD

SADQKPLRPFFDEWPHEKTGSKGSWMGLEGETQLSISIANELPITTTSRYHHGE

>AeGRF9

MLSSSAAMGMGLGGYGQQQQMQMQMQRGAGPVFTPAQWAELEQQALIYKYLMAGVPVPPD

LLLPIRPHPAGAAGTTFSFANPAASPFYHHHHPSMSYYAYYGKKLDPEPWRCRRTDGKKW

RCSKEAHPDSKYCERHMHRGRNRSRKPVESKSASPAHQSQQPQLSAVTSAARDAEPLPSL

PAGAKTHGLSLGGAGSSQMHVDASSYGGKYSLGAKSDVGELSFFSGASGNNNRGFTIDSP

TDSSWHSMGSSLTPYQLSKPRDSGLMQGGFSYSHFEPSQELGQENQPLRPFFDEWPGRRD

SWSEMDDERSNGTSFSTTQLSISIPMPRCD

>AeGRF10

MMLGGHGGGGGGRCLFTASQWRELEHQALIYKYMAAGSQVPHELVLPLRHRDAAAFAAID

TVPSLACYPPPQPSLGWGLYGAGAQYARKPEDPEPGRCRRTDGKKWRCSREAYGESKYCD

RHMHRGKNRSRKPVEPMSSSSVSSPAASYRQTALSMSPPTPADTPTYGHGHGHDHLRAAA

GQSQSQINPLQLHLDTPSPPPSYHRYAPAQQYGGSFFQNRQQVQEEAEAEARRRQHFLAL

GADLSLDKPDATTAASSTTEEKPLRRFFDEWPRDGNAVEGRPWNMGHRDETLLSMSIPTT

TATHPDLAAASRYHHHHNGT

>TdGRF1

LKAKAPYRRTPPRSNPKDTFCPLLLRGPFATMAAEGEDKKDANSVGGGGGGGENTLEAAE

EAILQAVGQEPGQELEGEVEESADREGNGDDAGKEDSGCKDLVLVEDPVLVEDPEEAVAT

AALQEEMRALFASVPEGAGASFTAMQLQELEQQSRVYQYMAARVPVPTHLVFPIWKSVTG

ASSEGAQNYPTLMGLATLCLDFGKSPEPEPGRCRRTDGKKWRCWRKTIPNEKYCERHMHR

GRKRPVQVIVEDDEPDSASGSKSSSGKVTEGGKKTDDKSSSSKKLAVAAPAAVEFT

>TdGRF2

MTVGKGAGTVTMAAPSPLVLGLGLGVGGSSSDSGRGDAEASAATRPSALTFMQRQELEHQ

VLIYRYFAANAPVPVHLVLPIWKSVAASSSAPQRFPSLAGLGSMCYDHRSSMEPEPDRCR

RTDGKKWRCSRGVVPGHKYCERHVHRGRGRARKPVEAAAATSAVPIRAMHAADAQGATSA

HAAPPQRLGFSSPAGVYLAHGTARAT

>TdGRF3

RERAAYMAMPFASLSPAADHHRSSPIFPFCRSSPLYSQQQQQQQHAMSGARWAAARPATF

TAAQYEELEQQALIYKYLVAGVPVPPDLLLPIRRGFDSLASRFYHHHALGYGSYFGKKLD

PEPGRCRRTDGKKWRCSKEAAQDSKYCERHMHRGRNRSRKPVETQLVSHSQQLQQQAPAA

AFHGHSPYPAIATGAGAPGSFALGSTAQLHMDNAAAPYATAGAAGNKDFRSSCFKNVQQQ

LLV

>TdGRF4

TTLGPSPPSPSKRELRIAGLPLRSNPKDTFCPLLLRGPFATMAAEGEDKKDANSVGGGGG

ENTLESAEEAILQAVGQEPGQELEGEAEESADREGNGDDAGKEDSGCKDLVLVEDPVLLE

DPEEAVATAALQEEMRALLASVPEGAGASFTAMQLQELEQQSRVYQYMAARVPVPTHLVF

PIWKSVTGASSEGAQNYPTLMGLATLCLDFGKSPEPEPGRCRRTDGKKWRCWRKTIPNEK

YCERHMHRGRKRPVQVIVEDDEPDSASGSKSSSGKVTEGGKKTDDKSSSSKKLAVAAPAA

VEFT

>TdGRF5

TPEMTERRQEHSPPSKLPRLSGPDADDNDGTVTMAAPSPLVLGLGLGVGGSSSDSGRGDA

EASAATRPSALTFMQRQELEHQVLIYRYFAANAPVPVHLVLPIWKSVAASSSAPQRFPSL

AGLGSMCYDHRSSMEPEPDRCRRTDGKKWRCSRGVMPGHKYCERHVHRGRGRARKPVEAA

PATSAGNLRGLCCTGAGNFYFRLDH

>TdGRF6

EEREHIWRCPLPPCRRQPTTTAPPPSSPSAAPPLSTREETAHQQQQQHTMSGARWAARPA

TFTAAQYEELEQQALIYKYLVAGVPVPPDLLLPIRRGFDSLASRFYHHHAFGYGSYFGKK

LDPEPGRCRRTDGKKWRCSKEAAQDSKYCERHMHRGRNRSRKPVETQLVATPHHSHSQQL

QQHAPAASAAAFHSHSPYPAIATGGGGSFAVGSAQLHMDNAASPYATAGAAGNKDFSNNC

HVPFVLSSSQET

>TdGRF7

MARVRGPFTPSQWMELEHQALIYKYLAANIAVPHSLLVPIRRSVTSLYPSAYFGSSTLGW

GPFQLGYSGSADLEPGRCRRTDGKKWRCSRDAVADQKYCERHMNRGRHRSRKHVEGQPGH

AAKAMPATVAAAAAQPGALATGGGGGATAGAAICHEQQPLKSYSASTIDPCSLQYNREMA

SKQQHECEQVQDSDTLSMLTSMSARNTNTGSMFPFSKEHHNHNPFEVRSSRPDYGLVSSD

SLMSSPHSSLENVNLLTSQRALSSEQQSSLSLQHFADWPRTPSQQGQGGGGLSWPDAENM

QLAHQRTQQLSVSAAPMASSDLSSASTSPIHEKLMLSPLKLSREYSPIGLSVAATAAAAA

KDEGEANWMPMFRDSSMGGPLGEALNKNNGGNMEAKNYLSASLNLMTDAWDSSPLESSPV

GVLQRTAFGSVSSSTGSSPRQEYHGVYDGNPRDDLGSIVVNHPSIRLM

>TdGRF8

MAMGIRSNLPIHWGPSCSAKAAPFHCFFLLLYLSTLLSSLGACNNKKQINKHIHGYLQDK

AVASLSIFAHAEWGFLFFFLHLGMDFGRSNEQRLLLARSKMPFTPSQWMELEHQALIYKY

LNAKAPIPSGLLISISKSFRPSSDRMPWRPVYQGFTNADSDPEPGRCRRTDGKKWRCSKE

AMAEHKYCERHINRNRHRSRKPVENQTRKNAKETPAAGSISAAVSQGGCKKAKAGDELKP

GSVSYWTDNLNRFVYIES

>TdGRF9

MLSSSAAMGMGLGGYGQQQQQQMQMQMQRGAGPVFTPAQWAELEQQALIYKYLMAGVPVP

PDLLLPIRPHHPAAGAAGTTFSFASPAASPFYHHHHPSMSYYAYYGKKLDPEPWRCRRTD

GKKWRCSKEAHPDSKYCERHMHRGRNRSRKPVESKSASPAHQSQQPPLSAVTSAARDAEP

LPSLPAGAKTHGLSLGGAGSSQMHVDASSYGGKYSLGAKSDVGELSFFSGASGNNNRGFT

IDSPTDSSWHSMGSSLPPYQLSKPRDSGLMQGGFSYSHYEPSQELGQVTIASLSHSQEQD

RRSFGGGGGGGGGGGGGLMGNVKQENQPLRPFFDEWPGRRDSWSEMDDERSNGTSFSTTQ

LSISIPMPRCD

>TdGRF10

YPSPFLDEQKMLSFSKAAAPPSSGMDFGRSNEQRLLLARSKMPFTPSQWMELEHQALIYK

YLNAKAPIPSGLLISISKSFRPSSDRMPWRPVYQGFTNADSDPEPGRCRRTDGKKWRCSK

EAMAEHKYCERHINRNRHRSRKPVENQTRKNAKETPDAGSLSAAVSHGGCNKKAKAGDEL

KPGSVSYWTDNLNRAMVSKARGSNPEDGNSAPLLNSTNQQHTLSLFSQLKQQSKPDKFSP

AVDSESISSNTVLKPWERSNQQSSKDVSSTTLHDRGCLQSVLQDFSMHKNESQKINASVP

STFYSSTEGRHISCLASNMMQVQEDCISSSWEIPQGGPLGEILTNSKNTDDLTNKCESRS

YGWLLSLDEHEM

>TdGRF11

SDFLPPLACLPLSRKYYTFICFGFAFLSCTACLVAHLLVVLLSWVPGLSSVSLSASIQNA

MARVRGPFTPSQWMELEHQALIYKYLAANIAVPHNLLVPIRRSVTSLYPSAYFGSSTLGW

GPFQLGYSGSADLEPGRCRRTDGKKWRCSRDAVADQKYCERHMNRGRHRSRKHVEGQPGH

AAKAMPATVAAAAAQPGALATGGGGGATAGAAAICHEQQPLKNYAANTIDPCSLQYNREM

VSKQQQHECEQVQDSDTLSMLTSMSARNTNTGSMFPFSKEHHNHNPFEVTSSRPDYGLVS

SDSLMSSPHSSLENVNLLTSHSQRALSNEQQSSLSLQHFADWPRTPSQQGQGGGGLSWPD

AEDMQAHQRTQLSVSAAPMASPDLSSASTSPIHEKLMLSPLKLSREYSPIGLSIAATAAA

AKDEGEANWMPMFRDSSMGGPLGEALNKNNGGNMEAKNYLSASLNLMTDAWDSSPLESSP

VGVLQRTAFGSVSSSTGSSPRQEYHGVYDGNPRDDLGSIVVNHPSIRLT

>TdGRF12

MADEKEADSLQPPSKQPRLSSADSNAGAVTMAVLSPLGLGLGLGLGGDSRGEQQAFEARA

AAKSALTFMQQQELEHQVLIYRYFAAGAPVPVHLVLPIWKSVAASSFGPHRFPSLIGLGS

LCFDYRSSMEPEPGRCRRTDGKKWRCSRDVVQGHKYCERHVHRGRGRSRKPVEGAPSARA

HSDSTATAPPCAIGFSPAGILHATHSAAARAT

>TdGRF13

MGEEARMDGRWMARPVPFTAAQYEELEHQALIYKYLVAGVPVPPDLVLPIRRGIESLAAR

FYHNPLAIGYGSYLGKKVDPEPGRCRRTDGKKWRCAKEAASDSKYCERHMHRGRNRSRKP

VETQLVPHSQPPAASAVPPLATGFHSHSLYPAIGGGTNGGGGGGNNGMSMPGTFSSALGP

PQQHMGNNAASPYAALGGAGTCKDFRYTAYGIRSLADEQSQLMTEAMNTSVENPWRLPPS

SQTTTFPLSSYSPQLGATSDLGQNNSSNNNSGVKAERQQQQQPLSFPGCGDFGGGDSAKQ

ENQTLRPFFDEWPKTRDSWSDLTDDNSNVASFSATQLSISIPMTSSDFSAASSQSPNGM

>TdGRF14

MADEKEADSLQPPSKQPRLSSADSNAGAVTTAVSSPLGLGLGLGLGGDSRGEQQAFEARA

AAAAAKSALTFMQQQELEHQVLIYRYFAAGAPVPVHLVLPIWKSVSASSFGPHRFPSLIG

LGSLCFDYRSSMEPEPGRCRRTDGKKWRCSRDVVQGHKYCERHVHRGRGRSRKPVEGASA

APAHSGSPTTAPPRAIGFSPAGILHATHSAT

>TdGRF15

DSGRERKMAMPYASLSPAGDRRSSPAATASLLPFCRSSPFSASGNGGMGEEARMAGRWMA

RPAPFTAAQYEELEHQALIYKYLVAGVPVPPDLVLPIRRGIETLAARFYHNPLAIGYGSY

LGKKVDPEPGRCRRTDGKKWRCAKEAASDSKYCERHMHRGRNRSRKPVETQLVSHSQPPA

ASVVPPLATGFHNHSLYPAIGGTNGGGGGGNNGMPNTFSSALGPPQQHMGNNASSPYAAL

GGAGTCKDFRYTAYGIRSLADEHSQLMTEAMNTSVENPWRLPPSSQTTTFPLSSYAPQLG

ATSDLGQNNNSSSSNSAVKSERQQQQQPLSFPGCGDFGGGGAMDSAKQENQTLRPFFDEW

PKTRDSWSDLTDDNSSLASFSATQLSISIPMTSSDFSAASSQSPNVFVVSDLYQQ

>TdGRF16

MLSSSAAMGMGLGGYGQQQQQQQMQMQMQRGAGPVFTPAQWAELEQQALIYKYLMAGVPV

PPDLLLPIRPHPAGAGATFSFANPAASPFYHHHHPSMSYYAYYGKKLDPEPWRCRRTDGK

KWRCSKEAHPDSKYCERHMHRGRNRSRKPVESKSASPAHQSQQPPLSAVTSATRDAEPLP

SLPAGAKTHGLSLGGAGSSQMHVDASSYGNKYSLGAKSDVGELSFFSGASGNNNRGFTID

SPTDSSWHSMGSSLPPYQLSKPRDSGLMQGGFSYSHFEPSQELGQENQPLRPFFDEWPGR

RDSWSEMDDERSNGTSFSTTQLSISIPMPRCD

>TdGRF17

MMLGGHGGGGGRCLFTASQWRELEHQALIYKYMAAGSQVPHELVLPLRHRDAAAFAAIDT

APSLACYPPPQPSLGWGLYGAGAQYARKPEDPEPGRCRRTDGKKWRCSREAYGESKYCDR

HMHRGKNRSRKPVEPMSSSSSVSSPAASYRQTTLSMSPPTPADTPSYGHGHLRAAASQSQ

INPLQLHLDTPSPPPSYHRYAPAQQYGGSFPSRQQVQEEAEAEARRRQHFLALGADLSLD

KPDATTAASSTTEEKPLRRFFDEWPRDGNAVEVRPWNMGHRDETLLSMSIPTTTASHPDL

AAYRHHNAHSSD

>TdGRF18

MMLGGHGGGGGGGRCLFTASQWRELEHQALIYKYMAAGSQVPHELVLPLRHRDAAFAAID

TAPSLACYPPPQPSLGWGLYGAGSQYARKPEDPEPGRCRRTDGKKWRCSREAYGESKYCD

RHMHRGKNRSRKPVEPMSSASSVSSPAASYRHTALSMSPPTPADTPSYGHGHGHDHLRAA

AGQSQINPLQLHLDTPSPPPSYHRYAPAQQYGGSFFPSRQQVQEEEARRRQHFLALGADL

SLDKPDATTAASSTTEEKPLRRFFDEWPRDGNAVEGRPWNMGHRDETLLSMSIPTTTASH

PDLAAASRYHHHHNDE

>TuGRF1

MAAPSPLVLGLGLGVGGSSSDSGRDDAEASAATRPSALTFMQRQELEHQVLIYRYFAANA

PVPVHLVLPIWKSVAASSSAPQRFPSLAGLGSMCYDHRSSMEPEPDRCRRTDGKKWRCSR

GVVPGHKYCERHVHRGRGRARKPVEAAAATSAVPIRAMHAADAQGATSAHAAPPQRLGFS

SPAGVYLAHGTARAT

>TuGRF2

MAMPFASLSPAADHHRSSPIFPFCRSSPLYSAGEEAAQQQQQQQHAMSGARWAAARPATF

TAAQYEELEQQALIYKYLVAGVPVPPDLLLPIRRGFDSLASRFYHHHALGYGSYFGKKLD

PEPGRCRRTDGKKWRCSKEAAQDSKYCERHMHRGRNRSRKPVETQLVSHSQQLQQHGPAA

AFHGHSPYPAIATGAGAPGSFALGSTAQLHMDNAAAPYATAGAAGNKDFRYSAYGFRTSA

MEEHNQFISAAMDTAMDNYSWRLLPAQNSSFSLSSYPMLSTLSDLDQSAICSLAKTEREP

LSFFGVGGGFDDDESAVKQENQTLRPFFDEWPKDRDSWPELQDHDSNHNNEAFSATKLSI

SIPVTSSDFSTTAGSRSPHGIYSR

>TuGRF3

MAAEGEDKKDANSVGGGGGGGENTLEAAEEAILQAVGQEPGQELEGEVEESADREGNGDD

AGKEDSGCKDLVLVEDPVLVEDPEEAVATAALQEEMRALFASVPEGAGASFTAMQLQELE

QQSRVYQYMAARVPVPTHLVFPIWKSVTGASSEGAQNYPTLMGLATLCLDFGKSPEPEPG

RCRRTDGKKWRCWRKTIPNEKYCERHMHRGRKRPVQVIVEDDEPDSASGSKSSSGKVTEG

GKKTDDKSSSSKKLAVAAPAAVEFT

>TuGRF4

MADEKEADSLQPPSKQPRLSSADSNAGAVTMAVSSPLGLGLGLGLGGDSRSEQQAFEARA

AAKSALTFMQQQELEHQVLIYRYFAAGAPVPVHLVLPIWKSVAASSFGPHRFPSLIGLGS

LCFDYRSSMEPEPGRCRRTDGKKWRCSRDVVQGHKYCERHVHRGRGRSRKPVEGAPAAPA

HSDSTATAPPRAIGFSPAGILHATHSAAARAT

>TuGRF5

MMMMGGRAGAGGVGAGGGRCPFTATQWQELEHQALIYKYMASGVPIPSDLLLPLRRSFLL

DSALATSPSLAFPPQAALGWGCFGMGFGRKAEDPEPGRCRRTDGKKWRCSKEAYPDSKYC

EKHMHRGKNRSRKPVEMSLATPPPPPSSSASSSSSNVHSAVNVATTTTSPAPSYHRHAAA

THDTTPYHALYGGPYSSAGRQQHASAYHHAAQVSPFHLHLDTTHPHPPPSYYSTMDHSKD

SYAYGHSVKEVHGGGEHAFFSSDVSTDRDHHHHQHQHHASAGGNGQWQFKQLGGMEPKQH

NPTSLFPGYGNNAAYAIDLSSKEEDEEKERRQQQQHCFLLGADLRLDKPSSGHGDSADQK

PLRPFFDEWPHEKTGSKGSWMGLEGETQLSISIANELPITTTSRYHHGE

>TuGRF6

MMLGGHGGGGGRCLFTASQWRELEHQALIYKYMAAGSQVPHELVLPLRHRDAAAFAAIDT

APSLACYPPPQPSLGWGLYGAGAQYARKPEDPEPGRCRRTDGKKWRCSREAYGESKYCDR

HMHRGKNRSRKPVEPMSSSSSVSSPAASYRQTTLSMSPPTPADTPSYGHGHLRAAASQSQ

INPLQLHLDTPSPPPSYHRYAPAQQYGGSFFPSRQQVQEEAEAEARRRQHFLALGADLSL

DKPDATTAASSTTEEKPLRRFFDEWPRDGNAVEGRPWNMGHRDETLLSMSIPTTTASHPD

LAAYRHHNADE

>AtGRF1

MDLGVRVSGHETVSSPGQTELGSGFSNKQERSGFDGEDCWRSSKLSRTSTDGFSSSPASA

KTLSFHQGIPLLRSTTINDPRKGQEHMLSFSSASGKSDVSPYLQYCRNSGYGLGGMMNTS

NMHGNLLTGVKGPFSLTQWAELEQQALIYKYITANVPVPSSLLLSLKKSFFPYGSLPPNS

FGWGSFHLGFSGGNMDPEPGRCRRTDGKKWRCSRDAVPDQKYCERHINRGRHRSRKPVEG

QNGHNTNAAAAASAAAASTAAAVSKAAAGTSAVAMRGSDNNNSLAAAVGTQHHTNNQSTD

SLANRVQNSRGASVFPATMNLQSKETHPKQSNNPFEFGLISSDSLLNPSHKQASYATSSK

GFGSYLDFGNQAKHAGNHNNVDSWPEELKSDWTQLSMSIPMAPSSPVQDKLALSPLRLSR

EFDPAIHMGLGVNTEFLDPGKKTNNWIPISWGNNNSMGGPLGEVLNSTTNSPKFGSSPTG

VLQKSTFGSLSNSSSASSTIIGDNNNKNGDGKDPLGPTTLMNTSATAPSL

>AtGRF2

MDIGVHVLGSVTSNENESLGLKELIGTKQDRSGFIGEDCLQRSLKLARTTTRAEEEENLS

SSVAAAYCKTMSFHQGIPLMRSASPLSSDSRRQEQMLSFSDKPDALDFSKYVGLDNSSNN

KNSLSPFLHQIPPPSYFRSSGGYGSGGMMMNMSMQGNFTGVKGPFTLTQWAELEQQALIY

KYITANVPVPSSLLISIKKSFYPYGSLPPSSFGWGTFHLGFAGGNMDPEPGRCRRTDGKK

WRCSRDAVPDQKYCERHINRGRHRSRKPVEVQSGQNQTAAAASKAVTTPQQPVVAGNTNR

SNARASSNRSLAIGSQYINPSTESLPNNRGVSIYPSTVNLQPKESPVIHQKHRNNNNPFE

FGHISSDSLLNPNTAKTYGSSFLDFSSNQEKHSGNHNHNSWPEELTSDWTQLSMSIPIAS

SSPSSTHNNNNAQEKTTLSPLRLSRELDLSIQTDETTIEPTVKKVNTWIPISWGNSLGGP

LGEVLNSTTNSPTFGSSPTGVLQKSTFCSLSNNSSVSSPIAENNRHNGDYFHYTT

>AtGRF3

MDLQLKQWRSQQQQQHQTESEEQPSAAKIPKHVFDQIHSHTATSTALPLFTPEPTSSKLS

SLSPDSSSRFPKMGSFFSWAQWQELELQALIYRYMLAGAAVPQELLLPIKKSLLHLSPSY

FLHHPLQHLPHYQPAWYLGRAAMDPEPGRCRRTDGKKWRCSRDVFAGHKYCERHMHRGRN

RSRKPVETPTTVNATATSMASSVAAAATTTTATTTSTFAFGGGGGSEEVVGQGGSFFFSG

SSNSSSELLHLSQSCSEMKQESNNMNNKRPYESHIGFSNNRSDGGHILRPFFDDWPRSSL

QEADNSSSPMSSATCLSISMPGNSSSDVSLKLSTGNEEGARSNNNGRDQQNMSWWSGGGS

NHHHHNMGGPLAEALRSSSSSSPTSVLHQLGVSTQAFH

>AtGRF4

MDLQLKQWRSQQQNESEEQGSAATKISNFFFDQIQSQTATSAAAAPLPLFVPEPTSSSSF

SCFSPDSSNSSSSSRFLKMGNFFSWAQWQELELQALIYRYMLAGASVPQELLLPIKKSLL

HQSPMHFLHHPLQHSFPHHQPSWYWGRGAMDPEPGRCKRTDGKKWRCSRDVVAGHKYCDR

HIHRGRNRSRKPVETATTTITTTATTTASSFVLGEELGHGPNNNHFFSSGSSQPLHLSHQ

QSCSSEMKQESNNNKRPYEANSGFSNGRSDDGHILRHFFDDWPRSSDSTSSPMSSSTCHL

SISMPGNNTSSDVSLKLSTGNEEEEENMRNNNNEREQMNWWSNGGNHHNNMGGPLAEALR

SASSTSSVLHQMGISTQVFH

>AtGRF5

MMSLSGSSGRTIGRPPFTPTQWEELEHQALIYKYMVSGVPVPPELIFSIRRSLDTSLVSR

LLPHQSLGWGCYQMGFGRKPDPEPGRCRRTDGKKWRCSREAYPDSKYCEKHMHRGRNRAR

KSLDQNQTTTTPLTSPSLSFTNNNNPSPTLSSSSSSNSSSTTYSASSSSMDAYSNSNRFG

LGGSSSNTRGYFNSHSLDYPYPSTSPKQQQQTLHHASALSLHQNTNSTSQFNVLASATDH

KDFRYFQGIGERVGGVGERTFFPEASRSFQDSPYHHHQQPLATVMNDPYHHCSTDHNKID

HHHTYSSSSSSQHLHHDHDHRQQQCFVLGADMFNKPTRSVLANSSRQDQNQEEDEKDSSE

SSKKSLHHFFGEDWAQNKNSSDSWLDLSSHSRLDTGS

>AtGRF6

MATRIPFTESQWEELENQALVFKYLAANMPVPPHLLFLIKRPFLFSSSSSSSSSSSFFSP

TLSPHFGWNVYEMGMGRKIDAEPGRCRRTDGKKWRCSKEAYPDSKYCERHMHRGKNRSSS

RKPPPTQFTPNLFLDSSSRRRRSGYMDDFFSIEPSGSIKSCSGSAMEDNDDGSCRGINNE

EKQPDRHCFILGTDLRTRERPLMLEEKLKQRDHDNEEEQGSKRFYRFLDEWPSSKSSVST

SLFI

>AtGRF7

MDFLKVSDKTTIPYRSDSLFSLNQQQYKESSFGFRDMEIHPHPTPYAGNGLLGCYYYYPF

TNAQLKELERQAMIYKYMIASIPVPFDLLVSSPSSASPCNNKNIAGDLEPGRCRRTDGKK

WRCAKEVVSNHKYCEKHLHRGRPRSRKHVEPPYSRPNNNGGSVKNRDLKKLPQKLSSSSI

KDKTLEPMEVSSSISNYRDSRGSEKFTVLATTEQENKYLNFIDVWSDGVRSSEKQSTTST

PVSSSNGNLSLYSLDLSMGGNNLMGQDEMGLIQMGLGVIGSGSEDHHGYGPYGVTSSLEE

MSSWLAPMSTTPGGPLAEILRPSTNLAISGDIESYSLMETPTPSSSPSRVMKKMTSSVSD

ESSQV

>AtGRF8

MRMLLGIPYVDKSVLSNSVLERGKQDKSKLLLVDKCHYELDVEERKEDFVGGFGFGVVEN

SHKDVMVLPHHHYYPSYSSPSSSSLCYCSAGVSDPMFSVSSNQAYTSSHSGMFTPAGSGS

AAVTVADPFFSLSSSGEMRRSMNEDAGAAFSEAQWHELERQRNIYKYMMASVPVPPELLT

PFPKNHQSNTNPDVDTYRSGMFSIYADYKNLPLSMWMTVTVAVATGGSLQLGIASSASNN

TADLEPWRCKRTDGKKWRCSRNVIPDQKYCERHTHKSRPRSRKHVESSHQSSHHNDIRTA

KNDTSQLVRTYPQFYGQPISQIPVLSTLPSASSPYDHHRGLRWFTKEDDAIGTLNPETQE

AVQLKVGSSRELKRGFDYDLNFRQKEPIVDQSFGALQGLLSLNQTPQHNQETRQFVVEGK

QDEAMGSSLTLSMAGGGMEETEGTNQHQWVSHEGPSWLYSTTPGGPLAEALCLGVSNNPS

SSTTTSSCSRSSS

>AtGRF9

MQSPKMEQEEVEEERMRNKWPWMKAAQLMEFRMQALVYRYIEAGLRVPHHLVVPIWNSLA

LSSSSNYNYHSSSLLSNKGVTHIDTLETEPTRCRRTDGKKWRCSNTVLLFEKYCERHMHR

GRKRSRKLVESSSEVASSSTKYDNTYGLDRYNESQSHLHGTISGSSNAQVVTIASLPSAR

SCENVIRPSLVISEFTNKSVSHGRKNMEMSYDDFINEKEASMCVGVVPLQGDESKPSVQK

FFPEVSDKCLEAAKFSSNRKNDIIARSREWKNMNVNGGLFHGIHFSPDTVLQERGCFRLQ

GVETDNEPGRCRRTDGKKWRCSKDVLSGQKYCDKHMHRGMKKKHPVDTTNSHENAGFSPL

TVETAVRSVVPCKDGDDQKHSVSVMGITLPRVSDEKSTSSCSTDTTITDTALRGEDDDEE

YLSLFSPGV

>OsGRF1

MMMMSGRPSGGAGGGRYPFTASQWQELEHQALIYKYMASGTPIPSDLILPLRRSFLLDSA

LATSPSLAFPPQPSLGWGCFGMGFGRKAEDPEPGRCRRTDGKKWRCSKEAYPDSKYCEKH

MHRGKNRSRKPVEMSLATPPPPSSSATSAASNTSAGVAPTTTTTSSPAPSYSRPAPHDAA

PYQALYGGPYAAATARTPAAAAYHAQVSPFHLQLDTTHPHPPPSYYSMDHKEYAYGHATK

EVHGEHAFFSDGTEREHHHAAAGHGQWQFKQLGMEPKQSTTPLFPGAGYGHTAASPYAID

LSKEDDDEKERRQQQQQQQQQHCFLLGADLRLEKPAGHDHAAAAQKPLRHFFDEWPHEKN

SKGSWMGLEGETQLSMSIPMAANDLPITTTSRYHNDD*

>OsGRF2

MMAGGGSGRCLFTATQWQELEHQALIYKYMAAGAPVPPDLLLHLRHRAAAAAAADVDTVP

SLAFPPHHLGWGCYGAAAAQYGRRVEDPEPGRCRRTDGKKWRCSREAYGESKYCEKHMHR

GKNRSRKPVEMPPPAAAAVYRPSALSISPPPHDADAPSYGAGAGAPLQLHLDSFHASTSP

PPSYHRYAHTSSAPLFPSSAAGYGGGWSLSKEHCLTLGGAAADLSLDKPADHHHDATSAT

TEKPLRRFFDEWPRSDDGRTPWDGTQLSISIPTAAAASPDLAIAGAASRYHSNGDHLRTS

E*

>OsGRF3

MAMPFASLSPAADHRPSFIFPFCRSSPLSAVGEEAQQHMMGARWAAAVARPPPFTAAQYE

ELEQQALIYKYLVAGVPVPADLLLPIRRGLDSLASRFYHHPVLGYGSYFGKKLDPEPGRC

RRTDGKKWRCSKEAAPDSKYCERHMHRGRNRSRKPVEAQLVAPHSQPPATAPAAAVTSTA

FQNHSLYPAIANGGGANGGGGGGGGGGSAPGSFALGSNTQLHMDNAASYSTVAAGAGNKD

FRYSAYGVRPLADEHSPLITGAMDTSIDNSWCLLPSQTSTFSVSSYPMLGNLSELDQNTI

CSLPKVEREPLSFFGSDYVTVDSGKQENQTLRPFFDEWPKARDSWPDLADDNSLATFSAT

QLSISIPMATSDFSTTSSRSHNGIYSR*

>OsGRF4

MPPCLRRWPTTARPRQPRPPPSSPSAAPPRSPRKQREPAATTHFLGSSGACDNTVRRCVW

VGGCRGGGGVAMGEDAPMTARWPPAAAARLPPFTAAQYEELEQQALIYKYLVAGVPVPPD

LVLPIRRGLDSLAARFYNHPALGYGPYFGKKLDPEPGRCRRTDGKKWRCSKEAAPDSKYC

ERHMHRGRNRSRKPVETQLVAQSQPPSSVVGSAAAPLAAASNGSSFQNHSLYPAIAGSNG

GGGGRNMPSSFGSALGSQLHMDNAAPYAAVGGGTGKDLRYTAYGTRSLADEQSQLITEAI

NTSIENPWRLLPSQNSPFPLSSYSQLGALSDLGQNTPSSLSKVQRQPLSFFGNDYAAVDS

VKQENQTLRPFFDEWPKGRDSWSDLADENANLSSFSGTQLSISIPMASSDFSAASSRSTN

GD*

>OsGRF5

MLSSSPSAAAPGIGGYQPQRGAAVFTAAQWAELEQQALIYKYLVAGVPVPGDLLLPIRPH

SSAAATYSFANPAAAPFYHHHHHPSLSYYAYYGKKLDPEPWRCRRTDGKKWRCSKEAHPD

SKYCERHMHRGRNRSRKPVESKTAAPAPQSQPQLSNVTTATHDTDAPLPSLTVGAKTHGL

SLGGAGSSQFHVDAPSYGSKYSLGAKADVGELSFFSGASGNTRGFTIDSPTDSSWHSLPS

SVPPYPMSKPRDSGLLPGAYSYSHLEPSQELGQVTIASLSQEQERRSFGGGAGGMLGNVK

HENQPLRPFFDEWPGRRDSWSEMDEERSNQTSFSTTQLSISIPMPRCGSPIGPRLP*

>OsGRF6

MQGAMARVRGPFTPSQWIELEHQALIYKYLAANSPVPHSLLIPIRRSLTSPYSPAYFGSS

TLGWGSFQLGYSGSADPEPGRCRRTDGKKWRCSRDAVADQKYCERHMNRGRHRSRKHVEG

QPGHAAKAMPAAVAAAAASATQPSAPAAHSGGAVAGLAINHQHQQMKNYAANTANPCSLQ

YSRDLANKHNESEQVQDSDSLSMLTSISTRNTGSLFPFSKQHNPFEVSNSRPDFGLVSPD

SLMSSPHSSLENVNLLTSQSLNEQQSSVSLQHFVDWPRTPAQGALAWPDAEDMQAQRSQL

SISAPMASSDLSSASTSPIHEKLMLSPLKLSREYSPIGLGFAANRDEVNQGEANWMPMFR

DSLMGGPLGEVLTKNNNMEARNCLSESLNLLNDGWDSSSGFDSSPVGVLQKTTFGSVSSS

TGSSPRLENHSVYDGNSNLRDDLGSVVVNHPSIRLV*

>OsGRF7

MAMATPTTNGSFLLGSGGYPGAQILSFSSSGHSGNGLDCGSSDVARMQGVLARVRGPFTP

TQWMELEHQALIYKHIVANAPVPAGLLLPIRRSLHPPVFPHFSSGGILGSSSLGWGSFQL

GYSGSADSEPGRCRRTDGKKWRCSRDAVVDQKYCERHINRGRHRSRKHVEGQSSHAAKAT

VPAIAQPPIGASNGKLSGSHGVSNELTKTLATNRMMLDKANLIERSQDYTNQQHNILQNN

TKGDNWSEEMSSQADYAVIPAGSLMNTPQSANLNPIPQQQRCKQSLFGKGIQHDDIQLSI

SIPVDNSDLPTNYNKAQMDHVVGGSSNGGNNTRASWIPGSWEASIGGPLGEFFTNTSSAS

DDKGKSRHPPSLNLLADGHTTSPQLQSPTGVLQMTSFSSVPSSTVSSPAGSLCNGLLTSG

LVNAQTVQTL*

>OsGRF8

MLSSCGGHGHGNPRSLQEEHHGRCGEQQGGGGGGGQEQEQDGFLVREARASPPSPSSSSF

LGSTSSSCSGGGGGGQMLSFSSPNGTAGLGLSSGGSMQGVLARVRGPFTPTQWMELEHQA

LIYKHIAANVSVPSSLLLPIRRSLHPWGWGSFPPGCADVEPRRCRRTDGKKWRCSRDAVG

DQKYCERHINRGRHRSRKHVEGRKATLTIAEPSTVIAAGVSSRGHTVARQKQVKGSAATV

SDPFSRQSNRKFLEKQNVVDQLSPMDSFDFSSTQSSPNYDNVALSPLKLHHDHDESYIGH

GAGSSSEKGSMMYESRLTVSKETLDDGPLGEVFKRKNCQSASTEILTEKWTENPNLHCPS

GILQMATKFNSISSGNTVNSGGTAVENLITDNGYLTARMMNPHIVPTLL*

>OsGRF9

MFADFSAAAMELGEVLGLQGLTVPSTKEGDLSLIKRAAAGSFTQAAAASYPSPFLDEQKM

LRFAKAAHTLPSGLDFGRENEQRFLLSRTKRPFTPSQWMELEHQALIYKYLNAKAPIPSS

LLISISKSFRSSANRMSWRPLYQGFPNADSDPEPGRCRRTDGKKWRCSKEAMADHKYCER

HINRNRHRSRKPVENQSRKTVKETPCAGSLPSSVGQGSFKKAKVNEMKPRSISYWTDSLN

RTMANKEKGNKAAEENNGPLLNLTNQQPTLSLFSQLKQQNKPEKFNTAGDSESISSNTML

KPWESSNQQNNKSIPFTKMHDRGCLQSVLQNFSLPKDEKMEFQKSKDSNVMTVPSTFYSS

PEDPRVSCHAPNMAQMQEDSISSSWEMPQGGPLGEILTNSKNPDDSIMKPEARPYGWLLN

LEDHAM*

>OsGRF10

MDEEKEADSPQPPSKLPRLSGADPNAGVVTMAAPPPPVGLGLGLGLGGDSRGERDVEASA

AAAHKATALTFMQQQELEHQVLIYRYFAAGAPVPVHLVLPIWKSVASSSFGPHRFPSLAV

MGLGNLCFDYRSSMEPDPGRCRRTDGKKWRCSRDVVPGHKYCERHVHRGRGRSRKPVEAS

AAATPANNGGGGGIVFSPTSVLLAHGTARAT*

>OsGRF11

MAAEGEAKKDSASNPPGGGGGGGGGEEEEDSSLAVGEAAVGVGEAGGGGGGGEKADREEE

EGKEDVEEGGVCKDLVLVEDAVPVEDPEEAAATAALQEEMKALVESVPVGAGAAFTAMQL

QELEQQSRVYQYMAARVPVPTHLVFPIWKSVTGASSEGAQKYPTLMGLATLCLDFGKNPE

PEPGRCRRTDGKKWRCWRNAIANEKYCERHMHRGRKRPVQLVVEDDEPDSTSGSKPASGK

ATEGGKKTDDKSSSSKKLAVAAPAAVEST*

>OsGRF12

MLAEGRQVYLPPPPPSKLPRLSGTDPTDGVVTMAAPSPLVLGLGLGLGGSGSDSSGSDAE

ASAATVREARPPSALTFMQRQELEQQVLIYRYFAAGAPVPVHLVLPIWKSIAAASSFGPQ

SFPSLTGLGSLCFDYRSSMEPEPGRCRRTDGKKWRCSRDVVPGHKYCERHVHRGRGRSRK

PMEASAAVAPTYLPVRPALHTVATLATSAPSLSHLGFSSASKVLLAHTTTGTTRAT*

>ZmGRF1

MAMPYASLSPAGAADHRSSTATASLVPFCRSTPLSAGGGLGEEDAQASARWPAARPVVPFTPAQYQELEQQALIYKYLVA

GVPVPPDLVVPIRRGLDSLATRFYGQPTLGYGPYLGRKLDPEPGRCRRTDGKKWRCSKEAAPDSKYCERHMHRGRNRSRK

PVETQLAPQSQPPAAAAVSAAPPLAAAAAATTNGSGFQNHSLYPAIAGSTGGGGGVGGSGNISSPFSSSMGGSSQLHMDS

AASYSYAALGGGTAKDLRYNAYGIRSLADEHNQLIAEAIDSSIESQWRLPSSSFPLSSYPHLGALGDLGGQNSTVSSLPK

MEKQQPPSSFLGNDTGAGMAMGSASAKQEGQTLRHFFDEWPKARDSWPGLSDETASLASFPPATQLSMSIPMASSDFSVA

SSQSPNGESRTFLLATDRR

>ZmGRF2

MEGGRDVFLGAAARAPPPPPSCPFHGSATATRSGGAQMLSFSSNGVAGLGLCSGASKMQG

VLSRVRRPFTPTQWMELEHQALIYKHFAVNAPVPSSLLLPIKRSLNPWSSLGSSSLGWAP

FRSGSADAEPGRCRRTDGKKWRCSRDAVGDQKYCERHIKRGCHRSRKHVEGRKATPTTAD

PTMAVSGGSLLHSHAVAWQQQGKSSAANVTDPFSLGSNRNLLDKQNLGDQFSVSTSMDSF

DFSSSHSSPNQAKVAFSPVAMQHEHDQLYLVHGAGSSAENVNKSQDGQLLVSRETIDDGP

LGEVFKGKSCQSASADILTDHWTSTRDLRPPTGVLQMSSSNTVPAENHTSNSSYLMARMA

NSQTVPTLH

>ZmGRF3

MMMMSSGRAGGGATAGRYPFTASQWQELEHQALIYKCLASGKPIPSYLMPPLRRILDSAL

ATSPSLAYPPQPSLGWGCFGMGFTRKADEDPEPGRCRRTDGKKWRCSKEAYPDSKYCEKH

MHRGKNRSRKPVEMSLATPAPAPAPAAATTATATSSPAPSYHRPAHDATPSPYHALYGGG

GGGGGSPYSASARPGATGGGGAYHHAQHVSPFHLHLETTHPHPPPPYNYSADQRDYAYGH

AAAKEVGEHAFFSDGAGERVDRQAAAGQWQFRQLGVETKPGPTPLFPVAGYGHGAASPYG

VEMGKDDDEQEERRRQHCFVLGADLRLERPSSGHGHGHDHDDAAAAQKPLRPFFDEWPHQ

KGDKAGSWMGLDGETQLSMSIPMAATDLPVTSRFRNGGHYE

>ZmGRF4

MAAEGEAKNPSGGGEGGNPQHQQAVQAAPAEPPTAQGEAVQEAGAQATGQEPEGEKANRDGEGSAGEKDDGACRDLVLVE

DPEVLAVEDPEEAAATAALQEEMKALVASVPDGAGAAFTAMQLQELEQQSRVYQYMAARVPVPTHLVFPVWKSVTGASSE

GAQKYPTLLGLATLCLDFGKNPEPEPGRCRRTDGKKWRCWRNTIPNEKYCERHMHRGRKRPVQVVEEAEPDSASGSKSAP

SKATEGAKKVDDKSPGSKKLAVAAAAAAAVQST

>ZmGRF5

MGMAMPFASPSPAADHRPSSLLPFCRAAPLSAAGEDAAQQHAMSGRWAARPALFTAAQYE

ELEHQALIYKYLVAGVPVPPDLLLPLRRGFVFHQPPALGYGPYFGKKVDPEPGRCRRTDG

KKWRCSKEAAPDSKYCERHMHRGRNRSRKPVEAQLAPPPHAQPQQQQQAPAPAAGFQNHS

LYPSILTGNGGGGVGAGAGGGTFGLGPTSQLHMDSAAAYATAAGGGSKYLRYSAYGVKSL

SDEHSTLLSGGMDPSMMDNSWRLLPSQTNTFQATSYPVFGTLSGLDESTIASLPKTQREP

LSFFGSDFVTAAKQENQTLRPFFDEWPKSRDSWPELGEDSSLGFSATQLSISIPMATSDF

SNTSSRSPGGIPSR

>ZmGRF6

MAMPFASLSPAADHRPSSLLPYCRAAPLSAVGEDAAAQAQQQQQHAMSGRWAARPPALFTAAQYEELEHQALIYKYLVAG

VPVPPDLLLPLRRGFVYHQPALGYGPYFGKKVDPEPGRCRRTDGKKWRCSKEAAPDSKYCERHMHRGRNRSRKPVEAQLV

PPPHAQQQQQQQAPAPTAGFQSHPMYPSILAGNGGGGGGVGGGAGGGGTFGLGPTSQLHMDSAAAYATAAGGGSKDLRYS

AYGVKSLSDEHSQLLSGGGGMDASMDNSWRLLPSQTAATFQATSYPLFGALSGLDESTIASLPKTQREPLSFFGSDFVTP

KQENQTLRPFFDEWPKSRDSWPELNEDNSLGSSATQLSISIPMAPSDFNTSSRSPNGIPSR

>ZmGRF7

MMLSGHGGGRRLFTASQWQELEHQALIFKYMASGAPVPHDLVLPLRLATGVDTAPSLAFP

PQPSPSLAYWGCYGAGAPFGRKAAEDTEPGRCRRTDGKKWRCSREAHGDSKYCEKHIHRG

KSRSRKPVEVTSSPAAGPAAAYRPSAISTISPPRAADAPPPSLAYPQQHLLHGASSAAGA

AARVPAGALQLHLDASLHAAAAAASPSPPPSYHRYAHYTPPASSLFPGGGYGYDYDYGQS

KELRRRHFHALGADLSLDKPLPEPDTGSDEKQPLRRFFDEWPRESGDMAADDATQLSISI

PAASPSDLAATSASAAAARFHNGEAASQRLHFHWFLAYDRIADWCCRFIIV

>ZmGRF8

MSAEFCAAAGVVAMELGVGDALGLQQGIAITAPSPRDSDLGLLKRAGLTQAAAAAPYPSP

FLDGEKMLRFSKAAHTSHSGLDFGGPGEQAFLLSRTKMPFTPSQWMELGHQALIYKYLNA

KAPIPSSLLISISKSFRSSNRVSWRPLYQGYTNADSDPEPGRCRRTDGKKWRCSKEAMAD

HKYCERHINRNRHRSRKPVENQPKKTTKEVPAAAGSLPCAGPQGSLKKAKVNDSKPGTVS

YWADSLNRTMLSREKANKPTEDSSLLLTSTNSQPTWSLLSQLKQQNKPDKLGPTLENESN

PDTILKAWGGNQPSHKSISSTERHDAESLQSVLQNLSLAQNEKMESEKDKYSDSVLVSST

FYSAGGPRATCLTPNMTQVKQDCISSSWEMPQGGPLGEILTNSKNSKDLSKCKPRSYGWL

LNLDHAP

>ZmGRF9

MMMMSGRAATAGRYPFTASQWQELEHQALIYKCLASGKPIPSYLMPPLRRILDSALATSP

SLAAFQPQPSLGWGGCFGMGFSRKPADEDPEPGRCRRTDGKKWRCSKEAYPDSKYCEKHM

HRGKNRSRKPVEMSLATPAPPASSAATTSTSPAPSYHRPAPAAHDAVPYHAPYGAAYHHT

QTQVMSPFHLHLETTHPHPPPPPPYYYADQRDYAYGKEVGERAFFSDGAGERDRQQQAAG

QWQFKQLGTMEATKPCPTPTPLLPAAGYGVGQAKEDEEEETRRQQQQHCFVLGADLRLAE

RPSGAHDDAAQKPLRHFFDEWPHEKGSKAGWWIGGLDGETTQLSMSIPMAAAADLPVTSR

YRT

>ZmGRF10

MTAEGEAKNPSAGGGGDNPQHQQAAPAPAPAQGEVAQEAAVQGTGQEQERDKADREVQGG

AGEKDDGACRDLVLVEDPEVLAVEDPEEAAATAALQEEMKALVASIPDGAGAAFTAMQLQ

ELEQQSRVYQYMAARVPVPTHLVFPVWKSVTGASSEGAQKYPTLMGLATLCLDFGKNPEP

EPGRCRRTDGKKWRCWRNTIPNEKYCERHMHRGRKRPVQVFLEDDEPDSASGSKPAAPGK

ATEGAKKADDKSPSSKKLAVAAPAAVQST

>ZmGRF11

MMLSGHGGGRRLFTASQWQELEHQALIFKYMASGAPVPHDLVLPLRLATGVDTAPSLAFP

PQPSPSLAYWGCYGAGAPFGRKAEDPEPGRCRRTDGKKWRCSRGGPRRLQVLREAHPPRE

EPFKKACGSDLPRRLPPVRVLHLAASRGRRAAAAAGPRPPAAAASPPRRSLSSRPRPRRW

RSPAPPRLEPARGVAAAVLPQVRPLPRSLHAAAAAVALRLRAVQGASGGGGAQAAALPRA

RGRPEPRQAAGRRRGRGEAPAAFLRRVAAGERRHEAVVGGGGGRDAALHLHPRGFALL

>ZmGRF12

MLSSASSAGAAMGMGGGYQHQPLPLPQRGAAAAVFTAAQWAELEQQALIYKYLMAGVPVP

PDLLRPAPHAAAFSFASPAASPFYHHHHHHPSLSYYAYYGKKLDPEPWRCRRTDGKKWRC

SKEAHPDSKYCERHMHRGRNRSRKPVESKTASSPPQLSTVVTTTTTREAAAATPLESLAG

AGGKAHGLSLGGGAGSSHLSVDASNTHFRYGSKYPLGAKSDAGELSFFSGAPGNSRGFTI

DSPADNSWHSLPSNVPPFTLSKGRDSGLLPGAPPVVVQQQRGRRWWVAGEREAGEPAAEA

LLRRVAWDAGLVVGDGRREVQ

>ZmGRF13

MSAEFCAAAAGAVAMELGVGDVMGLQQGIAAATGPSSGDSDLGLLKRAGLAQAATSYPSPFLDQQKMLRFSKAAAAHTSP

SGLDFGGGPSEQAFLLSRTKRPFTPSQWMELEHQALIYKYLNAKAPIPSSLLVSISKSFRSSNRVSWRPLYQGYANADSD

PEPGRCRRTDGKKWRCSKEAMPDHKYCERHINRNRHRSRKPVENQPRKATKEVTTAAAGSLPCAGPQGSLKKAKVNDSKP

GTGSYWTDSLNRTMLSREKANKPTDDESLLLSSTKNSQPTLSLLTQLKQQNKPDKLGPTPENEPNSDTMLKAWGGSHHKS

ISSTQRHDAESLQSVLQNFSLAQNDRLESEKNRYSDSVLVSSAFYSADGPQTTCLTPNMTQVQQDCISSSWEMPQGGPLG

EILTNSKISEDLSKCGSRSYGWLLNLDHAP

>ZmGRF14

MLSSASSAAGAAMGMGGGGYAHQPPPQRTVFTAAQWAELEQQALIYKYLMAGVPVPPDLL

LPVRPGPAAAFSFAGPAAASPFYHQHHPSLSYYAYYGKKLDPEPWRCRRTDGKKWRCSKE

AHPDSKYCERHMHRGRNRSRKPVESKTASSSSPAHPSPPQLSTVTTTAPLEPLAAAGGKV

HGLSLGGGAAGSSHLGVDASNAHYRYGSNRYPLGAKPDGGELSFFSGASSGNNSRGGFTI

DSPSDNNSWHSALASSVPPFTLSTKSGDSGLLPGAYASYSQSHSHMEPPRELGQVTIASL

AQEQERQQPFSGGMLGNVKQENQNQPLRPFFDEWPGTRADSWPPEMDGAPRAGRTSFSSS

TTQLSISIPMPRCD

1. **CDS sequences**

>TaGRF1-2A

ATGGCGGCGGAAGGGGAGGACAAGAAGGATGCTAATTCCGTTGGAGGCGGCGGCGGCGGCGGCGAGAACA

CCCTGGAGGCGGCGGAGGAGGCGATTCTGCAGGCGGTAGGGCAAGAGCCCGGTCAAGAATTGGAGGGCGA

GGTGGAGGAGAGCGCAGATCGAGAGGGGAACGGCGACGACGCTGGGAAGGAAGATAGTGGGTGTAAAGAT

CTGGTCCTGGTAGAGGACCCTGTCCTGGTCGAGGATCCAGAGGAAGGATTCCGCTACAAATCTGACGTCG

GGTTCTTTAAGCATGGAAAATCTGATTTATTCATTGTTATGCTAGCTTTTCTTAATACTGACGATGCACA

AAACATTAGATTTTGGGTTTACCATTGCTTCGCTGTCATTGTTGTCTTTATGTACAAGGCCGAGCTATCG

GTAGCAACTGCAGCACTTCAGGAAGAAATGAGAGCGCTTTTCGCGTCTGTCCCTGAAGGTGCTGGGGCAT

CATTTACTGCGATGCAGCTGCAGGAGCTAGAGCAGCAGTCTCGTGTATACCAGTATATGGCTGCCCGTGT

GCCTGTGCCTACCCATCTCGTCTTCCCCATCTGGAAGAGTGTTACCGGTGCATCCTCTGAAGGCGCGCAG

AATTACCCTACATTGATGGGATTGGCAACACTCTGCTTGGACTTCGGGAAGAGCCCAGAACCAGAACCAG

GAAGGTGCCGGCGAACAGATGGAAAAAAGTGGCGGTGCTGGAGAAAAACAATCCCAAACGAGAAATATTG

TGAACGCCATATGCATCGTGGTCGCAAGCGTCCTGTACAGGTTATTGTTGAGGATGACGAGCCTGATTCC

GCATCAGGGTCAAAATCGTCATCTGGCAAAGTCACTGAAGGAGGCAAGAAGACTGACGACAAGAGTTCAA

GTAGCAAGAAGCTTGCAGTGGCAGCACCAGCTGCTGTGGAGTTTACATGA

>TaGRF2-2A

ATGACGGTAGGGAAGGGCGCAGGGACGGTGACCATGGCGGCGCCGTCGCCGCTGGTTCTTGGGCTGGGTC

TCGGCGTAGGCGGCAGCAGCAGTGACAGCGGACGCGGCGACGCGGAGGCCTCTGCGGCGACGCGGCCGTC

GGCGCTGACGTTCATGCAGCGGCAGGAGCTGGAGCACCAGGTGCTCATCTACCGCTACTTCGCCGCCAAC

GCTCCCGTGCCCGTGCACCTCGTGCTCCCCATCTGGAAGAGCGTCGCCGCTTCCTCCTCCGCCCCGCAGA

GGTTTCCATCCCTGGCGGGGCTGGGGAGCATGTGCTACGACCACAGGAGCAGCATGGAGCCGGAGCCGGA

CCGGTGCCGGCGCACGGACGGCAAGAAGTGGCGGTGCTCGCGCGGCGTGGTGCCGGGGCACAAGTACTGC

GAGCGCCACGTCCACCGCGGCCGCAGCCGTGCAAGAAAGCCTGTGGAAGCCGCGGCGGCCACATCAGCCG

TCCCGATCCGCGCGATGCACGCCGCCGACGCGCAGGGCGCCACCAGTGCGCACGCGGCGCCACCGCAGCG

CCTCGGCTTCTCCTCCCCCGCCGGCGTCTACCTGGCGCACGGCACCGCCCGTGCCACCTGA

>TaGRF3-2A

ATGGCGATGCCCTTTGCCTCCCTGTCGCCGGCAGCCGACCACCACCGCTCCTCCCCCATCTTCCCCTTCT

GCCGCTCCTCCCCTCTCTACTCGGCAGGGGAGGAGGCGGCGCAGCAGCAGCAGCAGCAGCAGCACGCGAT

GAGCGGCGCGAGGTGGGCGGCGGCGAGGCCGGCGACCTTCACGGCGGCGCAGTACGAGGAGCTGGAGCAG

CAGGCGCTCATCTACAAGTACCTCGTCGCCGGCGTGCCCGTCCCGCCGGATCTCCTCCTCCCCATCCGCC

GGGGCTTCGACTCCCTCGCCTCGCGCTTCTACCACCACCACGCCCTTGGGTACGGGTCCTACTTCGGGAA

GAAGCTGGATCCGGAGCCGGGGCGGTGCCGGCGGACGGACGGCAAGAAGTGGCGGTGCTCCAAGGAGGCC

GCCCAGGACTCCAAGTACTGCGAGCGCCACATGCACCGCGGCCGCAACCGTTCAAGAAAGCCTGTGGAAA

CGCAGCTCGTCTCCCACTCCCAGCAGCTGCAGCAGCAGGCCCCCGCCGCCGCGTTCCACGGCCACTCGCC

GTACCCGGCGATCGCCACTGGCGCCGGCGCGCCCGGCTCCTTCGCCCTGGGGTCTACTGCTCAGCTGCAC

ATGGATAATGCTGCTGCGCCTTACGCGACCGCTGGCGCCGCCGGGAACAAAGATTTCAGGTATTCTGCCT

ATGGGTTTAGGACTTCGGCGATGGAGGACCACAACCAGTTCATCAGTGCGGCCATGGACACCGCCATGGA

CAACTACTCATGGCGCCTGCTGCCGGCCCAGAACTCGTCCTTCTCACTCTCGAGCTACCCCATGCTGAGC

ACCCTGAGCGACCTGGACCAGAGCGCGATCTGCTCGCTGGCCAAGACGGAGAGGGAGCCGCTGTCCTTCT

TCGGCGTGGGCGGCGGCTTCGACGACGACGAGTCGGCGGTGAAGCAGGAGAACCAGACGCTGCGGCCCTT

CTTCGACGAGTGGCCCAAGGACAGGGACTCGTGGCCGGAGCTGCAGGACCATGACTCCAACCACAACAAT

GAGGCCTTCTCGGCCACCAAGCTGTCCATCTCCATCCCGGTGACCAGCTCCGATTTCTCCACCACCGCCG

GCTCCCGCTCGCCCCACGGTATATACTCCCGGTGA

>TaGRF1-2B

ATGGCGGCGGAGGGGGAGGACAAGAAGGATGCTAATTCCGTTGGAGGCGGCGGCGGCGAGAACACCCTGG

AGTCGGCGGAGGAGGCGATTCTGCAGGCGGTAGGGCAAGAGCCCGGTCAAGAATTGGAGGGCGAGGCGGA

GGAGAGCGCAGATCGAGAGGGGAACGGCGACGACGCTGGGAAGGAAGATAGTGGGTGTAAAGATCTGGTC

CTGGTAGAGGACCCTGTCCTGGTCGAGGATCCAGAGGAAGCATTTCAGGAAGAAATGAGAGCGCTTTTGG

CGTCTGTCCCTGAAGGTGCCGGGGCATCATTTACTGCGATGCAGCTGCAGGAGCTAGAGCAGCAGTCTCG

GGTATACCAGTATATGGCTGCCCGTGTGCCTGTGCCTACCCATCTCGTCTTCCCCATCTGGAAGAGTGTT

ACCGGTGCATCCTCTGAAGGCGCGCAGAATTACCCTACATTGATGGGATTGGCAACACTCTGCTTGGACT

TTGGGAAGAGCCCAGAACCAGAACCAGGAAGGTGCCGGCGAACAGATGGAAAAAAGTGGCGGTGCTGGAG

AAAAACAATCCCAAACGAGAAATATTGTGAACGCCATATGCACCGTGGTCGCAAGCGTCCTGTACAGGTT

ATTGTTGAGGATGACGAGCCTGATTCCGCATCAGGGTCAAAATCATCATCTGGCAAAGTCACTGAAGGAG

GCAAGAAGACTGACGACAAGAGTTCAAGTAGCAAGAAGCTTGCAGTGGCAGCACCAGCTGCTGTGGAGTT

TACATGA

>TaGRF2-2B

ATGACCGAGCGAAGGCAGGAACACTCGCCGCCGTCCAAGCTCCCCCGCCTCTCCGGCCCCGACGCCGACG

ACAACGACGGCGCAGGGACGGTGACCATGGCGGCGCCGTCGCCGCTGGTTCTTGGGCTGGGTCTCGGCGT

AGGCGGCAGCAGCAGTGACAGTGGACGTGGCGACGCGGAGGCATCTGCGGCGACGCGGCCATCGGCGCTG

ACGTTCATGCAGCGGCAGGAGCTGGAGCACCAGGTGCTCATCTACCGCTACTTCGCCGCCAACGCTCCCG

TGCCCGTGCACCTCGTCCTCCCCATCTGGAAGAGCGTCGCCGCCTCCTCCTCCGCCCCGCAGAGGTTCCC

ATCCCTGGCGGGACTGGGGAGCATGTGCTACGACCACAGGAGCAGCATGGAGCCGGAGCCGGACCGGTGC

CGGCGCACGGACGGCAAAAAGTGGCGGTGCTCGCGCGGCGTGATGCCGGGGCACAAGTACTGCGAGCGCC

ACGTCCACCGCGGCCGCGGCCGTGCAAGAAAGCCTGTGGAAGCCGCGCCGGCCACATCAGCCATCCCGAT

CCGCGCAATGCACGCCGCCGACGCGCAGGGCGCCACAAGCGCGCACGCGGCGCCACCGCAGCGCCTCGGC

TTCTCCTCCCCCGCCGGCGTCTACCTGGCCCACGGCACCGCCCGTGCCACCTGA

>TaGRF3-2B

ATGGCGATGCCCTTTGCCTCCCTGTCGCCGGCAGCCGACCACCACCGCTCCTCCCCCATCTTCCCCTTCT

GCCGCTCCTCCCCTCTCTACTCGGTAGGGGAGGAGACGGCGCATCAGCAGCAGCAGCAGCACACGATGAG

CGGCGCGAGGTGGGCGGCGAGGCCGGCGACCTTCACGGCGGCGCAGTACGAGGAGCTGGAGCAGCAGGCG

CTCATCTACAAGTACCTCGTCGCCGGCGTCCCCGTCCCGCCGGATCTCCTCCTCCCCATCCGCCGCGGCT

TCGACTCCCTCGCCTCGCGCTTCTACCACCACCACGCCCTCGGGTACGGTTCCTACTTCGGGAAGAAGCT

GGATCCGGAGCCGGGGCGGTGCCGGCGGACGGACGGCAAGAAGTGGCGGTGCTCCAAGGAGGCCGCCCAG

GACTCCAAGTACTGCGAGCGCCACATGCACCGCGGCCGCAACCGTTCAAGAAAGCCTGTGGAAACGCAGC

TCGTCGCCACGCCCCACCACTCCCACTCCCAGCAGCTGCAGCAGCACGCCCCCGCCGCCAGCGCCGCCGC

GTTCCACAGCCACTCGCCGTATCCGGCGATCGCCTCTGGCGGCGGCGGCTCCTTCGCCGTGGGATCTGCT

CAGCTGCACATGGACAATGCTGCTTCGCCTTACGCGACCGCTGGTGCCGCCGGAAACAAAGATTTCAGGT

ATTCTGCCTATGGGTTTAGGACTTCGGCGATGGAGGAGCACAACCAGTTCATCTCTGCGGCCATGGAGAC

CGCCATGGAGAACTACTCATGCCGCCTGATGCCGGCCCAGAACTCATCCTTCTCACTCGCCAGCTACCCC

ATGCTGGGCACCCTGGGCGACCTTGACCAGAGCGCGATCTGCTCGCTGGCCAAGACGGAGAGGGAGCCTC

TGTCCTTCTTCGGCGGCGGCGGCGGCTTCGACGACGACGACTCGGCGGTGAAGCAGGAGAACCAGACGCT

GCGGCCCTTCTTCGACGAGTGGCCCAAGGACAGGGACTCGTGGCCGGAGCTGCAGGACCACGATGCCAAC

AACAGCAGCAACGCCTTCTCGGCCACCAAGCTGTCCATCTCCATCCCGGTGACCAGCTCCGACTTCTCCA

CCACCGCCGGCTCCCGCTCGCCCAACGGTATATACTCCCGGTGA

>TaGRF1-2D

ATGGCGGCGGAGGGGGAGGACAAGAAGGATGCTAATTCCGTTGGAGGCGGCGGCGGCGGCGAGAACACCC

TGGAGGCGGCGGAGGAGGCGATTCTGCAGGCGGTAGGGCAAGAACCCGGTCAAGAATTGGAGGGCGAGGC

GGAGGAGAGCGCAGATCGAGAGGGGAACGGCGACGACACTGGGAAGGAAGATAGTGGGTGTAAAGATCTG

GTCCTGGTAGAGGACCCTGTCCTGGTCGAGGATCCAGAGGAAGCGGCAGCAACTGCAGCACTTCAGGAAG

AGATGAGAGCGCTTTTGGCGTCTGTCCCTGAAGGTGCTGGGGCATCATTTACTGCGATGCAGCTGCAGGA

GCTAGAGCAGCAGTCTCGGGTATACCAGTATATGGCTGCCCGTGTGCCTGTGCCTACCCATCTCGTCTTC

CCCATCTGGAAGAGTGTTACCGGTGCATCCTCTGAAGGCGCGCAGAATTACCCTACATTGATGGGATTGG

CAACACTCTGCTTGGACTTCGGGAAGAGCCCAGAACCAGAACCAGGAAGGTGCCGGCGAACAGATGGAAA

AAAGTGGCGATGCTGGAGAAAAACAATCCCAAACGAGAAATATTGTGAACGCCATATGCACCGTGGTCGC

AAGCGTCCTGTACAGGTTATTGTTGAGGATGACGAGCCTGATTCCGCATCAGGGTCAAAATCGTCATCTG

GCAAAGTCACTGAAGGAGGCAAGAAGACTGAAAACAAGAGTTCAAGTAGCAAGAAGCTTGCAGTGGCAGC

ACCAGCTGCTGTGGAGTTTACATGA

>TaGRF2-2D

ATGACCGAGCGAAGGCAGGAGCACTCGCCGCCGTCCAAGCTCCCCCGCCTCTCCGGCCCCGACGCCGACG

CCGACGACAATGATGGCGCAGGGACGGTGACCATGGCGGCGCCGTCGCCGCTGGTTCTTGGGCTGGGTCT

CGGCGTAGGCGGCAGCTGCAGTGACAGCGGACGCGGCGACGCGGAGGCCTCTGCGGCGACGCGGCCGTCG

GCGCTGACGTTCATGCAGCGGCAGGAGCTGGAGCACCAGGTGCTCATCTACCGCTACTTCGCCGCCAACG

CTCCCGTGCCGGTGCACCTCGTCCTCCCCATCTGGAAGAGCGTCGCCGCCTCCTCCTCCGCCCCGCAGAG

GTTCCCATCCCTGGCGGGACTGGGGAGCATGTGCTACGACCACAGGAGCAGCATGGAGCCGGAGCCGGAC

CGGTGCCGGCGCACGGACGGCAAGAAGTGGCGGTGCTCGCGCGGGGTGGTGCCGGGGCACAAGTACTGCG

AGCGCCACGTCCACCGCGGCCGCGGCCGTGCAAGAAAGCCTGTGGAAGCCGCGGCGGCCACATCAGCCAT

CCCGATCCGCGCGATGCACGCCGCCGACGCGCAGGGCGCCACCAGCGCGCACGCGGCGCCGCCGCAGCGC

CTCGGCTTCTCCTCCCCCGCCGGCGTGTACCTGGCCCACGGCACCGCCCGTGCCACCTGA

>TaGRF3-2D

ATGGCGATGCCCTTTGCCTCCCTGTCGCCGGCAGCCGACCACCACCGCTCCTCCCCCATCTTCCCCTTCT

GCCGCTCCTCCCCTCTCTACTCGGCAGGGGAGGAGGCGGCGCAGCAGCAGCAGCAGCACGCGATGAGCGG

CGCGAGGTGGGCGGCGATGAGGCCGGCGACCTTCACGGCGGCGCAGTACCAGGAGCTGGAGCAGCAGGCG

CTCATCTACAAGTACCTCGTCGCCGGCGTGCCCGTCCCGCCGGATCTCCTCCTCCCCATCCGCCGCGACT

TCGACTCCCTCGCCTCGCGCTTCTACCACCACCACGCCCTTGGGTACGGGTCCTACTTCGGGAAGAAGCT

GGATCCGGAGCCGGGGCGGTGCCGGCGGACGGACGGCAAGAAGTGGCGGTGCTCCAAGGAGGCCGCCCAG

GACTCCAAGTACTGCGAGCGCCACATGCACCGCGGCCGCAACCGTTCAAGAAAGCCTGTGGAAACGCAGC

TCGTCGCCACGCCCCACTCCCACTCCCACTCCCAGCAGCTGCAGCAGCACGCCCCCGCCGCCACCGCCGC

CGCGTTCCACAGCCACTCGCCGTACCCGGCGATCGCCACTGGCGGCGGCGGCGGCGCGGCCGGCTCCTTC

GGCCTGGGGTCTGCTCAGCTGCACATGGACAATGCTGCTGCGCCTTACGCGACCGCTGGTGCGGCCGGAA

ACAAGGATTTCAGGTATTCTGCCTATGGGTTTAGGACTTCGGCGCTGGAGGAGCACAACCAGTTCATCAG

CGCGGCCATGGACACCGCCATGGACAACTACTCATGGCGCCTGATGCCGGCCCAGAACTCGGCGTTCTCA

CTCTCGAGCTACCCCATGCTGGGCACCCTGGGCGACCTGGACCAGAGCGCGATCTGCTCGCTGGCCAAGA

CGGAGAGGGAGCCGCTGTCCTTCGGCGGCGGCGGCGGCTTCGAGGACGACGAGTCGGCGGTGAAGCAGGA

GAACCAGACGCTGCGGCCCTTCTTCGACGAGTGGCCCAAGGACAGGGACTCGTGGCCGGAGCTGCAGGAC

CATGACTCCAACCACAACAGCAACGCCTTCTCGGCCACCAAGCTGTCCATCTCCATCCCGGTGACCAGCT

CCGACTTCTCCACCACCGCCGGCTCCCGCTCGCCCCACGGTATATACTCCCGGTGA

>TaGRF4-4A

ATGGACCTGGGCGGGGTGCTGATGGCGGCCGCGGACGCGGGGGTGGGCGGCGGGGAGCTCGGCATGCTCG

GATCTAGGCTGCTCAAGCACGGGAGGGGCAATGCCGCGGCGGCGGAGGCCGACGAGCGTCACGAGCACGG

GTGGGGCGGCGGCAGGCCGGCCGCCAAGCAGGCCCGGGTCGCCGGGGACAGCGACGCCGTGTCCGAGGCC

GTCAAGGCGGCCGCGCCATACCTGCTGGGCACCTGCAGCCCCGGGCACGGCCGGGAGAAGATGCTCAGCT

TCTCCTCCTCGCAGCAGCCGCCCTCCTGCCCCTCCGCCGCCGCCGCCGCTCAGGCCGCGCTGCCGCTCTA

CTACGGCACGCCCGCTTCTTGCTTAGGGTTGAGCTCGGTGAGCTTGAGCGCCAGCATCCAGGGCGCCATG

GCCAGGGTGAGGGGGCCCTTCACGCCGTCGCAGTGGATGGAGCTGGAGCACCAGGCCCTGATCTACAAGT

ACCTGGCGGCCAACATCGCCGTGCCGCACAGCCTCCTCGTCCCCATCCGCCGGAGCGTCACCTCGCTCTA

CCCGTCCGCCTACTTTGGCTCCTCCACATTGGGGTGGGGGCCTTTCCAGCTGGGCTACTCCGGGAGCGCG

GACCTGGAGCCCGGGCGGTGCCGCCGGACGGACGGCAAGAAGTGGCGGTGCTCCAGGGACGCCGTCGCTG

ACCAGAAGTACTGCGAGCGGCATATGAACCGGGGACGCCATCGTTCAAGAAAGCATGTGGAAGGCCAGCC

TGGCCATGCCGCGAAAGCGATGCCTGCGACGGTGGCGGCTGCTGCCGCCCAGCCCGGTGCTCTCGCCACC

GGGGGCGGCGGCGGAGCCACCGCCGGCGCCGCCATCTGCCACGAGCAGCAGCCGTTGAAGAGCTACTCCG

CCAGCACCATTGATCCTTGTTCACTGCAATACAACAGGGAAATGGCGAGCAAGCAGCAACACGAGTGCGA

GCAAGTGCAGGACTCGGACACCCTCTCGATGCTGACCTCCATGAGCGCGAGGAACACCAACACGGGCAGC

ATGTTCCCGTTCTCAAAGGAACATCATAACCACAATCCTTTCGAGGTGACGAGCTCGAGGCCGGACTACG

GGCTGGTTTCCTCCGACTCGCTGATGAGCTCCCCCCACAGCTCCCTGGAGAACGTCAACCTGCTCACCTC

GCAGCGAGCGCTCTCGAGCGAGCAGCAGAGCTCGCTCTCCCTGCAGCACTTCGCGGACTGGCCGAGGACG

CCGTCGCAGCAGGGGCAGGGAGGGGGAGGCCTCTCATGGCCGGACGCCGAGAACATGCAGCTGGCTCATC

AGCGGACCCAGCAGCTCTCGGTGTCCGCCGCTCCGATGGCGTCCTCCGACCTGTCGTCGGCCTCCACGTC

CCCCATCCACGAGAAGCTCATGCTGTCGCCCCTCAAGCTGAGCCGCGAGTACAGCCCCATCGGCCTCAGC

GTCGCGGCCACGGCGGCAGCGGCGGCGAAGGACGAGGGGGAGGCGAACTGGATGCCCATGTTCCGCGACT

CGTCCATGGGCGGGCCACTGGGGGAGGCTCTGAACAAGAACAATGGCGGCAACATGGAGGCCAAGAACTA

CCTGTCGGCGTCGCTGAACCTCATGACGGACGCCTGGGACTCGAGCCCGCTGGAGTCGTCGCCGGTGGGG

GTCCTGCAGAGGACCGCCTTCGGGTCGGTGTCCAGCAGCACCGGCAGCAGCCCCAGGCAGGAGTACCACG

GCGTGTATGATGGTAATCCGCGGGATGATCTCGGCTCCATCGTCGTGAATCACCCCAGCATCCGCCTCAT

GTGA

>TaGRF5-4A

ATGGAGCTCGGGCAGGTGCTGGGCTACACGCCACCGGCGACCAAGGACGCGAGATCCGGCGGCGGCTTCA

CCCAGGCTGCCGCTTGCCCCTACCCCTACCCCTCCCCCTTCCTCGACGAGCAGAAGATGCTCAGCTTCTC

CAAGGCCGCCGCCGCTCACCAGCCGCCCTCAGGTATGGATTTTGGGAGGTCCAATGAGCAGAGGCTGTTG

CTGGCCAGGAGCAAGATGCCCTTCACCCCTTCACAGTGGATGGAGCTGGAGCACCAGGCCCTCATTTACA

AGTATCTCAATGCAAAGGCCCCCATACCTTCCGGCCTGCTCATCTCCATCAGCAAGAGCTTCAGACCCTC

CTCCGATAGAATGCCCTGGAGGCCTGTCTATCAAGGGTTCACCAATGCAGATTCTGACCCGGAACCTGGA

AGATGCCGTCGAACAGACGGCAAGAAATGGCGGTGCTCAAAGGAGGCGATGGCCGAGCACAAGTACTGTG

AGCGGCACATCAATAGGAACCGCCATCGTTCAAGAAAGCCTGTGGAAAACCAAACAAGGAAGAACGCCAA

AGAGACACCTGCTGCTGGCTCGATATCGGCCGCTGTCTCACAGGGTGGCTGTAAGAAAGCAAAAGCTGGT

GATGAACTGAAGCCAGGGAGCGTCAGTTATTGGACAGATAATTTAAACAGGGCAATGGTGAGCAAAGCCA

GGGGAAACAACCCTGAAGAAGGCAACAGTGCTCCACTCCTGAATTCTACTAATCAACAACACACATTGTC

CTTGTTCTCTCAACTGAAGCAACAGAGCAAACCAGATAAGTTCAGCCCGGCAGTCGATAGTGAATCGATC

TCCTCAAATACTGTATTGAAGCCTTGGGAAAGAAGCAACCAGCAGAGCAGTAAGGACGTTTCTTCCACGA

CGCTCCATGATCGCGGGTGCCTTCAATCAGTCCTTCAAGATTTCAGCATGCATAAGAATGACAAGATCGA

GTCTCAGAAAAACAATGCTTCAGTGCCATCTACTTTCTATTCATCTACAGAAGGTCGACACATCAGCTGC

CTTGCATCTAACATGATGCAAGTGCAGGAGGATTGCATCTCAAGCTCTTGGGAGATACCTCAAGGTGGGC

CTTTAGGTGAAATCCTAACAAACTCCAAGAATACTGATGACTTGACCAATAAGTGTGAATCAAGATCATA

TGGTTGGTTACTGAGTCTTGATGAACATGAAATGTGA

>TaGRF6-4A

ATGCTGAGCTCGTCGGCGGCGATGGGGATGGGGCTGGGGGGCTACGGCCAGCAGCAGCAGCAGCAGATGC

AGATGCAGATGCAGCGGGGCGCGGGGCCGGTGTTCACGCCGGCGCAGTGGGCCGAGCTGGAGCAGCAGGC

GCTGATTTACAAGTACCTCATGGCAGGCGTGCCCGTGCCGCCCGATCTCCTGCTCCCCATCCGCCCCCAC

CACCCCGCCGCCGGCGCCGCCGGAACCACCTTCTCCTTCGCCAGCCCCGCCGCCTCGCCCTTCTACCACC

ACCACCATCCCTCCATGAGTTACTACGCCTACTATGGCAAGAAGCTCGACCCGGAGCCGTGGCGGTGCCG

GCGCACCGACGGCAAGAAGTGGCGGTGCTCCAAGGAGGCGCACCCCGACTCCAAGTACTGCGAGCGCCAC

ATGCACCGTGGCCGCAACCGTTCAAGAAAGCCTGTGGAATCCAAGTCTGCTTCCCCTGCGCACCAGTCGC

AGCAGCCCCCGCTGTCCGCCGTCACGTCCGCGGCCCGCGACGCCGAGCCGCTCCCCTCCCTCCCGGCTGG

GGCTAAAACCCATGGCCTGTCCCTCGGCGGGGCTGGCTCGTCGCAGATGCACGTCGACGCCTCATCATAC

GGCGGCAAATACTCCCTTGGAGCTAAATCTGATGTGGGTGAACTGAGCTTCTTCTCTGGAGCATCAGGAA

ACAACAACAGGGGCTTCACCATCGATTCCCCAACGGACAGCTCGTGGCACTCGATGGGGTCCAGCCTGCC

CCCGTACCAACTGTCGAAACCTAGAGATTCCGGCCTCATGCAAGGCGGCTTCTCGTATTCCCACTATGAG

CCGTCGCAGGAGCTTGGGCAGGTAACCATCGCCTCGCTGTCCCACTCCCAGGAGCAGGACTGCCGCTCTT

TGGGTGGTGGAGGTGGAGGAGGTGGAGGTGGAGGTGGAGGGCTCATGGGAAATGTCAAGCAGGAGAACCA

GCCGCTGAGGCCCTTCTTCGACGAGTGGCCGGGGAGGCGGGACTCGTGGTCGGAGATGGACGACGAGCGC

TCCAACGGCACCTCCTTCTCGACGACCCAGCTCTCGATCTCCATCCCGATGCCTCGATGCGATTGA

>TaGRF4-4B

ATGGACCTGGGCGGCGTGCTGATGGCGGCCGCGGACGCGGGGGTGGGCGGCGGGGACCTGGGCATGCTCG

GATCTAGGCTGCTCAAGCACGGGAGGGGCAATGCGGCGGCGGCGGAGGCCGACGAGCACGGCTGGGGCAG

CGGCAGGCCGCCCGCCAAGCAGGCCCGGGTCGCGGCCTCGGCCGCGTCCGGGGACAGCGACGCCGTGTCC

GAGGCCGTCAAGGCGGCGGCACCCTACCTGCTCGGCACCTGCAGCCCCGGGCACGGCCGGGAGAAGATGC

TCAGCTTCTCCTCCTCGCAGCCGCCCTCCTGCCCCTCCTCCGCCGCCGCCGCCGCCGCTCAGGCCGCGCT

GCCGCTCTACTACGGCACGCCCGCTTCTTGCTCAGGGTTGAGCTCGGTGAGCTTGAGCGCCAGCATCCAG

AACGCCATGGCCAGGGTGAGGGGGCCCTTCACGCCGTCGCAGTGGATGGAGCTGGAGCACCAGGCCCTGA

TCTACAAGTACCTGGCGGCCAACATCGCCGTGCCTCACAACCTGCTCGTCCCCATCCGCCGGAGCGTCAC

CTCGCTCTACCCGTCCGCCTACTTTGGCTCCTCCACATTGGGGTGGGGGCCTTTCCAGCTGGGCTACTCC

GGGAGCGCGGACCTGGAGCCCGGGCGGTGCCGCCGGACGGACGGCAAGAAGTGGCGGTGCTCCAGGGACG

CCGTCGCCGACCAGAAGTACTGCGAGCGGCATATGAACCGGGGACGCCATCGTTCAAGAAAGCATGTGGA

AGGCCAGCCTGGCCATGCCGCGAAAGCGATGCCTGCGACGGTGGCGGCGGCTGCTGCCCAGCCCGGTGCT

CTCGCCACCGGGGGCGGCGGCGGAGCTACCGCCGGCGCCGCCGCCATCTGCCACGAGCAGCAGCCGTTGA

AGAACTACGCCGCGAACACCATTGATCCTTGTTCACTGCAATATAACAGGGAAATGGTGAGCAAGCAGCA

GCAACACGAGTGCGAGCAAGTGCAGGACTCCGACACCCTCTCGATGCTGACCTCCATGAGCGCGAGGAAC

ACCAACACGGGCAGCATGTTCCCGTTCTCAAAGGAGCATCACAATCACAATCCTTTCGAGGTGACGAGCT

CAAGGCCGGACTACGGGCTGGTTTCATCCGACTCGCTGATGAGCTCCCCTCACAGCTCCCTGGAGAACGT

CAACCTGCTCACCTCGCACTCGCAGCGAGCGCTCTCCAACGAGCAGCAGAGCTCGCTCTCCCTGCAGCAC

TTCGCGGACTGGCCGAGGACGCCCTCGCAGCAGGGGCAAGGAGGAGGAGGTCTCTCATGGCCGGACGCCG

AGGACATGCAAGCACATCAGAGGACCCAGCTCTCGGTGTCCGCCGCTCCAATGGCGTCCCCCGACCTGTC

GTCGGCCTCCACGTCCCCGATCCACGAGAAGCTCATGCTGTCGCCCCTCAAGCTGAGCCGCGAGTACAGC

CCCATCGGCCTCAGCATCGCGGCGACGGCGGCGGCGGCGAAGGACGAGGGGGAGGCGAACTGGATGCCCA

TGTTCCGCGACTCGTCCATGGGCGGGCCGCTGGGGGAGGCCCTGAACAAGAACAATGGCGGCAACATGGA

GGCCAAGAACTACCTGTCGGCGTCGCTGAACCTCATGACGGACGCCTGGGACTCGAGCCCGCTGGAGTCG

TCCCCGGTGGGGGTCCTGCAGAGGACCGCCTTCGGGTCGGTGTCGAGCAGCACCGGCAGCAGCCCCAGGC

AGGAGTACCACGGCGTGTATGATGGTAACCCGCGGGATGATCTCGGCTCCATCGTCGTGAATCACCCCAG

CATCCGCCTCATGTGA

>TaGRF5-4D

ATGCTTCCTGAGCTCACCGCCGCCGCCATGGAGCTCGGGCAGGTGNNNNNNTACACGCCGCCGGCGACCA

AGGACGCGAGATCCGGCGGCGGCTTCGCCCAGGCCGCCGCTTGCCCCTACCCCTACCCCTCCCCCTTCCT

CGACGAGCAGAAGATGCTCAGCTTCTCCAAGGCCGCCGCCCCTCCATCGTCAGGTATGGATTTTGGCAGG

TCCAATGAGCAGAGGCTGCTGCTGGCCAGGAGCAAGATGCCCTTCACTCCTTCACAGTGGATGGAGCTGG

AGCACCAGGCCCTCATATACAAGTATCTCAATGCAAAGGCCCCCATACCTTCCAGCCTGCTCATCTCCAT

CAGCAAAAGCTTCAGACCCTCCTCCGATAGAATGCCCTGGAGGCCTGTCTACCAAGGGTTCACCAATGCA

GATTCTGACCCGGAACCTGGAAGATGCCGTCGAACAGACGGCAAGAAATGGCGGTGCTCAAAGGAGGCGA

TGGCCGAGCACAAGTACTGTGAGCGGCACATCAATAGGAACCGCCATCGTTCAAGAAAGCCTGTGGAAAA

CCAAACAAGGAAGAACGCCAAAGAGACGCCTGCTGCTGGCTCGTTATCGGCCGCTGTCTCACAGGGTGGC

TGTAAGAAAGCAAAAGCTGGTGATGAACTGAAGCCAGGGAGCGTCAGCTATTGGACAGATAATTTAAACA

GGGCAATGGTGAGCAAAGCCAGGGGAAACAACCCTGAAGAAGGCAACAGTGCTCCACTCCTGAATTCTAC

TAATCAACAACACACATTGTCCTTGTTCTCTCAACTGAAGCAACAGAGCAAACCAGATAAGTTCAGCCCG

GCAGTCGATAGTGAATCGATCTCCTCAAATACAGTATTGAAGCCCTGGGAAAGAAGCAACCAGCAGAGCA

GCAAGGACGTTTCTTCGACGACGCTCCATGATCGCGGGTGCCTTCAATCAGTCCTTCAAGATTTCAGCAT

GCATAAGAATGACAAGATCGAGGCTCAGAAAAACAATGCTTCAGTGCCATCAACTTTCTATTCACCTACA

GAAGGTCAACACATCAGCTGCCTTGCATCTAACATGATGCAAGTGCAGGAGGATTGCATCTCAAGCTCTT

GGGAGATACCTCAAGGTGGGCCATTAGGTGAAATCCTAACAAACTCCAAGAACACTGATGACTTGACCAA

TAAGTGTGAATCAAGATCATATGGTTGGTTACTGAGTCTTGATGAACATGAAATGTGA

>TaGRF4-4D

ATGGACCTGGGCGGGGTGCTGATGGCGGCCGCGGACGCGGGGGTGGGCGGCGGGGACCTCGGCATGCTCG

GATCTAGGCTGCTCAAGCACGGGAGGGGCAATGAGGCCGACGAGCACGGCTGGGGCGGCGGCAGGCCGGC

GTCCAAGCAGGCCCGGGTCGCCGGGGACAGCGACGCGGTGTCCGAGGCCGTCAAGGCGGCCGCGCCCTAC

CTGCTCGGCACCTGCAGCCCCGGGCACGGCCGGGAGAAGATGCTCAGCTTCTCCTCCTCGCAGCCGGCCT

CCTGCCCCTCCGCCGCTCAGGCCGCGCTGCCGCTCTACTACGGCACGCCCGCTTCTTGCTCAGGGTTGAG

CTCAGTGAGGGGGCCCTTCACGCCGTCGCAGTGGATGGAGCTGGAGCACCAGGCCCTGATCTACAAGTAC

CTGGCGGCCAACATCGCCGTGCCTCACAACCTCGTCGTCCCCATCCGCCGGAGCGTCACCTCGCTCTACC

CGTCCGCCTACTTTGGCTCCTCCACATTGGGGTGGGGGCCTTTCCAGCTGGGCTACTCCGGGAGCGCGGA

CCTGGAGCCCGGGCGGTGCCGCCGGACGGACGGCAAGAAGTGGCGGTGCTCCAGGGACGCCGTCGCCGAC

CAGAAGTACTGCGAGCGGCATATGAACCGGGGACGCCATCGTTCAAGAAAGCATGTGGAAGGCCAGCCTG

GCCATGCCGCGAAAGCGATGCCTGCGACGGCCGCTGCTGCCGCCCAGCCCGGTGCTCTCGCCACCGGGGG

CGGCGGCGGAGCTCCCGCCGGCGCCGCCATCTGCCACGAGCAGCAACCGTTGAAGAACTACGCCGCCAGC

ACCATTGATCCTTGTTCACTGCAATATAACAGGGAAATGGTGAGCAAGCAGCAACACGAGTGCGAGCAAG

TGCAGGACTCCGACACCCTCTCGATGCTGACCTCCATGAGCGCGAGGAACACCAATGCAGGCAGCATGTT

CCCGTTCTCAAAGGAACATCATAACCACAATCCTTTCGAGGTGACGAGCTCGAGGCCGGACTACGGGCTG

GTTTCATCCGACTCGCTGATGAGCTCCCCTCACAGCTCCCTGGAGAACGTCAACCTGCTCACCTCGCAGC

GAGCTCTCTCGAGCGAGCAGCAGAGCTCGCTCTCCCTGCAGCACTTCGCGGACTGGCCGAGGACGCCCTC

GCAGCAGGGGCAGGGAGGAGGTCTCTCATGGCCGGACGCCGAGGACATGCAAGCTCATCAGAGGACCCAG

CTCTCGGTGTCCGCCGTTCCAATGGCGTCCTCTGACCTGTCGTCGGCCTCCACGTCCCCGATCCACGAGA

AGCTCATGCTGTCGCCCCTCAAGCTGAGCCGCGAGTACAGCCCCATCGGCCTCAGCGTCGCGGCCACGGC

GGCGGTGGCGAAGGACGAGGGGGAGGCGAACTGGATGCCCATGTTCCGCGACTCGTCCATGGGCGGGCCG

CTGGGGGAGGCTCTGAACAAGAACAATGGCGGCAACATGGAGGCCAAGAGCTACCTGTCGGCGTCGCTGA

ACCTGATGACGGACGCCTGGGACTCGAGCCCGCTGGAGTCGTCGCCGGTGGGGGTCCTGCAGAGGACCGC

CTTCGGATCGGTGTCCAGCAGCACCGGCAGCAGCCCCAGGCAGGAGTACCACGGCGTGTATGATGGTAAC

CCGCGGGATGATCTCGGCTCCATCGTCGTGAATCACCCCAGCATCCGCCTGATGTGA

>TaGRF7-6A

ATGAAAAAACTGGAGCTGGACGACGGCATGGTTGCCGGCAATGGCGACGCCGGACAGCGCCCGGGCGTGG

CAACTTTGATGCCACCCGCTGCTACTCATGTTCCTGTTGCCTCCACCATGGGTGCCGCCGGGGGCTTATT

CACGGCGACTCAATGGGTGGAGCTGCAACGCCAGTCGCTGATCTACAACCACATGGCCGCATCGTTGCCC

ATCCCCTCATACCTCCTCTTCTCAAATATCAACCCGGCAGCCGCCGCCGCCGCGGCGGCGGCGCCTTCCC

AAGCTGCACCGCCGTACTACTGCTGCTACAACACCCCCCTGCTTGTCCACCATTACCAGGCCCAGCAGGC

AGCGCAGATGTCCATGCTGCTGCAGTGCATGCAGCAGGCCGTCGCGCGGAGGAGGTGCGGCCGTACAGAC

GGCAAGAAGTGGCGCTGCGCCAGGGACGCCGAGCCCGACCAGAAGTACTGCCAACGCCACCTGAACCGCG

TCGGCCGGGCACGACCCCCTCCCTCTGCGAGAAAGCAACAGCACCAGCATGCAGCTGCTGCGGTGGTTGC

TCATCATCGTGACCGGGACAAGTCCGCCATGACCGCCCCTGCGGCCATTCATACCAGTCATGGTAACAAG

TCTTCCGGGAATACGATGCTGCGGGAGGATGACGACGACTACTCGCGAGGCCTCTTGGACTTCACCGGTG

GAGTTTGCCTCGCGGAGCAGCGAGAGAACCGGCTCAGCCTCAACTACGACAACATTGCCGAGCTCTACTG

CAATAGGCAGGTGACCGCCACGCCCACGGCCACAGCATCGGCGGCGGCGTCAGCAACGGCCATGGACGAC

GACGCCATTCATCATGGGGCAGCAGCAACCTGGGTGGGCATCGGGGGACCCCTCGGCGAGGCGCTCGGTC

TCGCCGTAGAGATTCAGTGGCCTGCTGGCTCCACCTAG

>TaGRF8-6A

ATGGCCGACGAGAAAGAAGCCGACTCGCTGCAGCCGCCGTCCAAGCAGCCCCGCCTCTCCTCCGCCGACT

CGAACGCCGGGGCGGTGACGATGGCGGTCTCGTCGCCGCTGGGTCTTGGCCTCGGCCTGGGGCTCGGTGG

CGATAGCCGTGGCGAGCAGCAGGCCTTCGAAGCACGGGCGGCGGCGAAGTCGGCGCTGACGTTCATGCAG

CAGCAGGAGCTGGAGCACCAGGTGCTCATCTACCGCTACTTCGCGGCGGGGGCGCCCGTGCCGGTGCACC

TCGTGCTGCCCATCTGGAAGAGCGTCGCCGCCTCCTCCTTCGGCCCGCACCGCTTCCCCTCCCTGATTGG

GCTGGGGAGCCTGTGCTTCGACTACCGGAGCAGCATGGAGCCGGAGCCCGGGCGGTGCCGCCGCACGGAC

GGCAAGAAGTGGCGGTGCTCCCGCGACGTGGTGCAGGGGCACAAGTACTGCGAGCGGCACGTCCACCGGG

GCCGCGGCCGTTCAAGAAAGCCTGTGGAAGGAGCCCCCTCAGCCCGGGCGCACAGCGACAGCACCGCCAC

CGCCCCGCCCTGCGCCATCGGCTTCTCCCCCGCCGGCATCCTCCACGCCACCCACAGCGCCGCCGCGCGC

GCCACCTGA

>TaGRF9-6A

ATGGCGATGCCGTATGCCTCTCTTTCCCCGGCAGGCGACCGCCGCTCCTCCCCGGCCGCCACCGCCACCG

CCTCCCTCCTCCCCTTCTGCCGCTCCTCCCCCTTCTCCGCCGGCGGCAATGGCGGCATGGGGGAGGAGGC

GCCGATGGACGGGAGGTGGATGGCGAGGCCGGTGCCCTTCACGGCGGCGCAGTACGAGGAGCTGGAGCAC

CAGGCGCTCATATACAAGTACCTGGTGGCCGGCGTGCCCGTCCCGCCGGATCTCGTGCTCCCCATCCGCC

GCGGCATCGAGTCCCTCGCCGCCCGCTTCTACCACAACCCCCTCGCCATCGGGTACGGATCGTACCTGGG

CAAGAAGGTGGATCCGGAGCCGGGCCGGTGCCGGCGCACGGACGGCAAGAAGTGGCGGTGCGCCAAGGAG

GCCGCCTCCGACTCCAAGTACTGCGAGCGCCACATGCACCGCGGCCGCAACCGTTCAAGAAAGCCTGTGG

AAACGCAGCTCGTGCCCCACTCCCAGCCGCCGGCCGCCTCCGCCGTGCCGCCCCTCGCCACCGGCTTCCA

CGGCCACTCCCTCTACCCCGCCGTCGGCGGCGGCACCAACGGTGGTGGAGGCGGGGGGAACAACGGCATG

TCCATGCCCGGCACGTTCTCCTCCGCGCTGGGGCCGCCTCAGCAGCACATGGGCAACAATGCCGCCTCTC

CCTACGCGGCTCTCGGCGGCGCCGGAACATGCAAAGATTTCAGGTATACCGCATATGGAATAAGATCTTT

GGCAGATGAGCAGAGTCAGCTCATGACAGAAGCCATGAACACCTCCGTGGAGAACCCATGGCGCCTGCCG

CCATCTTCTCAAACGACTACATTCCCGCTCTCAAGCTACTCTCCTCAGCTTGGAGCAACGAGTGACCTGG

GTCAGAACAACAGCAGCAACAACAACAGCGGCGTCAAGGCCGAGCGACAGCAGCAGCAGCAGCCGCTCTC

CTTCCCGGGGTGCGGCGACTTCGGCGGCGGCGACTCCGCGAAGCAGGAGAACCAGACGCTGCGGCCGTTC

TTCGACGAGTGGCCGAAGACGAGGGACTCGTGGTCGGACCTGACCGACGACAACTCGAACGTCGCCTCCT

TCTCGGCCACCCAGCTGTCGATCTCGATACCTATGACGTCCCCCGACTTCTCCGCCGCCAGCTCCCAGTC

GCCCAACGGCATGCTGTTCGCCGGCGAGATGTACTAG

>TaGRF10-6A

ATGATGATGATGGGCGGTCGCGCGGGGGCCGGCGGCGTCGGGGCAGGCGGCGGCCGGTGCCCGTTCACGG

CGACGCAGTGGCAGGAGCTGGAGCACCAGGCGCTCATCTACAAGTACATGGCCTCCGGCGTGCCCATCCC

CTCCGACCTCCTCCTCCCGCTCCGCCGCAGCTTCCTCCTCGACTCCGCCCTCGCCACCTCCCCCTCCCTC

GCCTTCCCTCCCCAGGCCGCACTTGGGTGGGGTTGCTTTGGCATGGGGTTCGGCCGGAAGGCGGAGGACC

CGGAGCCGGGGCGGTGCCGGCGGACGGACGGGAAGAAGTGGCGCTGCTCCAAGGAGGCGTACCCGGACTC

CAAGTACTGCGAGAAGCACATGCACCGGGGCAAGAACCGTTCAAGAAAGCCTGTGGAAATGTCCTTGGCC

ACGCCCCCGCCGCCGCCTTCCTCCTCGGCCTCCTCTTCCTCCTCCAACGTCCACTCCGCCGTCAACGTCG

CCACCACCACCACCTCCCCAGCGCCGTCCTACCACCGCCACGCCGCTGCGACTCACGACACGACGCCCTA

CCACGCCCTCTACGGCGGCCCCTACTCCTCCGCCGGCCGCCAGCAGCACGCCAGCGCCTACCACCACGCG

GCGCAGGTCAGCCCGTTCCACCTGCACCTCGACACCACCCACCCGCACCCGCCGCCGTCCTACTACTCCA

CCATGGACCACAGCAAGGACAGCTACGCCTACGGGCACAGCGTCAAGGAGGTGCACGGCGGCGGCGAGCA

CGCCTTCTTCTCCTCCGACGTCAGCACCGACAGGGACCACCACCACCATCAGCACCAACACCACGCTAGC

GCCGGCGGCAACGGCCAGTGGCAGTTCAAGCAGCTCGGCGGCATGGAGCCCAAGCAGCACAACCCCACGT

CGCTCTTCCCCGGCTACGGCAACAACGCGGCGTACGCCATCGACCTGTCCAGCAAAGAAGAGGACGAGGA

GAAGGAGAGGCGGCAACAGCAGCAGCACTGCTTCCTGCTGGGCGCCGACCTGAGGCTCGACAAGCCGTCG

TCGGGGCACGGCGACTCCGCCGACCAGAAGCCTCTCCGGCCGTTCTTCGACGAGTGGCCGCACGAGAAGA

CTGGCAGCAAGGGGTCGTGGATGGGGCTCGAGGGGGAGACGCAGCTCTCCATCTCCATCGCCAATGAACT

CCCCATCACCACCACCTCCCGCTACCACCATGGTGAATGA

>TaGRF8-6B

ATGGCCGACGAGAAAGAAGCCGACTCGCTGCAGCCGCCGTCCAAGCAGCCCCGCCTCTCCTCCGCCGACT

CGAACGCCGGGGCGGTGACGACGGCGGTCTCGTCGCCGCTGGGTCTTGGCCTCGGCCTGGGGCTCGGCGG

CGATAGCCGTGGCGAGCAGCAGGCCTTCGAAGCACGGGCAGCCGCGGCGGCGGCGAAATCGGCGCTGACG

TTCATGCAGCAGCAGGAGCTGGAGCACCAGGTGCTCATCTACCGCTACTTCGCGGCGGGTGCGCCCGTGC

CGGTGCACCTCGTGCTCCCCATCTGGAAGAGCGTCGCCGCCTCCTCCTTCGGCCCGCACCGCTTCCCCTC

CCTGATTGGGCTGGGGAGCCTGTGCTTCGACTACCGGAGCAGCATGGAGCCGGAGCCCGGGCGGTGCCGC

CGCACGGACGGCAAGAAGTGGCGGTGCTCCCGCGACGTGGTGCAGGGGCACAAGTACTGCGAGCGGCACG

TCCACCGGGGCCGCGGCCGTTCAAGAAAGCCTGTGGAAGGAGCCTCCGCAGCCCCGGCGCACAGCGGCAG

CCCCACCACAGCCCCGCCCCGCGCCATCGGCTTCTCCCCCGCCGGCATCCTCCACGCCACCCACAGCGCC

ACCTGA

>TaGRF9-6B

ATGGCGATGCCGTATGCCTCTCTTTCCCCGGCAGGCGACCGCCGCTCCTCCCCGGCCGCCACCGCCTCCC

TCCTCCCCTTCTGCCGCTCCTCCCCGTTCTCCGCCGGCAATGGCGGCATGGGGGAGGAGGCGCGGATGGC

CGGTAGGTGGATGGCGAGGCCGGCGCCCTTCACGGCGGCGCAGTACGAGGAGCTGGAGCACCAGGCGCTG

ATATACAAGTACCTGGTGGCCGGCGTGCCCGTCCCGCCGGATCTCGTGCTCCCCATCCGCCGCGGCATCG

AGACCCTCGCCGCCCGCTTCTACCACAACCCCCTCGCCATCGGGTATGGATCGTACCTGGGCAAGAAGGT

GGATCCGGAGCCCGGCCGGTGCCGGCGCACGGACGGCAAGAAGTGGCGGTGCGCCAAGGAGGCCGCCTCC

GACTCCAAGTATTGCGAGCGCCACATGCACCGCGGCCGCAACCGTTCAAGAAAGCCTGTGGAAACGCAGC

TCGTCTCGCACTCCCAGCCGCCGGCCGCCTCCGTCGTGCCGCCCCTCGCCACCGGCTTCCACAACCACTC

CCTCTACCCCGCCATCGGCGGCACCAACGGTGGTGGAGGCGGGGGGAACAACGGCATGCCCAACACGTTC

TCCTCCGCGCTGGGGCCTCCTCAGCAGCACATGGGCAACAATGCCTCCTCACCCTACGCGGCTCTCGGTG

GCGCCGGAACATGCAAAGATTTCAGGTATACCGCATATGGAATAAGATCTTTGGCAGACGAGCACAGTCA

GCTCATGACAGAAGCCATGAATACCTCCGTGGAGAACCCATGGCGCCTGCCGCCATCGTCTCAAACGACC

ACATTCCCGCTCTCAAGCTACGCTCCTCAGCTTGGAGCAACTAGTGACCTGGGTCAGAACAACAACAGCA

GCAGCAGCAACAGTGCCGTCAAGTCCGAACGGCAGCAGCAGCAGCAGCCCCTCTCCTTCCCGGGGTGCGG

CGACTTCGGCGGCGGCGGCGCCATGGACTCCGCGAAGCAGGAGAACCAGACGCTGCGGCCGTTCTTCGAC

GAGTGGCCCAAGACGAGGGACTCGTGGTCGGACCTGACCGACGACAACTCCAGCCTCGCCTCCTTCTCGG

CCACCCAGCTGTCGATCTCGATACCCATGACGTCCTCCGACTTCTCGGCCGCCAGCTCCCAGTCGCCCAA

CGGTATGCTGTTCGCCGGCGAAATGTACTAG

>TaGRF10-6B

ATGATGATGATGGGCGGTCGCGCGGGGGCCGGCGGCGTCGGGGCCGGCGGGGGCCGGTGCCCGTTCACGG

CGACGCAGTGGCAGGAGCTGGAGCACCAGGCGCTCATCTACAAGTACATGGCCTCCGGCGTGCCCATCCC

CTCCGACCTCCTCCTCCCGCTCCGCCGCAGCTTCCTCCTCGACTCCGCCCTCGCCACCTCCCCCTCCCTC

GCCTTCCCTCCCCAGGCCGCACTGGGCTGGGGATGTTTCGGGATGGGGTTCGGCCGGAAGGCGGAGGACC

CGGAGCCGGGGCGGTGCCGGCGGACTGACGGCAAGAAGTGGCGCTGCTCCAAGGAGGCGTACCCGGACTC

CAAGTACTGCGAGAAGCACATGCACCGCGGCAAGAACCGTTCAAGAAAGCCTGTGGAAATGTCCTTGGCC

ACGCCCCCGCCGCCGCCTTCCTCCTCGGCCTCCTCCTCCTCCTCCAACGTCCACTCCGCCGTCAACGCCG

CCACCACCACCACCTCCCCCGCGCCGTCCTACCACCGCCACGCCGCCGCGACTCACGACACGACGCCCTA

CCACACGCTCTACGGCGGCCCCTACTCCTCCGCCGGCCGCCAGCAGCACGCCAGCGCCTACCACCACGCC

GCGCAGGTCAGCCCGTTCCACCTGCACCTCGACACCACCCACCCGCACCCGCCGCCGTCCTACTACTCCA

CCATGGACCACAGCAAGGACAGCTACGCCTACGGGCACAGCGTCAAGGAGGTGCACGGCGGCGGCGAGCA

CGCCTTCTTCTCCTCCGACGTCACCACTGACAGGGACCACCACCACCACCAACACCACGCCGGCGCCGGC

GGCAACGGGCAGTGGCAGTTCAAGCAGCTCGGCGGCATGGAGCCCAAGCAGCACAACCCCACGTCGCTCT

TCCCCGGCTGCGGCGGGTACGGCAACAACGCGGCGTACGCCATCGACCTGTCCAGCAAAGAAGAGGACGA

GGAGAAGGAGAGGCGGCAGCAGCAGCAGCACTGCTTCCTGCTGGGCGCCGACCTGAGGCTCGACAAGCCG

TCGTCGGGGCACGGCGACTCCGCCGACCAGAAGCCTCTCCGGCCCTTCTTCGACGAGTGGCCGCACGAGA

AGACCGGGAGCAAGGGGTCGTGGATGGGGCTCGAGGGGGAGACGCAGCTCTCCATCTCCATCGCCAACGA

ACTCCCCATCACCACCACCTCCCGCTACCACCATGGTGAATGA

>TaGRF8-6D

ATGGCCGACGAGAAAGAAGCCGACTCGCTGCAGCCGCCGTCCAAGCAGCCCCGCCTCTCCTCCGCCGACT

CGAACGCCGGTGCGGTGACGATGGCGGTCTCGTCGCCGCTGGGTCTTGGCCTCGGCCTGGGACTCGGCGG

CGATAGCCGCGGCGAGCAGCAGGCCTTCCAAGCACGGGCAGCCGCGGCGGCGGCGAAATCGGCGCTGACG

TTCATGCAGCAGCAGGAGCTGGAGCACCAGGTGCTCATCTACCGCTACTTCGCGGCGGGGGCGCCCGTGC

CGGTGAACCTCGTGCTGCCCATCTGGAAGAGCATCGCCGCCTCCTCCTTCGGCCCGCACCGCTTCCCCTC

CCTGATTGGGCTTGGGAGCCTGTGCTTCGACTACCGGAGCAGCATGGAGCCGGAGCCCGGGAGGTGCCGC

CGCACGGACGGCAAGAAGTGGCGGTGCTCCCGCGACGTGCTGCAGGGGCACAAGTACTGCGAGCGGCACG

TCCACCGGGGCCGCGGCCGTTCAAGAAAGCCTGTGGAAGGAGCCCCCGCAGCCCCGGCGCACAGCGGCAG

CAGCACCACCGCCCCGCCCCGCGCCATCGGCTTCTCCCCCGCCGGCATCCTCCACGCCACCCACAGCGCC

GCCGCGCGCGCCACCTGA

>TaGRF9-6D

ATGGCGATGCCGTATGCCTCTCTTTCCCCGGCAGGCGACCGCCGCTCCTCCCCGGCCGCCACCGCCTCCC

TCCTCCCCTTCTGCCGCTCCTCCCCCTTCTCCGCCGGCGGCGGCAATGGCGGCATGGGGGAGGAGGCGCG

GATGGACGGGAGGTGGATGGCGAGGCCGGTGCCCTTCACGGCGGCGCAGTACGAGGAGCTGGAGCACCAG

GCGCTGATATACAAGTACCTGGTGGCCGGCGTGCCCGTCCCGCCGGATCTCGTGCTCCCCATCCGCCGCG

GCATCGAATCCCTCGCCGCCCGCTTCTACCACAACCCCCTCGCCATCGGGTACGGATCGTACCTAGGCAA

GAAGGTGGATCCGGAGCCGGGCCGGTGCCGGCGCACGGACGGCAAGAAGTGGCGGTGCGCCAAGGAGGCC

GCCTCCGATTCCAAGTATTGCGAGCGCCACATGCACCGCGGCCGCAACCGTTCAAGAAAGCCTGTGGAAA

CGCAGCTCGTCCCGCACACCCAGCCGCCGGCCGCCTCCGCCGTGCCGCCCCTCGCCACCGGCTTCCACAG

CCACTCCCTCTACCCCGCCATCGGCGGCAGCACCAACGGTGGTGGAGGCGGGGGGAACAACGGCATGTCC

ATGCCCAGCACGTTCTCCTCCGCGCTGGGGCCGCCTCAGCAGCACATGGGCAGCAATGCCGCCTCTCCCT

ACGCGGCTCTCGGTGGCGCCGGAACATGCAAAGATTTCAGGTATACCGCATATGGAATAAGATCTTTGGC

AGACGAGCACAGTCAGCTCATGACAGAAGCCATGAATACCTCCGTGGAGAACCCATGGCGCCTGCCGCCG

TCGTCTCAAACGACCTCATTCCCGCTTTCAAGCTACGCTCCTCAGCTTGGAGCAACGAGTGACCTGGGTC

AGAACAACAACCACAACAACAGCAGCAGCAACAGTGCCGTCAAGTCCGAGCGGCAGCAGCCGCTCTCCTT

CCCGGGGTGCGGCGACTTTGGCGGCGGCGGCATGGACTCCGCGAAGCAGGAGAACCAGACGCTGCGGCCG

TTCTTCGACGAGTGGCCGAAGACGAGGGACTCGTGGTCGGACCTGACGGACGACAACTCCAGCCTCGCCT

CCTTCTCGGCCACCCAGCTGTCGATCTCGATACCCATGACGTCCTCCGACTTCTCCGCCGCCAGCTCCCA

GTCGCCCAACGGTATGCTGTTCGCCGGCGAGATGTACTAG

>TaGRF10-6D

ATGATGATGATGGGTGGTCGCGCGGGGGCCGGCGGCGTCGGGGCAGGCGGGGGGCGGTGCCCGTTCACGG

CGACGCAGTGGCAGGAGCTTGAGCACCAGGCACTCATCTACAAGTACATGGCCTCCGGCGTGCCCATCCC

CTCCGACCTCCTCCTCCCGCTCCGCCGCAGCTTCCTCCTCGACTCCGCCCTCGCCACCTCCCCCTCCCTC

GCCTTCCCTCCCCAGGCCGCACTTGGCTGGGGTTGCTTTGGCATGGGGTTCGGCCGGAAGGCGGAGGACC

CGGAGCCGGGGCGGTGCCGGCGGACGGACGGCAAGAAGTGGCGCTGCTCCAAGGAGGCGTACCCGGACTC

CAAGTACTGCGAGAAGCACATGCACCGCGGCAAGAACCGTTCAAGAAAGCCTGTGGAAATGTCCTTGGCC

ACGCCCCCGCCGCCGCCTTCCTCCTCGGCCTCCTCCTCCTCCTCCAACGTCCACTCCGCCGTCAACGTCG

CCACCACCACCTCCTCCCCCGCGCCGTCCTACCACCGCCACGCCGCCGCGACTCACGACACGACGCCCTA

CCACGCGCTCTACGGCGGCCCCTACTCCTCCGCCGGCCGCCAGCAGCACGCTAGCGCCTACCACCACGCC

GCGCAGGTCAGCCCGTTCCACCTGCACCTCGACACCACCCACCCGCACCCGCCGCCGTCCTACTACTCCA

GCATGGACCACAGCAAGGACAGCTACGCCTACGGGCACAGCGTCAAGGAGGTGCACGGCGGCGGCGAGCA

CGCCTTCTTCTCCTCCGACGTCACCACCGACAGGGACCATCACCACCACCACCATCAGCACCAACACCAC

GCTAGCGCCGGCGGCAACGGCCAGTGGCAGTTCAAGCAGCTCGGCGGCATGGAGCCGAAGCAGCATAACC

CAACGTCGCTCTTCCCCGGCTGCGGCGGCTACGGCAACAACGCGGCCTACGCCATCGACCTGTCCAGCAA

AGAAGAGGACGAGGAGAAGGAGAGGCGGCAGCAGCAGCAGCACTGCTTCCTGCTGGGCGCCGACCTGAGG

CTCGACAAGCCGTCGTCGGGGCACGGCGACTCCGCCGACCAGAAGCCTCTCCGGCCCTTCTTCGACGAGT

GGCCGCACGAGAAGACCGGGAGCAAGGGGTCGTGGATGGGGCTCGAGGGGGAGACGCAGCTCTCCATCTC

CATCGCCAACGAACTCCCCATCACCACCACCTCCCGCTACCACCATGGTGAATGA

>TaGRF11-7A

ATGCTGAGCTCGTCGGCGGCGATGGGGATGGGGCTGGGCGGGTACGGCCAGCAGCAGCAGCAGCAGCAGA

TGCAGATGCAGATGCAGATGCAGCGGGGGGCGGGGCCGGTGTTCACGCCGGCGCAGTGGGCCGAGCTGGA

GCAGCAGGCGCTGATTTACAAGTACCTCATGGCGGGCGTGCCCGTGCCGCCCGATCTCCTGCTCCCCATC

CGCCCCCACCCCGCCGGCGCCGGAGCCACCTTCTCCTTCGCCAACCCCGCCGCCTCGCCCTTCTACCACC

ACCACCACCCCTCCATGAGTTACTACGCCTACTATGGCAAGAAGCTCGACCCGGAGCCGTGGCGGTGCCG

CCGCACCGACGGCAAGAAGTGGAGGTGCTCCAAGGAGGCGCACCCCGACTCCAAGTACTGCGAGCGCCAC

ATGCACCGTGGCCGCAACCGTTCAAGAAAGCCTGTGGAATCCAAGTCTGCTTCCCCTGCGCACCAGTCGC

AGCAGCCCCCGTTGTCCGCCGTCACGTCCGCCACCCGCGACGCCGAGCCTCTCCCCTCCCTCCCGGCGGG

GGCTAAGACCCATGGCCTGTCCCTCGGCGGGGCTGGCTCGTCGCAGATGCACGTCGACGCCTCGTCATAC

GGCAACAAATACTCCCTTGGAGCTAAATCTGACGTGGGTGAACTGAGCTTCTTCTCTGGAGCATCAGGAA

ACAACAACAGGGGCTTCACCATCGATTCCCCAACGGACAGCTCGTGGCACTCAATGGGATCCAGCCTGCC

CCCGTACCAACTGTCGAAACCTAGAGATTCCGGCCTCATGCAAGGCGGCTTCTCGTATTCCCACTTTGAG

CCGTCGCAGGAGCTTGGGCAGGTAACCATCGCCTCGCTGTCCCACTCCCAGGAGCAGGACCGCCGCTCCT

TCGGTGGCGGCGGTGGTGGTGGAGGTGGAGGGGCAGGGCTCATGGGAAATGTTAAGCAGGAGAACCAGCC

GCTGAGGCCCTTCTTCGACGAGTGGCCGGGGAGGCGGGACTCGTGGTCGGAGATGGACGACGAGCGCTCC

AACGGCACCTCCTTCTCGACGACCCAGCTCTCGATCTCCATCCCAATGCCTCGATGTGATTGA

>TaGRF12-7A

ATGATGCTGGGAGGGCACGGCGGCGGCGGCGGGAGGTGCCTGTTCACGGCGTCGCAGTGGCGGGAGCTGG

AGCACCAGGCGCTCATCTACAAGTACATGGCCGCCGGCTCGCAGGTGCCCCACGAGCTGGTCCTCCCGCT

CCGCCACCGCGACGCCGCCGCCTTCGCCGGCATCGACACCGCCCCCTCCGTCGCCTGCTACCCTCCTCCG

CAGCCCTCCCTGGGGTGGGGGCTCTACGGGGCGGGGGCGCAGTACGCGCGGAAGCCGGAGGACCCGGAGC

CCGGGCGGTGCCGGCGGACGGACGGCAAGAAGTGGCGCTGCTCCAGGGAGGCGTACGGGGAGTCCAAGTA

CTGCGACAGGCACATGCACCGCGGCAAGAACCGTTCAAGAAAGCCTGTGGAACCGATGAGCTCCTCCTCC

GTCTCCTCCCCGGCCGCCTCCTACCGCCAGACCACCCTCTCCATGTCGCCCCCCACGCCGGCCGACACGC

CCAGCTACGGCCACGGCCACCTCCGCGCAGCTGCTTCTCAGAGCCAGATAAACCCTCTCCAGCTCCACCT

CGACACCCCGTCGCCCCCGCCGTCCTACCACAGGTACGCGCCGGCGCAGCAGTACGGGGGCTCCTTCTTC

CCGAGCAGGCAGCAGGTGCAGGAGGAGGCCGAGGCGGAGGCGAGGCGGCGGCAGCACTTCCTGGCTCTCG

GCGCCGACCTGAGCCTGGACAAGCCGGACGCCACCACCGCGGCGTCCTCGACAACCGAGGAGAAGCCGCT

GCGGCGCTTCTTCGACGAGTGGCCGCGCGACGGGAACGCCGTCGAGGTTCGGCCCTGGAATATGGGCCAC

CGGGACGAGACGCTGCTCTCCATGTCCATCCCCACGACGACGGCCTCGCACCCCGACCTCGCCGCCTACC

GCCACCACAACGATGAATAA

>TaGRF12-7B

ATGATGCTGGGAGGGCACGGCGGCGGCGGCGGCGGCGGGAGGTGCCTGTTCACGGCGTCGCAGTGGCGGG

AGCTGGAGCACCAGGCGCTCATCTACAAGTACATGGCCGCCGGCTCGCAGGTGCCCCACGAGCTGGTCCT

CCCGCTCCGCCACCGCGACGCAGCCTTCGCCGCCATCGACACCGCCCCCTCCCTCGCCTGCTACCCTCCT

CCGCAGCCATCCCTGGGGTGGGGGCTCTACGGGGCGGGATCGCAGTACGCGCGGAAGCCGGAGGACCCGG

AGCCCGGGCGGTGCCGGCGGACGGACGGCAAGAAATGGCGGTGCTCCAGGGAGGCGTATGGGGAGTCCAA

GTACTGCGACAGGCACATGCACCGCGGCAAGAACCGTTCAAGAAAGCCTGTGGAACCAATGAGCTCCGCC

TCCTCCGTCTCCTCCCCGGCCGCCTCGTACCGCCACACCGCCCTCTCCATGTCGCCCCCCACGCCGGCCG

ACACGCCCAGCTACGGCCACGGCCACGGCCACGACCACCTCCGCGCAGCTGCTGGTCAGAGCCAGATAAA

CCCTCTCCAGCTCCACCTCGACACCCCGTCGCCCCCGCCGTCCTACCACAGGTACGCGCCGGCGCAGCAG

TACGGGGGCTCCTTCTTCCCGAGCAGGCAGCAGGTGCAGGAGGAGGAGGCGAGGCGGCGGCAGCACTTCC

TGGCTCTCGGCGCCGACCTGAGCCTGGACAAGCCGGACGCCACCACCGCGGCGTCCTCGACAACCGAGGA

GAAGCCGCTGCGGCGCTTCTTCGACGAGTGGCCGCGCGACGGGAACGCCGTCGAGGGTAGGCCCTGGAAT

ATGGGCCACCGGGACGAGACGCTGCTCTCCATGTCCATCCCCACGACGACGGCCTCGCACCCCGACCTCG

CCGCCGCCTCGCGCTACCACCACCACCACAACGATGAATAA

>TaGRF11-7D

ATGCTGAGCTCGTCGGCGGCGATGGGGATGGGGCTGGGCGGGTACGGCCAGCAGCAGCAGCAGCAGATGC

AGATGCAGATGCAGCGGGGGGCGGAGCCGGTGTTCACGCCGGCGCAGTGGGCCGAGCTGGAGCAGCAGGC

GCTGATTTACAAGTACCTCATGGCGGGCGTGCCCGTGCCGCCCGATCTCCTGCTCCCCATCCGCCCCCAC

CCCGCCGGCGCCGCCGGAACCACCTTCTCCTTCGCCAACCCCGCCGCCTCGCCCTTCTACCACCACCACC

ACCCCTCCATGAGTTACTACGCCTACTATGGCAAGAAGCTCGACCCGGAGCCGTGGCGGTGCCGACGCAC

CGACGGCAAGAAGTGGCGGTGCTCCAAGGAGGCGCACCCCGACTCCAAGTACTGCGAGCGCCACATGCAC

CGTGGCCGCAACCGTTCAAGAAAGCCTGTGGAATCCAAGTCTGCTTCCCCTGCGCACCAGTCGCAGCAGC

CCCAGTTGTCCGCCGTCACGTCCGCGGCCCGCGACGCCGAGCCTCTCCCCTCCCTCCCGGCGGGGGCTAA

AACCCATGGCCTGTCCCTCGGCGGGGCTGGCTCGTCGCAGATGCACGTCGACGCCTCGTCATACGGCGGC

AAATACTCCCTTGGAGCTAAATCTGATGTGGGTGAACTGAGCTTCTTCTCTGGAGCATCAGGAAACAACA

ACAGGGGATTCACCATCGATTCCCCAACGGACAGCTCGTGGCACTCAATGGGATCCAGCCTGACCCCATA

CCAACTGTCGAAACCTAGAGATTCCGGCCTCATGCAGGGCGGCTTCTCGTATTCCCACTTTGAGCCGTCG

CAGGAGCTCGGGCAGGTAACCATCGCCTCGCTGTCCCACTCCCAGGAGCAGGACCGCCGCTCTTTCGGGG

GCGGTGGTGGAGGTGGGGGTGGAGGGGCAGGGCTCATGGGAAATGTTAAGCAGGAGAACCAGCCGCTGAG

GCCCTTCTTCGACGAGTGGCCGGGGAGGCGGGACTCGTGGTCGGAGATGGACGACGAGCGCTCCAACGGC

ACCTCCTTCTCGACGACCCAGCTCTCGATCTCCATCCCAATGCCTCGATGCGATTGA

>TaGRF12-7D

ATGATGCTGGGAGGGCACGGCGGCGGCGGCGGCGGGAGGTGCCTGTTCACGGCGTCGCAGTGGCGGGAGC

TGGAGCACCAGGCGCTCATCTACAAGTACATGGCCGCCGGCTCGCAGGTGCCCCACGAGCTGGTCCTCCC

GCTCCGCCACCGCGACGCTGCCGCCTTCGCCGCCATCGACACCGTCCCCTCCCTCGCCTGCTACCCTCCT

CCGCAGCCATCCCTGGGGTGGGGGCTCTACGGGGCGGGGGCGCAGTACGCGCGGAAGCCGGAGGACCCGG

AGCCGGGGCGGTGCCGGCGGACGGACGGCAAGAAGTGGCGCTGCTCCAGGGAGGCGTACGGGGAGTCCAA

GTACTGCGACAGGCACATGCACCGCGGCAAGAACCGTTCAAGAAAGCCTGTGGAACCGATGTCCTCCTCC

TCCGTCTCCTCCCCGGCCGCCTCCTACCGCCAGACCGCCCTCTCCATGTCGCCCCCCACGCCGGCCGACA

CGCCCACCTACGGCCACGGCCACGGCCACGACCACCTCCGCGCAGCTGCTGGTCAGAGTCAGAGCCAGAT

AAACCCTCTCCAGCTCCACCTCGACACCCCGTCGCCCCCGCCGTCCTACCACAGGTACGCGCCGGCGCAG

CAGTACGGGGGCTCCTTCTTCCAGAACAGGCAGCAGGTGCAGGAGGAGGCGGAGGCGGAGGCGAGGCGGC

GGCAGCACTTCCTGGCTCTCGGCGCCGACCTGAGCCTGGACAAGCCCGACGCCACCACCGCGGCGTCCTC

GACAACCGAGGAGAAGCCGCTGCGGCGCTTCTTCGACGAGTGGCCGCGCGACGGGAACGCCGTCGAGGGT

CGGCCCTGGAATATGGGCCACCGGGACGAGACGCTGCTCTCCATGTCCATCCCCACGACGACGGCCACGC

ACCCCGACCTCGCCGCCGCCTCGCGCTACCACCACCACCACAACGATGAATAA

>AeGRF1

ATCCTCCCTCAAAGCGAGAGCTCCGTATCGCCGGCCTCCCCCTCCGATCCAATCCCTGCC

TTTTGCCCTCTTCTCTTGCGCGGCCCTTTCGCTACCATGGCGGCGGAGGGGGAGGACAAG

AAGGATGCTAATTCCGTTGGAGGCGGCGGCGGCGGCGAGAACACCCTGGAGGCGGCGGAG

GAGGCGATTCTGCAGGCGGTAGGGCAAGAACCCGGTCAAGAATTGGAGGGCGAGGCGGAG

GAGAGCGCAGATCGAGAGGGGAACGGCGACGACACTGGGAAGGAAGATAGTGGGTGTAAA

GATCTGGTCCTGGTAGAGGACCCTGTCCTGGTCGAGGATCCAGAGGAAGCAACTGCAGCA

CTTCAGGAAGAGATGAGAGCGCTTTTGGCGTCTGTCCCTGAAGGTGCTGGGGCATCATTT

ACTGCGATGCAGCTGCAGGAGCTAGAGCAGCAGTCTCGGGTATACCAGTATATGGCTGCC

CGTGTGCCTGTGCCTACCCATCTCGTCTTCCCCATCTGGAAGAGTGTTACCGGTGCATCC

TCTGAAGGCGCGCAGAATTACCCTACATTGATGGGATTGGCAACACTCTGCTTGGACTTC

GGGAAGAGCCCAGAACCAGAACCAGGAAGGTGCCGGCGAACAGATGGAAAAAAGTGGCGA

TGCTGGAGAAAAACAATCCCAAACGAGAAATATTGTGAACGCCATATGCACCGTGGTCGC

AAGCGTCCTGTACAGGTTATTGTTGAGGATGACGAGCCTGATTCCGCATCAGGGTCAAAA

TCGTCATCTGGCAAAGTCACTGAAGGAGGCAAGAAGACTGACAACAAGAGTTCAAGTAGC

AAGAAGCTTGCAGTGGCAGCACCAGCTGCTGTGGAGTTTACATGA

>AeGRF2

ATGGCGGCGCCGTCGCCGCTGGTTCTTGGGCTGGGTCTCGGCGTAGGCGGCAGCTGCAGT

GACAGCGGACGCGGCGACGCGGAGGCCTCTGCGGCGACGCGGCCGTCGGCGCTGACGTTC

ATGCAGCGGCAGGAGCTGGAGCACCAGGTGCTCATCTACCGCTACTTCGCCGCCAACGCT

CCCGTGCCGGTTCACCTCGTCCTCCCCATCTGGAAGAGCGTCGCCGCCTCCTCCTCCGCC

CCGCAGAGGTTCCCATCCCTGGCGGGACTGGGGAGCATGTGCTACGACCACAGGAGCAGC

ATGGAGCCGGAGCCGGACCGGTGCCGGCGCACGGACGGCAAGAAGTGGCGGTGCTCGCGC

GGGGTGGTGCCGGGGCACAAGTACTGCGAGCGCCACGTCCACCGCGGCCGCGGCCGTGCA

AGAAAGCCTGTGGAAGCCGCGGCGGCCACATCAGCCATCCCGATCCGCGCGATGCACGCC

GCCGACGCGCAGGGCGCCACCAGCGCGCACGCGGCGCCGCCGCAGCGCCTCGGCTTCTCC

TCCCCCGCCGGCGTGTACCTGGCCCACGGCACCGCCCGTGCCACCTGA

>AeGRF3

ATGAGCGGCGCGAGGTGGGCGGCGATGAGGCCGGCGACCTTCACGGCGGCGCAGTACCAG

GAGCTGGAGCAGCAGGCGCTCATCTACAAGTACCTCGTCGCCGGCGTGCCCGTCCCGCCG

GATCTCCTCCTCCCCATCCGCCGCGGCTTCGACTCCCTCGCCTCGCGCTTCTACCACCAC

CACGCCCTTGGGTACGGGTCCTACTTCGGGAAGAAGCTGGATCCGGAGCCGGGGCGGTGC

CGGCGGACGGACGGCAAGAAGTGGCGGTGCTCCAAGGAGGCCGCCCAGGACTCCAAGTAC

TGCGAGCGCCACATGCACCGCGGCCGCAACCGTTCAAGAAAGCCTGTGGAAACGCAGCTC

GTCGCCACGCCCCACTCCCACTCCCACTCCCAGCAGCTGCAGCAGCACGCCCCCGCCGCC

ACCGCCGCCGCGTTCCACAGCCACTCGCCGTACCCGGCGATCGCCTCTGGCGGCGGCGGC

GGCGCGGCCGGCTCCTTCGGCCTGGGGTCTGCTCAGCTGCACATGGACAATGCTGCTGCG

CCTTACGCGACCGCTGGTGCGGCCGGAAACAAGGATTTCAGGTATTCTGCCTATGGGTTT

AGGACTTCGGCGCTGGAGGAGCACAACCAGTTCATCAGCGCGGCCATGGACACCGCCATG

GACAACTACTCATGGCGCCTGATGCCGTCTCAGCCCTCATCCTTCTCACTCGCCAGCTAC

CCCATGCTGGGCACCCTGGGCGACCTGGACCAGAGCGCGATCTGCTCGCTGGCCAAGACG

GAGAGGGAGCCGCTGTCCTTCGGCGGCGGCGGCGGCTTCGAGGACGACGAGTCGGCGGTG

AAGCAGGAGAACCAGACGCTGCGGCCCTTCTTCGACGAGTGGCCCAAGGACAGGGACTCG

TGGCCGGAGCTGCAGGACCATGACTCCAACCACAACAGCAACGCCTTCTCGGCCACCAAG

CTGTCCATCTCCATCCCGGTGACCAGCTCCGACTTCTCCACCACCGCCGGCTCCCGCTCG

CCCCACGGTATATACTCCCGGTGA

>AeGRF4

ATGGAGCTCGGGCAGGTGCTGGGCTACACGCCGCCGGCGACCAAGGACGCGAGATCCGGC

GGCGGCTTCGCCCAGGCCGCCGCTTGCCCCTACCCCTACCCCTCCCCCTTCCTCGACGAG

CAGAAGATGCTCAGCTTCTCCAAGGCCGCCGCCCCTCCATCGTCAGGTATGGATTTTGGC

AGGTCCAATGAGCAGAGGCTGCTGCTGGCCAGGAGCAAGATGCCCTTCACTCCTTCACAG

TGGATGGAGCTGGAGCACCAGGCCCTCATATACAAGTATCTCAATGCAAAGGCCCCCATA

CCTTCCAGCCTGCTCATCTCCATCAGCAAAAGCTTCAGACCCTCCTCCGATAGAATGCCC

TGGAGGCCTGTCTACCAAGGGTTCACCAATGCAGATTCTGACCCGGAACCTGGAAGATGC

CGTCGAACAGACGGCAAGAAATGGCGGTGCTCAAAGGAGGCGATGGCCGAGCACAAGTAC

TGTGAGCGGCACATCAATAGGAACCGCCATCGTTCAAGAAAGCCTGTGGAAAACCAAACA

AGGAAGAACGCCAAAGAGACGCCTGCTGCTGGCTCGTTATCGGCCGCTGTCTCACAGGGT

GGCTGTAAGAAAGCAAAAGCTGGTGATGAACTGAAGCCAGGGAGCGTCAGCTATTGGACA

GATAATTTAAACAGGGCAATGGTGAGCAAAGCCAGGGGAAACAACCCTGAAGAAGGCAAC

AGTGCTCCACTCCTGAATTCTACTAATCAACAACACACATTGTCCTTGTTCTCTCAACTG

AAGCAACAGAGCAAACCAGATAAGTTCAGCCCTGCAGTCGATAGTGAATCGATCTCCTCA

AATACAGTATTGAAGCCCTGGGAAAGAAGCAACCAGCAGAGCAGCAAGGACGTTTCTTCG

ACGACGCTCCATGATCGCGGGTGCCTTCAATCAGTCCTTCAAGATTTCAGCATGCATAAG

AATGACAAGATCGAGGCTCAGAAAAACAATGCTTCAGTGCCATCAACTTTCTATTCACCT

ACAGAAGGTCGACACATCAGCTGCCTTGCATCTAACATGATGCAAGTGCAGGAGGATTGC

ATCTCAAGCTCTTGGGAGATACCTCAAGGTGGGCCATTAGGTGAAATCCTAACAAACTCC

AAGAACACTGATGACTTGACCAATAAGTGTGAATCAAGATCATATGGTTGGTTACTGAGT

CTTGATGAACATGAAATGTGA

>AeGRF5

ATGGACCTGGGCGGGGTGCTGATGGCGGCCGCGGACGCGGGGGTGGGCGGCGGGGACCTC

GGCATGCTCGGATCTAGGCTGCTCAAGCACGGGAGGGGCAATGAGGCCGACGAGCACGGC

TGGGGCGGCGGCAGGCCGGCGTCCAAGCAGGCCCGGGTCGCCGGGGACAGCGACGCGGTG

TCCGAGGCCGTCAAGGCGGCCGCGCCCTACCTGCTCGGCACCTGCAGCCCCGGGCACGGC

CGGGAGAAGATGCTCAGCTTCTCCTCCTCGCAGCCGGCCTCCTGCCCCTCCGCCGCTCAG

GCCGCGCTGCCGCTCTACTACGGCACGCCCGCTTCTTGCTCAGGGTTGAGCTCAGTGAGG

GGGCCCTTCACGCCGTCGCAGTGGATGGAGCTGGAGCACCAGGCCCTGATCTACAAGTAC

CTGGCGGCCAACATCGCCGTGCCTCACAACCTCGTCGTCCCCATCCGCCGGAGCGTCACC

TCGCTCTACCCGTCCGCCTACTTTGGCTCCTCCACATTGGGGTGGGGGCCTTTCCAGCTG

GGCTACTCCGGGAGCGCGGACCTGGAGCCCGGGCGGTGCCGCCGGACGGACGGCAAGAAG

TGGCGGTGCTCCAGGGACGCCGTCGCCGACCAGAAGTACTGCGAGCGGCATATGAACCGG

GGACGCCATCGTTCAAGAAAGCATGTGGAAGGCCAGCCTGGCCATGCCGCGAAAGCGATG

CCTGCGACGGCCGCTGCTGCCGCCCAGCCCGGTGCTCTCGCCACCGGGGGCGGCGGCGGA

GCTCCCGCCGGCGCCGCCATCTGCCACGAGCAGCAACCGTTGAAGAACTACGCCGCCAGC

ACCATTGATCCTTGTTCACTGCAATATAACAGGGAAATGGTGAGCAAGCAGCAACACGAG

TGCGAGCAAGTGCAGGACTCCGACACCCTCTCGATGCTGACCTCCATGAGCGCGAGGAAC

ACCAATGCAGGCAGCATGTTCCCGTTCTCAAAGGAACATCATAACCACAATCCTTTCGAG

GTGACGAGCTCGAGGCCGGACTACGGGCTGGTTTCATCCGACTCGCTGATGAGCTCCCCT

CACAGCTCCCTGGAGAACGTCAACCTGCTCACCTCGCAGCGAGCTCTCTCGAGCGAGCAG

CAGAGCTCGCTCTCCCTGCAGCACTTCGCGGACTGGCCGAGGACGCCCTCGCAGCAGGGG

CAGGGAGGAGGTCTCTCATGGCCGGACGCCGAGGACATGCAAGCTCATCAGAGGACCCAG

CTCTCGGTGTCCGCCGTTCCAATGGCGTCCTCTGACCTGTCGTCGGCCTCCACGTCCCCG

ATCCACGAGAAGCTCATGCTGTCGCCCCTCAAGCTGAGCCGCGAGTACAGCCCCATCGGC

CTCAGCGTCGCGGCCACGGCGGCGGTGGCGAAGGACGAGGGGGAGGCGAACTGGATGCCC

ATGTTCCGCGACTCGTCCATGGGCGGGCCGCTGGGGGAGGCTCTGAACAAGAACAATGGC

GGCAACATGGAGGCCAAGAGCTACCTGTCGGCGTCGCTGAACCTGATGACGGACGCCTGG

GACTCGAGCCCGCTGGAGTCGTCGCCGGTGGGGGTCCTGCAGAGGACCGCCTTCGGATCG

GTGTCCAGCAGCACCGGCAGCAGCCCCAGGCAGGAGTACCACGGCGTGTATGATGGTAAC

CCGCGGGATGATCTCGGCTCCATCGTCGTGAATCACCCCAGCATCCGCCTGATGTGA

>AeGRF6

ATGGCCGACGAGAAAGAAGCCGACTCGCTGCAGCCGCCGTCCAAGCAGCCCCGCCTCTCC

TCCGCCGACTCGAACGCCGGTGCGGTGACGATGGCGGTCTCGTCGCCGCTGGGTCTTGGC

CTCGGCCTGGGACTCGGCGGCGATAGCCGCGGCGAGCAGCAGGCCTTCCAAGCACGGGCA

GCCGCGGCGGCGGCGAAATCGGCGCTGACGTTCATGCAGCAGCAGGAGCTGGAGCACCAG

GTGCTCATCTACCGCTACTTCGCGGCGGGGGCGCCCGTGCCGGTGCACCTCGTGCTGCCC

ATCTGGAAGAGCATCGCCGCCTCCTCCTTCGGCCCGCACCGCTTCCCCTCCCTGATTGGG

CTTGGGAGCCTGTGCTTCGACTACCGGAGCAGCATGGAGCCGGAGCCCGGGAGGTGCCGC

CGCACGGACGGCAAGAAGTGGCGGTGCTCCCGCGACGTGCTGCAGGGGCACAAGTACTGC

GAGCGGCACGTCCACCGGGGCCGCGGCCGTTCAAGAAAGCCTGTGGAAGGAGCCCCCGCA

GCCCCGGCGCACAGCGGCAGCAGCACCACCGCCCCGCCCCGCGCCATCGGCTTCTCCCCC

GCCGGCATCCTCCACGCCACCCACAGCGCCGCCGCGCGCGCCACCTGA

>AeGRF7

ATGTTCGTGTGCAGCGGCGGCGGCAATGGCGGCATGGGGGAGGAGGCGCGGATGGACGGG

AGGTGGATGGCGAGGCCGGTGCCCTTCACGGCGGCGCAGTACGAGGAGCTGGAGCACCAG

GCGCTGATATACAAGTACCTGGTGGCCGGCGTGCCCGTCCCGCCGGATCTCGTGCTCCCC

ATCCGCCGCGGCATCGAATCCCTCGCCGCCCGCTTCTACCACAACCCCCTCGCCATCGGG

TACGGATCGTACCTAGGCAAGAAGGTGGATCCGGAGCCGGGCCGGTGCCGGCGCACGGAC

GGCAAGAAGTGGCGGTGCGCCAAGGAGGCCGCCTCCGATTCCAAGTATTGCGAGCGCCAC

ATGCACCGCGGCCGCAACCGTTCAAGAAAGCCTGTGGAAACGCAGCTCGTCCCGCACACC

CAGCCGCCGGCCGCCTCCGCCGTGCCGCCCCTCGCCACCGGCTTCCACAGCCACTCCCTC

TACCCCGCCATCGGCGGCAGCACCAACGGTGGTGGAGGCGGGGGGAACAACGGCATGTCC

ATGCCCAGCACGTTCTCCTCCGCGCTGGGGCCGCCTCAGCAGCACATGGGCAGCAATGCC

GCCTCTCCCTACGCGGCTCTCGGTGGCGCCGGAACATGCAAAGATTTCAGGTATACCGCA

TATGGAATAAGATCTTTGGCAGACGAGCACAGTCAGCTCATGACAGAAGCCATGAATACC

TCCGTGGAGAACCCATGGCGCCTGCCGCCGTCGTCTCAAACGACCTCATTCCCGCTTTCA

AGCTACGCTCCTCAGCTTGGAGCAACGAGTGACCTGGGTCAGAACAACAACCACAACAAC

AGCAGCAGCAACAGTGCCGTCAAGTCCGAGCGGCAGCAGCCGCTCTCCTTCCCGGGGTGC

GGCGACTTTGGCGGCGGCGGCATGGACTCCGCGAAGCAGGAGAACCAGACGCTGCGGCCG

TTCTTCGACGAGTGGCCGAAGACGAGGGACTCGTGGTCGGACCTGACGGACGACAACTCC

AGCCTCGCCTCCTTCTCGGCCACCCAGCTGTCGATCTCGATACCCATGACGTCCTCCGAC

TTCTCCGCCGCCAGCTCCCAGTCGCCCAACGGTATGCTGTTCGCCGGCGAGATGTACTAG

>AeGRF8

ATGATGATGATGGGTGGTCGCGCGGGGGCCGGCGGCGTCGGGGCAGGCGGGGGGCGGTGC

CCGTTCACGGCGACGCAGTGGCAGGAGCTTGAGCACCAGGCACTCATCTACAAGTACATG

GCCTCCGGCGTGCCCATCCCCTCCGACCTCCTCCTCCCGCTCCGCCGCAGCTTCCTCCTC

GACTCCGCCCTCGCCACCTCCCCCTCCCTCGCCTTCCCTCCCCAGGCCGCACTTGGCTGG

GGTTGCTTTGGCATGGGGTTCGGCCGGAAGGCGGAGGACCCGGAGCCGGGGCGGTGCCGG

CGGACGGACGGCAAGAAGTGGCGCTGCTCCAAGGAGGCGTACCCGGACTCCAAGTACTGC

GAGAAGCACATGCACCGCGGCAAGAACCGTTCAAGAAAGCCTGTGGAAATGTCCTTGGCC

ACGCCCCCGCCGCCGCCTTCCTCCTCGGCCTCCTCCTCCTCCTCCAACGTCCACTCCGCC

GTCAACGTCGCCACCACCACCTCCTCCCCCGCACCGTCCTACCACCGCCACGCCGCCGCG

ACTCACGACACGACGCCCTACCACGCGCTCTACGGCGGCCCCTACTCCTCCGCCGGCCGC

CAGCAGCACGCTAGCGCCTACCACCACGCCGCGCAGGTCAGCCCGTTCCACCTGCACCTC

GACACCACCCACCCGCACCCGCCGCCGTCCTACTACTCCAGCATGGACCACAGCAAGGAC

AGCTACGCCTACGGGCACAGCGTCAAGGAGGTGCACGGCGGCGGCGAGCACGCCTTCTTC

TCCTCCGACGTCACCACCGACAGGGACCATCACCACCACCACCATCAGCACCAACACCAC

GCTAGCGCCGGCGGCAACGGCCAGTGGCAGTTCAAGCAGCTCGGCGGCATGGAGCCGAAG

CAGCATAACCCAACGTCGCTCTTCCCCGGCTGCGGCGGCTACGGCAACAACGCGGCCTAC

GCCATCGACCTGTCCAGCAAAGAAGAGGACGAGGAGAAGGAGAGGCGGCAGCAGCAGCAG

CACTGCTTCCTGCTGGGCGCCGACCTGAGGCTCGACAAGCCGTCGTCGGGGCACGGCGAC

TCCGCCGACCAGAAGCCTCTCCGGCCCTTCTTCGACGAGTGGCCGCACGAGAAGACCGGG

AGCAAGGGGTCGTGGATGGGGCTCGAGGGGGAGACGCAGCTCTCCATCTCCATCGCCAAC

GAACTCCCCATCACCACCACCTCCCGCTACCACCATGGTGAATGA

>AeGRF9

ATGCTGAGCTCGTCGGCGGCGATGGGGATGGGGCTGGGCGGGTACGGCCAGCAGCAGCAG

ATGCAGATGCAGATGCAGCGGGGGGCGGGGCCGGTGTTCACGCCGGCGCAGTGGGCCGAG

CTGGAGCAGCAGGCGCTGATTTACAAGTACCTCATGGCGGGCGTGCCCGTGCCGCCCGAT

CTCCTGCTCCCCATCCGCCCCCACCCCGCCGGCGCCGCCGGAACCACCTTCTCCTTCGCC

AACCCCGCCGCCTCGCCCTTCTACCACCACCACCACCCCTCCATGAGTTACTACGCCTAC

TATGGCAAGAAGCTCGACCCGGAGCCATGGCGGTGCCGGCGCACCGACGGCAAGAAGTGG

CGGTGCTCCAAGGAGGCGCACCCCGACTCCAAGTACTGCGAGCGCCACATGCACCGTGGC

CGCAACCGTTCAAGAAAGCCTGTGGAATCCAAGTCTGCTTCCCCTGCGCACCAGTCGCAG

CAGCCCCAGTTGTCCGCCGTCACGTCCGCGGCCCGCGACGCCGAGCCTCTCCCCTCCCTC

CCGGCGGGGGCTAAAACCCATGGCCTGTCCCTCGGCGGGGCTGGCTCGTCGCAGATGCAC

GTCGACGCCTCGTCATACGGCGGCAAATACTCCCTTGGAGCTAAATCTGATGTGGGTGAA

CTGAGCTTCTTCTCTGGAGCATCAGGAAACAACAACAGGGGATTCACCATCGATTCCCCA

ACGGACAGCTCGTGGCACTCAATGGGATCCAGCCTGACCCCATACCAACTGTCGAAACCT

AGAGATTCCGGCCTCATGCAGGGCGGCTTCTCGTATTCCCACTTTGAGCCGTCGCAGGAG

CTCGGGCAGGAGAACCAGCCGCTGAGGCCCTTCTTCGACGAGTGGCCGGGGAGGCGGGAC

TCGTGGTCGGAGATGGACGACGAGCGCTCCAACGGCACCTCCTTCTCGACGACCCAGCTC

TCGATCTCCATCCCAATGCCTCGATGCGATTGA

>AeGRF10

ATGATGCTGGGAGGGCACGGCGGCGGCGGCGGCGGGAGGTGCCTGTTCACGGCGTCGCAG

TGGCGGGAGCTGGAGCACCAGGCGCTCATCTACAAGTACATGGCCGCCGGCTCGCAGGTG

CCCCACGAGCTGGTCCTCCCGCTCCGCCACCGCGACGCTGCCGCCTTCGCCGCCATCGAC

ACCGTCCCCTCCCTCGCCTGCTACCCTCCTCCGCAGCCATCCCTGGGGTGGGGGCTCTAC

GGGGCGGGGGCGCAGTACGCGCGGAAGCCGGAGGACCCGGAGCCGGGGCGGTGCCGGCGG

ACGGACGGCAAGAAGTGGCGCTGCTCCAGGGAGGCGTACGGGGAGTCCAAGTACTGCGAC

AGGCACATGCACCGCGGCAAGAACCGTTCAAGAAAGCCTGTGGAACCGATGTCCTCCTCC

TCCGTCTCCTCCCCGGCCGCCTCCTACCGCCAGACCGCCCTCTCCATGTCGCCCCCCACG

CCGGCCGACACGCCCACCTACGGCCACGGCCACGGCCACGACCACCTCCGCGCAGCTGCT

GGTCAGAGTCAGAGCCAGATAAACCCTCTCCAGCTCCACCTCGACACCCCGTCGCCCCCG

CCGTCCTACCACAGGTACGCGCCGGCGCAGCAGTACGGGGGCTCCTTCTTCCAGAACAGG

CAGCAGGTGCAGGAGGAGGCGGAGGCGGAGGCGAGGCGGCGGCAGCACTTCCTGGCTCTC

GGCGCCGACCTGAGCCTGGACAAGCCCGACGCCACCACCGCGGCGTCCTCGACAACCGAG

GAGAAGCCGCTGCGGCGCTTCTTCGACGAGTGGCCGCGCGACGGGAACGCCGTCGAGGGT

CGGCCCTGGAATATGGGCCACCGGGACGAGACGCTGCTCTCCATGTCCATCCCCACGACG

ACGGCCACGCACCCCGACCTCGCCGCCGCCTCGCGCTACCACCACCACCACAACGGTACG

>TdGRF1

CTCAAAGCGAAAGCTCCGTATCGCCGGACTCCCCCCCGATCCAATCCCAAAGATACCTTT

TGCCCTCTTCTCTTGCGCGGCCCTTTCGCTACCATGGCGGCGGAAGGGGAGGACAAGAAG

GATGCTAATTCCGTTGGAGGCGGCGGCGGCGGCGGCGAGAACACCCTGGAGGCGGCGGAG

GAGGCGATTCTGCAGGCGGTAGGGCAAGAACCCGGTCAAGAATTGGAGGGCGAGGTGGAG

GAGAGCGCAGATCGAGAGGGGAACGGCGACGACGCTGGGAAGGAAGATAGTGGGTGTAAA

GATCTGGTCCTGGTAGAGGACCCTGTCCTGGTCGAGGATCCAGAGGAAGCGGTAGCAACT

GCAGCACTTCAGGAAGAAATGAGAGCGCTTTTCGCGTCTGTCCCTGAAGGTGCTGGGGCA

TCATTTACTGCGATGCAGCTGCAGGAGCTAGAGCAGCAGTCTCGGGTATACCAGTATATG

GCTGCCCGTGTGCCTGTGCCTACCCATCTCGTCTTCCCCATCTGGAAGAGTGTTACCGGT

GCATCCTCTGAAGGCGCGCAGAATTACCCTACATTGATGGGATTGGCAACACTCTGCTTG

GACTTCGGGAAGAGCCCAGAACCAGAACCAGGAAGGTGCCGGCGAACAGATGGAAAAAAG

TGGCGGTGCTGGAGAAAAACAATCCCAAACGAGAAATATTGTGAACGCCATATGCATCGT

GGTCGCAAGCGTCCTGTACAGGTTATTGTTGAGGATGACGAGCCTGATTCCGCATCAGGG

TCAAAATCGTCATCTGGCAAAGTCACTGAAGGAGGCAAGAAGACTGACGACAAGAGTTCA

AGTAGCAAGAAGCTTGCAGTGGCAGCACCAGCTGCTGTGGAGTTTACATGATTGATGATG

CAGCATTTGGAAGGTGCAAGAAGAGCGTAACCGCCATGGCAATTAGAGTTCCGTTATTGT

AATCCTCAAAAGACTTTAGTGTTGTCTAGCTATAACCTCATTAAGCAAGCAAAATGTCTT

GTCGGAAGAAGCCACAAAAACCCCTGTTTAGCTGTCACTGAATCTTTCAGTTTAGGTGTA

TAGTTTGAATTAGCTTGGTCGTGCTTTCGGCCTTTGGGCAGGTGATGCGGCATAATTGGA

TAAGTCTTCCCTCACTGATAAGTCGCATTGTGACTCAAGAAACTGGTGGATGAATCTGCA

GAGAGTAGATACTATTTGTTGTGTGCTTTTATTGGCCTTCACAATTGTGTTGTTTGTCAG

TTGCATAACAATTGGAAGATCCATACAATTTTAGACCCTATGGAGATTCAGTTATGAGAA

TACATATCTTTAGTTTCAACATGCTATTTGTTAATGTAAGTTCTAACAACCTGAGGTGCT

CTGATATGATTTGAATGCATGTGTGATGTAATGATGTGCTAATGGAT

>TdGRF2

ATGACCGAGCGAAGGCAGGAGCACTCGCCGCCGTCCAAGATCCCCCGCCTCTCCGGCGCC

GACGACGATGACGGTAGGGAAGGGCGCAGGGACGGTGACCATGGCGGCGCCGTCGCCGCT

GGTTCTTGGGCTGGGTCTCGGCGTAGGCGGCAGCAGCAGTGACAGCGGACGCGGCGACGC

GGAGGCGTCTGCGGCGACGCGGCCGTCGGCGCTGACGTTCATGCAGCGGCAGGAGCTGGA

GCACCAGGTGCTCATCTACCGCTACTTCGCCGCCAACGCTCCCGTGCCCGTGCACCTCGT

GCTCCCCATCTGGAAGAGCGTCGCCGCTTCCTCCTCCGCCCCGCAGAGGTTTCCATCCCT

GGCGGGGCTGGGGAGCATGTGCTACGACCACAGGAGCAGCATGGAGCCGGAGCCGGACCG

GTGCCGGCGCACGGACGGCAAGAAGTGGCGGTGCTCGCGCGGCGTGGTGCCGGGGCACAA

GTACTGCGAGCGCCACGTCCACCGCGGCCGCGGCCGTGCAAGAAAGCCTGTGGAAGCCGC

GGCGGCCACATCAGCCGTCCCGATCCGCGCGATGCACGCCGCCGACGCGCAGGGCGCCAC

CAGTGCGCACGCGGCGCCACCGCAGCGCCTCGGCTTCTCCTCCCCCGCCGGCGTCTACCT

GGCGCACGGCACCGCCCGTGCCACCTGA

>TdGRF3

AGAGAGAGAGCGGCATATATGGCGATGCCCTTTGCCTCCCTGTCGCCGGCAGCCGACCAC

CACCGCTCCTCCCCCATCTTCCCCTTCTGCCGCTCCTCCCCTCTCTACTCGCAGCAGCAG

CAGCAGCAGCAGCACGCGATGAGCGGCGCGAGGTGGGCGGCGGCGAGGCCGGCGACCTTC

ACGGCGGCGCAGTACGAGGAGCTGGAGCAGCAGGCGCTCATCTACAAGTACCTCGTCGCC

GGCGTGCCCGTCCCGCCGGATCTCCTCCTCCCCATCCGCCGGGGCTTCGACTCCCTCGCC

TCGCGCTTCTACCACCACCACGCCCTTGGGTACGGGTCCTACTTCGGGAAGAAGCTGGAT

CCGGAGCCGGGGCGGTGCCGGCGGACGGACGGCAAGAAGTGGCGGTGCTCCAAGGAGGCC

GCCCAGGACTCCAAGTACTGCGAGCGCCACATGCACCGCGGCCGCAACCGTTCAAGAAAG

CCTGTGGAAACGCAGCTCGTCTCCCACTCCCAGCAGCTGCAGCAGCAGGCCCCCGCCGCC

GCGTTCCACGGCCACTCGCCGTACCCGGCGATCGCCACTGGCGCCGGCGCGCCCGGCTCC

TTCGCCCTGGGGTCTACTGCTCAGCTGCACATGGATAATGCTGCTGCGCCTTACGCGACC

GCTGGCGCCGCCGGGAACAAAGATTTCAGGAGCTCCTGCTTTAAAAATGTGCAGCAACAA

TTGCTCGTATAGTAGTATTCTGCCTATGGGTTTAGGACTTCGGCGATGGAGGACCACAAC

CAGTTCATCAGTGCGGCCATGGACACCGCCATGGACAACTACTCATGGCGCCTGCTGCCG

GCCCAGAACTCGTCCTTCTCACTCTCGAGCTACCCCATGCTGAGCACCCTGAGCGACCTG

GACCAGAGCGCGATCTGCTCGCTGGCCAAGACGGAGAGGGAGCCGCTGTCCTTCTTCGGC

GTGGGCGGCGGCTTCGACGACGACGAGTCGGCGGTGAAGCAGGAGAACCAGACGCTGCGG

CCCTTCTTCGACGAGTGGCCCAAGGACAGGGACTCGTGGCCGGAGCTGCAGGACCATGAC

TCCAACCACAACAATGAGGCCTTCTCGGCCACCAAGCTGTCCATCTCCATCCCGGTGACC

AGCTCCGATTTCTCCACCACCGCCGGCTCCCGCTCGCCCCACGGTATATACTCCCGGTGA

AGGTGAA

>TdGRF4

AACAACTTTGGGTCCCTCTCCTCCTTCCCCCTCAAAGCGAGAGCTCCGTATCGCCGGCCT

CCCCCTCCGATCCAATCCCAAAGATACCTTTTGCCCTCTTCTCTTGCGCGGCCCTTTCGC

TACCATGGCGGCGGAGGGGGAGGACAAGAAGGATGCTAATTCCGTTGGAGGCGGCGGCGG

CGAGAACACCCTGGAGTCGGCGGAGGAGGCGATTCTGCAGGCGGTAGGGCAAGAGCCCGG

TCAAGAATTGGAGGGCGAGGCGGAGGAGAGCGCAGATCGAGAGGGGAACGGCGACGACGC

TGGGAAGGAAGATAGTGGGTGTAAAGATCTGGTCCTGGTAGAGGACCCTGTCCTGCTCGA

GGATCCAGAGGAAGCGGTAGCAACTGCAGCACTTCAGGAAGAAATGAGAGCGCTTTTGGC

GTCTGTCCCTGAAGGTGCCGGGGCATCATTTACTGCGATGCAGCTGCAGGAGCTAGAGCA

GCAGTCTCGGGTATACCAGTATATGGCTGCCCGTGTGCCTGTGCCTACCCATCTCGTCTT

CCCCATCTGGAAGAGTGTTACCGGTGCATCCTCTGAAGGCGCGCAGAATTACCCTACATT

GATGGGATTGGCAACACTCTGCTTGGACTTTGGGAAGAGCCCAGAACCAGAACCAGGAAG

GTGCCGGCGAACAGATGGAAAAAAGTGGCGGTGCTGGAGAAAAACAATCCCAAACGAGAA

ATATTGTGAACGCCATATGCACCGTGGTCGCAAGCGTCCTGTACAGGTTATTGTTGAGGA

TGACGAGCCTGATTCCGCATCAGGGTCAAAATCATCATCTGGCAAAGTCACTGAAGGAGG

CAAGAAGACTGACGACAAGAGTTCAAGTAGCAAGAAGCTTGCAGTGGCAGCACCAGCTGC

TGTGGAGTTTACATGATTGATGATGTAGCATTTGGAAGCTGCAAGAAGAGCGTAACGGCC

ATGGCAATTAGACTACCGTTATTGTAATCCTCAAAAGACTTTAGTGTTGTCTAGCTATAA

CCTCATTAAGCAAGCAAAATGTCTTGTCGGAAGAAGCCACAAAAACCCCTGTTTAGCTGT

CACTGAATTTTTCAGTTTAGGTGTATAGTTTGAATTAGCTTGGTCGTGCCTTCGGCCTTT

AGGCAGGTGATGCGGCATAATTGGATAAGTCTTCCCTCATTGATAAGTCGCATTGTGACT

CAAGAAACTGGTGGATGAATCTGCAGAGAGTAGATACTATTTGGTGTGTGCTTTTATTGG

CCTTCACAATTGTGTTGTTTGTCAGTTGCATAACAATTGGAAGATCAATATAATTTTAGA

CCCTATGGAGATTCAGTTACGAGAC

>TdGRF5

ACTCCAGAGATGACCGAGCGAAGGCAGGAACACTCGCCGCCGTCCAAGCTCCCCCGCCTC

TCCGGCCCCGACGCCGACGACAACGACGGGACGGTGACCATGGCGGCGCCGTCGCCGCTG

GTTCTTGGGCTGGGTCTCGGCGTAGGCGGCAGCAGCAGTGACAGTGGACGAGGCGACGCG

GAGGCATCTGCGGCGACGCGGCCATCGGCGCTGACGTTCATGCAGCGGCAGGAGCTGGAG

CACCAGGTGCTCATCTACCGCTACTTCGCCGCCAACGCTCCCGTGCCCGTGCACCTCGTC

CTCCCCATCTGGAAGAGCGTCGCCGCCTCCTCCTCCGCCCCGCAGAGGTTCCCATCCCTG

GCGGGACTGGGGAGCATGTGCTACGACCACAGGAGCAGCATGGAGCCGGAGCCGGACCGG

TGCCGGCGCACGGACGGCAAAAAGTGGCGGTGCTCGCGCGGCGTGATGCCGGGGCACAAG

TACTGCGAGCGCCACGTCCACCGCGGCCGCGGCCGTGCAAGAAAGCCTGTGGAAGCCGCG

CCGGCCACATCAGCGGGAAATCTCCGAGGATTATGCTGTACTGGTGCTGGTAATTTTTAT

TTTCGCCTTGACCACG

>TdGRF6

GAGGAGAGAGAGCATATATGGCGATGCCCTTTGCCTCCCTGTCGCCGGCAGCCGACCACC

ACCGCTCCTCCCCCATCTTCCCCTTCTGCCGCTCCTCCCCTCTCTACTCGGGAGGAGACG

GCGCATCAGCAGCAGCAGCAGCACACGATGAGCGGCGCGAGGTGGGCTGCGAGGCCGGCG

ACCTTCACGGCGGCGCAGTACGAGGAGCTGGAGCAGCAGGCGCTCATCTACAAGTACCTC

GTCGCCGGCGTCCCCGTCCCGCCGGATCTCCTCCTCCCCATCCGCCGCGGCTTCGACTCC

CTCGCCTCGCGCTTCTACCACCACCACGCCTTCGGGTACGGTTCCTACTTCGGGAAGAAG

CTGGATCCGGAGCCGGGGCGGTGCCGGCGGACGGACGGCAAGAAGTGGCGGTGCTCCAAG

GAGGCCGCCCAGGACTCCAAGTACTGCGAGCGCCACATGCACCGCGGCCGCAACCGTTCA

AGAAAGCCTGTGGAAACGCAGCTCGTCGCCACGCCCCACCACTCCCACTCCCAGCAGCTG

CAGCAGCACGCCCCCGCCGCCAGCGCCGCCGCGTTCCACAGCCACTCGCCGTATCCGGCG

ATCGCCACTGGCGGCGGCGGCTCCTTCGCCGTGGGATCTGCTCAGCTGCACATGGACAAT

GCTGCTTCGCCTTACGCGACCGCTGGTGCCGCCGGAAACAAAGATTTCAGCAACAATTGC

CATGTCCCTTTTGTTTTGTCTAGTAGCCAAGAAACGTGAAGCCTACCGTGTATTCTGCCT

ATGGGTTTAGGACTTCGGCGATGGAGGAGCACAACCAGTTCATCTCTGCGGCCATGGAGA

CCGCCATGGAGAACTACTCATGCCGCCTGATGCCGGCCCAGAACTCATCCTTCTCACTCG

CCAGCTACCCCATGCTGGGCACCCTGGGCGACCTTGACCAGAGCGCGATCTGCTCGCTGG

CCAAGACGGAGAGGGAGCCTCTGTCCTTCTTCGGCGGCGGCGGCGGCTCCGACGACGACG

ACTCGGCGGTGAAGCAGGAGAACCAGACGCTGCGGCCCTTCTTCGACGAGTGGCCCAAGG

ACAGGGACTCGTGGCCGGAGCTGCAGGACCACGATGCCAACAACAGCAGCAACGCCTTCT

CGGCCACCAAGCTGTCCATCTCCATCCCGGTGACCAGCTCCGACTTCTCCACCACCGCCG

GCTCCCGCTCGCCCAACGGTATATACTCCCGGTGAAGGTGAA

>TdGRF7

CACGCTCTCAGATCGAGGTTGTTGCATTAGATCAAAGATTCAAATCTGTGCTTCTTTCTC

ATCTGGTATTGGTTTTCTGATCTTGGCAGCCACACCCTGGCTCAGATTTCCTCCCGCGTG

TTGCTTGCTTGCCCCTGTCAAGAAAGTACACTTTATTTTCTTGGATTTGCCTTGTTGTCT

TGCACTGCCTTGTTGCTCATCTGCTTGTTGTATTATTATCATGGGTGGCAGGGTTGAGCT

CGGTGAGCTTGAGCGCCAGCATCCAGGGCGCCATGGCCAGGGTGAGGGGGCCCTTCACGC

CGTCGCAGTGGATGGAGCTGGAGCACCAGGCCCTGATCTACAAGTACCTGGCGGCCAACA

TCGCCGTGCCGCACAGCCTCCTCGTCCCCATCCGCCGGAGCGTCACCTCGCTCTACCCGT

CCGCCTACTTTGGCTCCTCCACATTGGGGTGGGGGCCTTTCCAGCTGGGCTACTCCGGGA

GCGCGGACCTGGAGCCCGGGCGGTGCCGCCGGACGGACGGCAAGAAGTGGCGGTGCTCCA

GGGACGCCGTCGCTGACCAGAAGTACTGCGAGCGGCATATGAACCGGGGACGCCATCGTT

CAAGAAAGCATGTGGAAGGCCAGCCTGGCCATGCCGCGAAAGCGATGCCTGCGACGGTGG

CGGCTGCTGCCGCCCAGCCCGGTGCTCTCGCCACCGGGGGCGGCGGCGGAGCCACCGCCG

GCGCCGCCATCTGCCACGAGCAGCAGCCGTTGAAGAGCTACTCCGCCAGCACCATTGATC

CTTGTTCACTGCAATACAACAGGGAAATGGCGAGCAAGCAGCAACACGAGTGCGAGCAAG

TGCAGGACTCGGACACCCTCTCGATGCTGACCTCCATGAGCGCGAGGAACACCAACACGG

GCAGCATGTTCCCGTTCTCAAAGGAACATCATAACCACAATCCTTTCGAGGTGAGGAGCT

CGAGGCCGGACTACGGGCTGGTTTCCTCCGACTCGCTGATGAGCTCCCCCCACAGCTCCC

TGGAGAACGTCAACCTGCTCACCTCGCAGCGAGCGCTCTCGAGCGAGCAGCAGAGCTCGC

TCTCCCTGCAGCACTTCGCGGACTGGCCGAGGACGCCGTCGCAGCAGGGGCAGGGAGGGG

GAGGCCTCTCATGGCCGGACGCCGAGAACATGCAGCTGGCTCATCAGCGGACCCAGCAGC

TCTCGGTGTCCGCCGCTCCGATGGCGTCCTCCGACCTGTCGTCGGCCTCCACGTCCCCCA

TCCACGAGAAGCTCATGCTGTCGCCCCTCAAGCTGAGCCGCGAGTACAGCCCCATCGGCC

TCAGCGTCGCGGCCACGGCGGCAGCGGCGGCGAAGGACGAGGGGGAGGCGAACTGGATGC

CCATGTTCCGCGACTCGTCCATGGGCGGGCCACTGGGGGAGGCTCTGAACAAGAACAATG

GCGGCAACATGGAGGCCAAGAACTACCTGTCGGCGTCGCTGAACCTCATGACGGACGCCT

GGGACTCGAGCCCGCTGGAGTCGTCGCCGGTGGGGGTCCTGCAGAGGACCGCCTTCGGGT

CGGTGTCCAGCAGCACCGGCAGCAGCCCCAGGCAGGAGTACCACGGCGTGTATGATGGTA

ATCCGCGGGATGATCTCGGCTCCATCGTCGTGAATCACCCCAGCATCCGCCTCATGTGAG

CTTCTTCTTCCGAGCTCTGACCATGGAGAGCTGCAACGAGAAGGCTGGCCGGTTGGCAAC

GAACGAACGAGCTTGGGCAGGTCTTCTTCTTCACGTGCCTTGTTACATATCAACATCATC

ATATCCAGAAGCATTTTACGTTGTAGCTTGTCATTTGAGACTAATCTGTTGTTGTCTTCT

CTGCTTCCGGATGTTGAGTTCTTTCTTTTTATCTTTTCTCTGTAACCTTAGACATCGCTG

GGTATTGACGTTGTGTGTGTGGATGGATTTGGTGGTGCTACGCTGTTGCATAATGTGATA

ATGTCCAGTTAATGCTGCGATGCCATAATAGTTGTTGAGTAATGCAAGCTGGCATA

>TdGRF8

TTCCTCGACGAGCAGAAGATGCTCAGCTTCTCCAAGGCCGCCGCCGCTCACCAGCCGCCC

TCAGGTCTTTCTTGGATCCGCTCCTCCCTCTTCCACACATTTTTTCTGGCATCTGTGATG

TACTCCGCTCGATCTGCTCTCTGCAAGCCATGATAGATGGGCAGTCCTCTGGCGTCCTAC

TGTTCCCATGTTCAAAGATAAAAGGAAAAGAGTCAAAAGCAGCACTCCATGAGCCCTCTC

TCTCACCTCTCACTCAGCAGCGCCACCAGCTCGATTCATGGCCATGGGGATTCGCTCGAA

CCTACCCATTCACTGGGGGCCCTCCTGCTCAGCCAAGGCTGCTCCATTCCATTGCTTCTT

TTTACTGCTTTATTTATCTACACTTTTGTCTTCACTCGGCGCCTGCAATAACAAGAAACA

AATAAATAAGCATATCCATGGATATTTGCAAGATAAGGCTGTTGCTTCACTGAGCATTTT

TGCTCATGCTGAGTGGGGTTTCCTCTTCTTCTTTTTGCATCTAGGTATGGATTTTGGGAG

GTCCAATGAGCAGAGGCTGTTGCTGGCCAGGAGCAAGATGCCCTTCACCCCTTCACAGTG

GATGGAGCTGGAGCACCAGGCCCTCATTTACAAGTATCTCAATGCAAAGGCCCCCATACC

TTCCGGCCTGCTCATCTCCATCAGCAAGAGCTTCAGACCCTCCTCCGATAGAATGCCCTG

GAGGCCTGTCTATCAAGGGTTCACCAATGCAGATTCTGACCCGGAACCTGGAAGATGCCG

TCGAACAGACGGCAAGAAATGGCGGTGCTCAAAGGAGGCGATGGCCGAGCACAAGTACTG

TGAGCGGCACATCAATAGGAACCGCCATCGTTCAAGAAAGCCTGTGGAAAACCAAACAAG

GAAGAACGCCAAAGAGACACCTGCTGCTGGCTCGATATCGGCCGCTGTCTCACAGGGTGG

CTGTAAGAAAGCAAAAGCTGGTGATGAACTGAAGCCAGGGAGCGTCAGTTATTGGACAGA

TAATTTAAACAGGTTTGTATACATTGAATCTTAGTTTCTTATATGGATCAGAACTATTTC

ACAGCTATGGAGTGACTGAAAAAATGCAACTGCTAAGCTAGGGAGGCAAAGAAACAAGCA

AAAACATTTAAATTTTATTGCAAGGTCATAATTTCGATTTAACACATGTACTCGTTTGCA

GGGCAATGGTGAGCAAAGCCAGGGGAAACAACCCTGAAGAAGGCAACAGTGCTCCACTCC

TGAATTCTACTAATCAACAACACACATTGTCCTTGTTCTCTCAACTGAAGCAACAGAGCA

AACCAGATAAGTTCAGCCCGGCAGTCGATAGTGAATCGATCTCCTCAAATACTGTATTGA

AGCCTTGGGAAAGAAGCAACCAGCAGAGCAGTAAGGACGTTTCTTCCACGACGCTCCATG

ATCGCGGGTGCCTTCAATCAGTCCTTCAAGATTTCAGCATGCATAAGAATGACAAGATCG

AGTCTCAGAAAAACAATGCTTCAGTGCCATCTACTTTCTATTCATCTACAGAAGGTCGAC

ACATCAGCTGCCTTGCATCTAACATGATGCAAGTGCAGGAGGATTGCATCTCAAGCTCTT

GGGAGATACCTCAAGGTGGGCCTTTAGGTGAAATCCTAACAAACTCCAAGAATACTGATG

ACTTGACCAATAAGTGTGAATCAAGATCATATGGTTGGTTACTGAGTCTTGATGAACATG

AAATGTGAGTTCGCAGTAAATGGAGAGCTAGACATAGCAAAATTCACCATGGAAGGGAAC

AATGCTGATGGAAGCAGGATGAAGTAGCTTAGATGTGTGACCCATCCATGCTTGTTCTTC

TCTCCATATTTTCAATCTTCTGTTTTGTAATATCCAGTAGTTGCAACTTTTAGTGTTTAG

GAATTTGGCTGTATCCTAAAATATTATGGCTTGCCTTGTATCAAGCTTATTAGCTACATC

TTTTAACAGGTAAACTATAAGGATCCATTTCTTCATATCCATCCAGTAGTTCCAACTTCC

AAGGAACTTTTCTGAAATTATTTGAGCCCATAACACCCATGGTTCTGTTTGTCAATGTCC

AAGACCATAACTGTCAACTCTTCATAAGGATGGCATATATTGTAATAATCATATAACAAG

CAGATGAATATCAATTCAAATCGTCCTGACAAACATATAAGGCCATTTGGTTATCAAGCC

TTGGACAACTTACTGCAGGTTAAATGAGCCCAGTAATCATAGTACAATTATACAGTCAAT

TTCTGGAAACTTTTATCATACTTTCTGAGGACAGTTTATCATCTCTAGTATGATTCAAAA

GAATGAACCCATGTCTGGTACGGTGATGGCCCTTAAATTTGATATACTCCTGTATTGATT

ATGTCCAGGACAGGATTGATACAGTGAGGAGTATTACAGCAGAGATACGTATCAATAGGA

CATCCGGCAGATAAGAACAGTATCCAAATTTAAACAAGTGCACTGCACAAACAAACAAGT

GCGCCGGCTCAGACACGCACAAATAAAGTCATCATATTTGGTCCTTAACTGAAATTAAAT

TATGAACAAATAATCTAGTTGTCATCACAGCCAGGCAAACCAGTTCCTCTATTACACAAT

TTTGTCACCTAAAACACAAGAACAAAAATCATGACAAAAACCAACGTTAC

>TdGRF9

NNNNNNNNNNNNNNNNNNNNNNNNNNNNNNNNNNNNNNNNNNNNNNNNNNNNNNNNNNNN

NNNNNNNNNNNNNNNNNNNNNNNNNNNNNNNNNCCCTCCCTCCCCTCCATTGATGGCCGC

CACCAAGAACCCCCCAACCCCCACCTGACCGCCGTGCCCCGCCGCGCGTTCTCGCCCGCC

TTGCCTTGCGCCGAGAGGAGGAGCCGGGCCTACTACTATCTTGCTCCAAGGCGGAGGCTT

TACGCCGCTGGAGATGCTGAGCTCGTCGGCGGCGATGGGGATGGGGCTGGGGGGCTACGG

CCAGCAGCAGCAGCAGCAGATGCAGATGCAGATGCAGCGGGGCGCGGGGCCGGTGTTCAC

GCCGGCGCAGTGGGCCGAGCTGGAGCAGCAGGCGCTGATTTACAAGTACCTCATGGCAGG

CGTGCCCGTGCCGCCCGATCTCCTGCTCCCCATCCGCCCCCACCACCCCGCCGCCGGCGC

CGCCGGAACCACCTTCTCCTTCGCCAGCCCCGCCGCCTCGCCCTTCTACCACCACCACCA

TCCCTCCATGAGTTACTACGCCTACTATGGCAAGAAGCTCGACCCGGAGCCGTGGCGGTG

CCGGCGCACCGACGGCAAGAAGTGGCGGTGCTCCAAGGAGGCGCACCCCGACTCCAAGTA

CTGCGAGCGCCACATGCACCGTGGCCGCAACCGTTCAAGAAAGCCTGTGGAATCCAAGTC

TGCTTCCCCTGCGCACCAGTCGCAGCAGCCCCCGCTGTCCGCCGTCACGTCCGCGGCCCG

CGACGCCGAGCCGCTCCCCTCCCTCCCGGCTGGGGCTAAAACCCATGGCCTGTCCCTCGG

CGGGGCTGGCTCGTCGCAGATGCACGTCGACGCCTCATCATACGGCGGCAAATACTCCCT

TGGAGCTAAATCTGATGTGGGTGAACTGAGCTTCTTCTCTGGAGCATCAGGAAACAACAA

CAGGGGCTTCACCATCGATTCCCCAACGGACAGCTCGTGGCACTCGATGGGGTCCAGCCT

GCCCCCGTACCAACTGTCGAAACCTAGAGATTCCGGCCTCATGCAAGGCGGCTTCTCGTA

TTCCCACTATGAGCCGTCGCAGGAGCTTGGGCAGGTAACCATCGCCTCGCTGTCCCACTC

CCAGGAGCAGGACCGCCGCTCTTTCGGTGGTGGAGGTGGAGGAGGTGGAGGTGGAGGTGG

AGGGCTCATGGGAAATGTCAAGCAGGAGAACCAGCCGCTGAGGCCCTTCTTCGACGAGTG

GCCGGGGAGGCGGGACTCGTGGTCGGAGATGGACGACGAGCGCTCCAACGGCACCTCCTT

CTCGACGACCCAGCTCTCGATCTCCATCCCGATGCCTCGATGCGATTGA

>TdGRF10

TACCCCTCCCCCTTCCTCGACGAGCAGAAGATGCTCAGCTTCTCCAAGGCCGCCGCTCCT

CCATCGTCAGGTATGGATTTCGGGAGGTCAAATGAGCAGAGGCTGCTGCTGGCCAGGAGC

AAGATGCCCTTCACACCTTCGCAGTGGATGGAGCTGGAGCACCAGGCCCTCATATACAAG

TACCTCAACGCGAAGGCCCCCATACCCTCCGGCCTGCTCATCTCCATCAGCAAGAGCTTC

AGACCCTCCTCCGATAGAATGCCCTGGAGGCCTGTCTACCAAGGGTTCACCAATGCAGAT

TCTGACCCGGAGCCTGGAAGATGCCGTCGAACAGACGGCAAGAAATGGCGGTGCTCAAAG

GAGGCGATGGCCGAGCACAAGTATTGTGAGCGGCACATCAATAGGAACCGCCATCGTTCA

AGAAAGCCTGTGGAAAACCAAACAAGGAAGAACGCCAAGGAGACACCTGATGCTGGCTCG

TTATCGGCCGCTGTCTCACATGGTGGCTGTAATAAGAAAGCAAAAGCTGGTGATGAACTG

AAGCCAGGGAGCGTCAGCTATTGGACAGATAATTTAAACAGGGCAATGGTGAGCAAAGCC

AGGGGAAGCAACCCTGAAGATGGCAACAGTGCTCCACTCCTGAATTCTACTAATCAACAA

CACACATTGTCCTTGTTCTCTCAACTGAAGCAACAGAGCAAACCAGATAAGTTCAGCCCG

GCAGTCGATAGTGAATCGATCTCCTCAAATACAGTGTTGAAGCCTTGGGAAAGAAGCAAC

CAGCAGAGCAGCAAGGACGTTTCTTCCACGACGCTCCATGATCGCGGGTGCCTTCAATCA

GTCCTTCAAGATTTCAGCATGCATAAGAATGAGTCTCAGAAAATCAATGCTTCAGTGCCA

TCAACTTTCTATTCATCTACAGAAGGTCGACACATCAGCTGCCTTGCATCTAACATGATG

CAAGTGCAGGAGGATTGCATCTCAAGCTCTTGGGAGATACCTCAAGGTGGGCCTTTAGGT

GAAATCCTAACAAACTCCAAGAACACTGATGACTTGACCAATAAGTGTGAATCAAGATCA

TATGGTTGGTTACTGAGTCTTGATGAACATGAAATGTGAGCTCGGGAGAGCTAGACATGG

CAAAATTCACCATGGAAGGGAACAATGCTGATGGAAGCAGGATGAAGTAGCTTAGATGTG

TGACCCATCCATGCTTGTTCTTCTCTCCATATTTTCAATCTTCTGTTTTGTAATATCCAG

TAGTTGCAACTTTTAGTGTTTAGGAATCTGGCTCTATCCTAAAATATTATGGCTTGCCTT

GTATCAAGCTTATTAGCTACATCTTTTAACAGGTAAACTATAAGGATTCATTTCTTCATA

TCCATCCAGTAGTTTCATGGAACTTTTCTGAAATTATTTGAGCCCATAACACCAATGGTT

CTGTTTGTCAATGTCCAAGACCATAACTGGCAACTCTTCAGAAGGAATAACATATATTGG

AATAATCATATAACAAGAAGATGAATATCAATTCAAATCGTCCTGACAAACATCGAGGCA

TCGAGGGCCATCTGCAGGTTATATGAGCCCAGTAATCATAGTACAATTATACAGTCAATT

TTCTGACAGTTTATCATCTCTGGTATGGTTCAAAAGAATGAACCCATGTCTGGTATGGTG

ATGGCCCTTAAATTTGATATACTCCTGTATTGATTATGTTCAGGACACGATTGATACAGT

GAGTATTACAACATAGATGCTTATCAATAGGACATCCGGCAGATAAGAACAGTATCCAGC

TAGCTACCTATGCAGGATCATACAGCCTCTCGTGGGCCGAAGTTATTCGATTTTGCCAGT

TTCTCATTATGAAGCAAGTTCCTGAAAAAGCCTTTTTCTTCATTGTGCCCGTAGGGAAAT

ATCAATCATAAGAACAATTTTGATCTCAGTCACACATGAAAATGCAAAGTGAATATGATG

AAGTGATCATATCCGCCTAATCTAGTTTGCAAATGCATGAGAATCAATAAATATACAGGG

TTGAAATGAGTGCCTTTAATAGTCAGTTAATCAGAAACAAAACCTGGGCAAACCAAACTG

AAATAGTACAAAAGGTTTAGCACACAGGAGCACACCAAGAAGAAGCAACATTGGCTCAGA

TACGCACAAAGCTCGTCAAATCAAATAAAGTATCAGCACCGAGATACAGAATATCTCTGC

TAGACAAGAACACTAGAAGCAAATAAAAAGGACATCACAACTGAAGATGCCAAACTGAGT

TTACACACCGAAAAATACAAAAACCGAGCTAACACACTACAAAGCACAAAGGCATATTTG

CATATGTCGAAGGCAGGCCTAACCCGAAGTCCAACCATATTCATGGAACAAGGGCTATCT

TGCGTTGCATCAGTATGGGAGTAACTGAAGCCAATAGCTATCAAGAAGCAATGGCTTGCC

AAAATTGTGGAACTCCCATGAAGAATCATTGGTGATGTTACATAAACCACAATAGATACT

GGAGAATGCAAAATCGATATGCTAGTTCGTCTAATACACAGTTGTCACCTAAAATACAAG

AACAAATAATTGAGACCAAAAAAAC

>TdGRF11

CTCAGATTTCCTCCCGCCTCTTGCTTGCTTGCCCCTGTCGAGAAAGTACTACACATTTAT

TTGTTTTGGATTTGCCTTCTTGTCTTGCACTGCCTGCCTTGTTGCTCATCTGCTTGTTGT

ATTGTTATCATGGGTGCCAGGGTTGAGCTCGGTGAGCTTGAGCGCCAGCATCCAGAACGC

CATGGCCAGGGTGAGGGGGCCCTTCACGCCGTCGCAGTGGATGGAGCTGGAGCACCAGGC

CCTGATCTACAAGTACCTGGCGGCCAACATCGCCGTGCCTCACAACCTGCTCGTCCCCAT

CCGCCGGAGCGTCACCTCGCTCTACCCGTCCGCCTACTTTGGCTCCTCCACATTGGGGTG

GGGGCCTTTCCAGCTGGGCTACTCCGGGAGCGCGGACCTGGAGCCCGGGCGGTGCCGCCG

GACGGACGGCAAGAAGTGGCGGTGCTCCAGGGACGCCGTCGCCGACCAGAAGTACTGCGA

GCGGCATATGAACCGGGGACGCCATCGTTCAAGAAAGCATGTGGAAGGCCAGCCTGGCCA

TGCCGCGAAAGCGATGCCTGCGACGGTGGCGGCGGCTGCTGCCCAGCCCGGTGCTCTCGC

CACCGGGGGCGGCGGCGGAGCTACCGCCGGCGCCGCCGCCATCTGCCACGAGCAGCAGCC

GTTGAAGAACTACGCCGCGAACACCATTGATCCTTGTTCACTGCAATATAACAGGGAAAT

GGTGAGCAAGCAGCAGCAACACGAGTGCGAGCAAGTGCAGGACTCCGACACCCTCTCGAT

GCTGACCTCCATGAGCGCGAGGAACACCAACACGGGCAGCATGTTCCCGTTCTCAAAGGA

GCATCACAATCACAATCCTTTCGAGGTGACGAGCTCAAGGCCGGACTACGGGCTGGTTTC

ATCCGACTCGCTGATGAGCTCCCCTCACAGCTCCCTGGAGAACGTCAACCTGCTCACCTC

GCACTCGCAGCGAGCGCTCTCCAACGAGCAGCAGAGCTCGCTCTCCCTGCAGCACTTCGC

GGACTGGCCGAGGACGCCCTCGCAGCAGGGGCAGGGAGGAGGAGGTCTCTCATGGCCGGA

CGCCGAGGACATGCAAGCACATCAGAGGACCCAGCTCTCGGTGTCCGCCGCTCCAATGGC

GTCCCCCGACCTGTCGTCGGCCTCCACGTCCCCGATCCACGAGAAGCTCATGCTGTCGCC

CCTCAAGCTGAGCCGCGAGTACAGCCCCATCGGCCTCAGCATCGCGGCGACGGCGGCGGC

GGCGAAGGACGAGGGGGAGGCGAACTGGATGCCCATGTTCCGCGACTCGTCCATGGGCGG

GCCGCTGGGGGAGGCCCTGAACAAGAACAATGGCGGCAACATGGAGGCCAAGAACTACCT

GTCGGCGTCGCTGAACCTCATGACGGACGCCTGGGACTCGAGCCCGCTGGAGTCGTCCCC

GGTGGGGGTCCTGCAGAGGACCGCCTTCGGGTCGGTGTCGAGCAGCACCGGCAGCAGCCC

CAGGCAGGAGTACCACGGCGTGTATGATGGTAACCCGCGGGATGATCTCGGCTCCATCGT

CGTGAATCACCCCAGCATCCGCCTCACGTGAGCTGCTTCTTCCGAGTTCTGACCATGGCA

GCTGCAACGAGAAGGCCCAGGCCGGTTGTCACTCCTGTGGCAACGAACGAACGAACTTGG

GCAAGTCTTCTTCTTCACGGACCTTGTTACATATCGACATCATCGTATCCAGAAGCATTT

TACGTTGTAGTTTGCCATTTGAGACTAATCTGTTGTTGTCTTCTCTGCTTCGGGATGTTG

AGTTCTTTTTGTTACCTTTTTCTCTGTAACCTTTAGACTTCGCTGGGTATTGACGTTTTG

TGTGTGTGTGTGTGTGTGGATGGATTTGGT

>TdGRF12

ATGGCCGACGAGAAAGAAGCCGACTCGCTGCAGCCGCCGTCCAAGCAGCCCCGCCTCTCC

TCCGCCGACTCGAACGCCGGGGCGGTGACGATGGCGGTCTTGTCGCCGCTGGGTCTTGGC

CTCGGCCTGGGGCTCGGTGGCGATAGCCGTGGCGAGCAGCAGGCCTTCGAAGCACGGGCG

GCGGCGAAGTCGGCGCTGACGTTCATGCAGCAGCAGGAGCTGGAGCACCAGGTGCTCATC

TACCGCTACTTCGCGGCGGGGGCGCCCGTGCCGGTGCACCTCGTGCTGCCCATCTGGAAG

AGCGTCGCCGCCTCCTCCTTCGGCCCGCACCGCTTCCCCTCCCTGATTGGGCTGGGGAGC

CTGTGCTTCGACTACCGGAGCAGCATGGAGCCGGAGCCCGGGCGGTGCCGCCGCACGGAC

GGCAAGAAGTGGCGGTGCTCCCGCGACGTGGTGCAGGGGCACAAGTACTGCGAGCGGCAC

GTCCACCGGGGCCGCGGCCGTTCAAGAAAGCCTGTGGAAGGAGCCCCCTCAGCCCGGGCG

CACAGCGACAGCACCGCCACCGCCCCGCCCTGCGCCATCGGCTTCTCCCCCGCCGGCATC

CTCCACGCCACCCACAGCGCCGCCGCGCGCGCCACCTGA

>TdGRF13

CGTATGCCTCTCTTTCCCCGGCAGGCGACCGCCGCTCCTCCCCGGCCGCCACCGCCACCG

CCTCCCTCCTCCCCTTCTGCCGTTCCTCCCCCTTCTCCGCGTAAGCATCCACCGTCCACC

GCTCGCTTCTTGGCTCCTCCCTCATCTTCTTTCTTGTGTTGTGTGACGTGGTTCGGTGCG

TGTTCTTGTGCGCAGCGGCGGCAATGGCGGCATGGGGGAGGAGGCGCGGATGGACGGGAG

GTGGATGGCGAGGCCGGTGCCCTTCACGGCGGCGCAGTACGAGGAGCTGGAGCACCAGGC

GCTCATATACAAGTACCTGGTGGCCGGCGTGCCCGTCCCGCCGGATCTCGTGCTCCCCAT

CCGCCGCGGCATCGAGTCCCTCGCCGCCCGCTTCTACCACAACCCCCTCGCCATCGGGTA

CGGATCGTACCTGGGCAAGAAGGTGGATCCGGAGCCGGGCCGGTGCCGGCGCACGGACGG

CAAGAAGTGGCGGTGCGCCAAGGAGGCCGCCTCCGACTCCAAGTACTGCGAGCGCCACAT

GCACCGCGGCCGCAACCGTTCAAGAAAGCCTGTGGAAACGCAGCTCGTGCCCCACTCCCA

GCCGCCGGCCGCCTCCGCCGTGCCGCCCCTCGCCACCGGCTTCCACAGCCACTCCCTCTA

CCCCGCCATCGGCGGCGGCACCAACGGTGGTGGAGGCGGGGGGAACAACGGCATGTCCAT

GCCCGGCACGTTCTCCTCCGCGCTGGGGCCGCCTCAGCAGCACATGGGCAACAATGCCGC

CTCTCCCTACGCGGCTCTCGGCGGCGCCGGAACATGCAAAGATTTCAGGTATACCGCATA

TGGAATAAGATCTTTGGCAGACGAGCAGAGTCAGCTCATGACAGAAGCCATGAACACCTC

CGTGGAGAACCCATGGCGCCTGCCGCCATCTTCTCAAACGACTACATTCCCGCTCTCAAG

CTACTCTCCTCAGCTTGGAGCAACGAGTGACCTGGGTCAGAACAACAGCAGCAACAACAA

CAGCGGCGTCAAGGCCGAGCGACAGCAGCAGCAGCAGCCGCTCTCCTTCCCGGGGTGCGG

CGACTTCGGCGGCGGCGACTCCGCGAAGCAGGAGAACCAGACGCTGCGGCCGTTCTTCGA

CGAGTGGCCGAAGACGAGGGACTCGTGGTCGGACCTGACCGACGACAACTCGAACGTCGC

CTCCTTCTCGGCCACCCAGCTGTCGATCTCGATACCCATGACGTCCTCCGACTTCTCCGC

CGCCAGCTCCCAGTCGCCCAACGGCATGTGA

>TdGRF14

ATGGCCGACGAGAAAGAAGCCGACTCGCTGCAGCCGCCGTCCAAGCAGCCCCGCCTCTCC

TCCGCCGACTCGAACGCCGGGGCGGTGACGACGGCGGTCTCGTCGCCGCTGGGTCTTGGC

CTCGGCCTGGGGCTCGGCGGCGATAGCCGTGGCGAGCAGCAGGCCTTCGAAGCACGGGCA

GCCGCGGCGGCGGCGAAATCGGCGCTGACGTTCATGCAGCAGCAGGAGCTGGAGCACCAG

GTGCTCATCTACCGCTACTTCGCGGCGGGTGCGCCCGTGCCGGTGCACCTCGTGCTCCCC

ATCTGGAAGAGCGTCTCCGCCTCCTCCTTCGGCCCGCACCGCTTCCCCTCCCTGATTGGG

CTGGGGAGCCTGTGCTTCGACTACCGGAGCAGCATGGAGCCGGAGCCCGGGCGGTGCCGC

CGCACGGACGGCAAGAAGTGGCGGTGCTCCCGCGACGTGGTGCAGGGGCACAAGTACTGC

GAGCGGCACGTCCACCGGGGCCGCGGCCGTTCAAGAAAGCCTGTGGAAGGAGCCTCCGCA

GCCCCGGCGCACAGCGGCAGCCCCACCACAGCCCCGCCCCGCGCCATCGGCTTCTCCCCC

GCCGGCATCCTCCACGCCACCCACAGCGCCACCTGAGGCCGACGGCCTCGTCCCATTCCC

ACCGCGTCCGTCCGTCGGTCATGGCGCGTGGGCGTGGGGGCAAAATCGTGC

>TdGRF15

AGACAGCGGCCGAGAGAGAAAGATGGCGATGCCGTATGCCTCTCTTTCCCCGGCAGGCGA

CCGCCGCTCCTCCCCGGCCGCCACCGCCTCCCTCCTCCCCTTCTGCCGCTCCTCCCCGTT

CTCCGCCAGCGGCAATGGCGGCATGGGGGAGGAGGCGCGGATGGCCGGTAGGTGGATGGC

GAGGCCGGCGCCCTTCACGGCGGCGCAGTACGAGGAGCTGGAGCACCAGGCGCTGATATA

CAAGTACCTGGTGGCCGGCGTGCCCGTCCCGCCGGATCTCGTGCTCCCCATCCGCCGCGG

CATCGAGACCCTCGCCGCCCGCTTCTACCACAACCCCCTCGCCATCGGGTATGGATCGTA

CCTGGGCAAGAAGGTGGATCCGGAGCCCGGCCGGTGCCGGCGCACGGACGGCAAGAAGTG

GCGGTGCGCCAAGGAGGCCGCCTCCGACTCCAAGTATTGCGAGCGCCACATGCACCGCGG

CCGCAACCGTTCAAGAAAGCCTGTGGAAACGCAGCTCGTCTCGCACTCCCAGCCGCCGGC

CGCCTCCGTCGTGCCGCCCCTCGCCACCGGCTTCCACAACCACTCCCTCTACCCCGCCAT

CGGCGGCACCAACGGTGGTGGAGGCGGGGGGAACAACGGCATGCCCAACACGTTCTCCTC

CGCGCTGGGGCCTCCTCAGCAGCACATGGGCAACAATGCCTCCTCACCCTACGCGGCTCT

CGGTGGCGCCGGAACATGCAAAGATTTCAGGTATACCGCATATGGAATAAGATCTTTGGC

AGACGAGCACAGTCAGCTCATGACAGAAGCCATGAATACCTCCGTGGAGAACCCATGGCG

CCTGCCGCCATCGTCTCAAACGACCACATTCCCGCTCTCAAGCTACGCTCCTCAGCTTGG

AGCAACTAGTGACCTGGGTCAGAACAACAACAGCAGCAGCAGCAACAGTGCCGTCAAGTC

CGAACGGCAGCAGCAGCAGCAGCCCCTCTCCTTCCCGGGGTGCGGCGACTTCGGCGGCGG

CGGCGCCATGGACTCCGCGAAGCAGGAGAACCAGACGCTGCGGCCGTTCTTCGACGAGTG

GCCCAAGACGAGGGACTCGTGGTCGGACCTGACCGACGACAACTCCAGCCTCGCCTCCTT

CTCGGCCACCCAGCTGTCGATCTCGATACCCATGACGTCCTCCGACTTCTCGGCCGCCAG

CTCCCAGTCGCCCAACGTATTTGTCGTGTCGGATTTGTATCAACAG

>TdGRF16

CCGCCACCAAGAACCCCCCAACCCCCACCTGACCGCCGCGCGTTCTCGCCCGCCTTGCGC

CGAGAGGAGGAGCCGGGCCTGTTGTTGTTATCTTGCTCCAAGGCGGAGGCTTTAAGCTGG

TTTCTTGCCGCAAGATGCTGAGCTCGTCGGCGGCGATGGGGATGGGGCTGGGCGGGTACG

GCCAGCAGCAGCAGCAGCAGCAGATGCAGATGCAGATGCAGCGGGGGGCGGGGCCGGTGT

TCACGCCGGCGCAGTGGGCCGAGCTGGAGCAGCAGGCGCTGATTTACAAGTACCTCATGG

CGGGCGTGCCCGTGCCGCCCGATCTCCTGCTCCCCATCCGCCCCCACCCCGCCGGCGCCG

GAGCCACCTTCTCCTTCGCCAACCCCGCCGCCTCGCCCTTCTACCACCACCACCACCCCT

CCATGAGTTACTACGCCTACTATGGCAAGAAGCTCGACCCGGAGCCGTGGCGGTGCCGCC

GCACCGACGGCAAGAAGTGGAGGTGCTCCAAGGAGGCGCACCCCGACTCCAAGTACTGCG

AGCGCCACATGCACCGTGGCCGCAACCGTTCAAGAAAGCCTGTGGAATCCAAGTCTGCTT

CCCCTGCGCACCAGTCGCAGCAGCCCCCGTTGTCCGCCGTCACGTCCGCCACCCGCGACG

CCGAGCCTCTCCCCTCCCTCCCGGCGGGGGCTAAGACCCATGGCCTGTCCCTCGGCGGGG

CTGGCTCGTCGCAGATGCACGTCGACGCCTCGTCATACGGCAACAAATACTCCCTTGGAG

CTAAATCTGACGTGGGTGAACTGAGCTTCTTCTCTGGAGCATCAGGAAACAACAACAGGG

GCTTCACCATCGATTCCCCAACGGACAGCTCGTGGCACTCAATGGGATCCAGCCTGCCCC

CGTACCAACTGTCGAAACCTAGAGATTCCGGCCTCATGCAAGGCGGCTTCTCGTATTCCC

ACTTTGAGCCGTCGCAGGAGCTTGGGCAGGAGAACCAGCCGCTGAGGCCCTTCTTCGACG

AGTGGCCGGGGAGGCGGGACTCGTGGTCGGAGATGGACGACGAGCGCTCCAACGGCACCT

CCTTCTCGACGACCCAGCTCTCGATCTCCATCCCAATGCCTCGATGTGATTGACGGGTGC

GAGCGGCAGGTGCAGGGCGTGCACGTACCTGCGCTGTGGCCTCTCCTGGTTCTTCGTGTC

CATTGTATCCGTGCTATGAACTCGGTTTAAGAGCAAAACACTTGCGCGTAGTTGTTGTTG

GCGGAGATAAGAGGAACCTGTAGCTGTTTTCAAGATTCCACGTTGCGTTGTACGCTCACT

CTCGGACAAGTTTGTGTGTGTTGTTGTTGCGGTAGATCATCTTGTGGAATGTATAACTGC

CTATTTGTCGGTGTTGAATGAATCCGATGTTGCTTACTTGGACAAGATCTCCTGGGACGC

G

>TdGRF17

ATGATGCTGGGAGGGCACGGCGGCGGCGGCGGGAGGTGCCTGTTCACGGCGTCGCAGTGG

CGGGAGCTGGAGCACCAGGCGCTCATCTACAAGTACATGGCCGCCGGCTCGCAGGTGCCC

CACGAGCTGGTCCTCCCGCTCCGCCACCGCGACGCCGCCGCCTTCGCCGCCATCGACACC

GCCCCCTCCCTCGCCTGCTACCCTCCTCCGCAGCCCTCCCTGGGGTGGGGGCTCTACGGG

GCGGGGGCGCAGTACGCGCGGAAGCCGGAGGACCCGGAGCCCGGGCGGTGCCGGCGGACG

GACGGCAAGAAGTGGCGCTGCTCCAGGGAGGCGTACGGGGAGTCCAAGTACTGCGACAGG

CACATGCACCGCGGCAAGAACCGTTCAAGAAAGCCTGTGGAACCGATGAGCTCCTCCTCC

TCCGTCTCCTCCCCGGCCGCCTCCTACCGCCAGACCACCCTCTCCATGTCGCCCCCCACG

CCGGCCGACACGCCCAGCTACGGCCACGGCCACCTCCGCGCAGCTGCTTCTCAGAGCCAG

ATAAACCCTCTCCAGCTCCACCTCGACACCCCGTCGCCCCCGCCGTCCTACCACAGGTAC

GCGCCGGCGCAGCAGTACGGGGGCTCCTTCCCGAGCAGGCAGCAGGTGCAGGAGGAGGCC

GAGGCGGAGGCGAGGCGGCGGCAGCACTTCCTGGCTCTCGGCGCCGACCTGAGCCTGGAC

AAGCCGGACGCCACCACCGCGGCGTCCTCGACAACCGAGGAGAAGCCGCTGCGGCGCTTC

TTCGACGAGTGGCCGCGCGACGGGAACGCCGTCGAGGTTCGGCCCTGGAATATGGGCCAC

CGGGACGAGACGCTGCTCTCCATGTCCATCCCCACGACGACGGCCTCGCACCCCGACCTC

GCCGCCTACCGCCACCACAACGCGCATTCTTCTGAT

>TdGRF18

ATGATGCTGGGAGGGCACGGCGGCGGCGGCGGCGGCGGGAGGTGCCTGTTCACGGCGTCG

CAGTGGCGGGAGCTGGAGCACCAGGCGCTCATCTACAAGTACATGGCCGCCGGCTCGCAG

GTGCCCCACGAGCTGGTCCTCCCGCTCCGCCACCGCGACGCAGCCTTCGCCGCCATCGAC

ACCGCCCCCTCCCTCGCCTGCTACCCTCCTCCGCAGCCATCCCTGGGGTGGGGGCTCTAC

GGGGCGGGATCGCAGTACGCGCGGAAGCCGGAGGACCCGGAGCCCGGGCGGTGCCGGCGG

ACGGACGGCAAGAAATGGCGGTGCTCCAGGGAGGCGTATGGGGAGTCCAAGTACTGCGAC

AGGCACATGCACCGCGGCAAGAACCGTTCAAGAAAGCCTGTGGAACCAATGAGCTCCGCC

TCCTCCGTCTCCTCCCCGGCCGCCTCGTACCGCCACACCGCCCTCTCCATGTCGCCCCCC

ACGCCGGCCGACACGCCCAGCTACGGCCACGGCCACGGCCACGACCACCTCCGCGCAGCT

GCTGGTCAGAGCCAGATAAACCCTCTCCAGCTCCACCTCGACACCCCGTCGCCCCCGCCG

TCCTACCACAGGTACGCGCCGGCGCAGCAGTACGGGGGCTCCTTCTTCCCGAGCAGGCAG

CAGGTGCAGGAGGAGGAGGCGAGGCGGCGGCAGCACTTCCTGGCTCTCGGCGCCGACCTG

AGCCTGGACAAGCCGGACGCCACCACCGCGGCGTCCTCGACAACCGAGGAGAAGCCGCTG

CGGCGCTTCTTCGACGAGTGGCCGCGCGACGGGAACGCCGTCGAGGGTAGGCCCTGGAAT

ATGGGCCACCGGGACGAGACGCTGCTCTCCATGTCCATCCCCACGACGACGGCCTCGCAC

CCCGACCTCGCCGCCGCCTCGCGCTACCACCACCACCACAACGATGAATAA

>TuGRF1

ATGGCGGCGCCGTCGCCGCTGGTTCTTGGGCTGGGTCTCGGCGTAGGCGGCAGCAGCAGTGACAGCGGACGCGACGACGCGGAGGCCTCTGCGGCGACGCGGCCGTCGGCGCTGACGTTCATGCAGCGGCAGGAGCTGGAGCACCAGGTGCTCATCTACCGCTACTTTGCCGCCAACGCTCCCGTGCCCGTGCACCTCGTGCTCCCCATCTGGAAGAGCGTCGCCGCTTCCTCCTCCGCCCCGCAGAGGTTTCCATCCCTGGCGGGGCTGGGGAGCATGTGCTACGACCACAGGAGCAGCATGGAGCCGGAGCCGGACCGGTGCCGGCGCACGGACGGCAAGAAGTGGCGGTGCTCGCGCGGCGTGGTGCCGGGGCACAAGTACTGCGAGCGCCACGTCCATCGCGGCCGCGGCCGTGCAAGAAAGCCTGTGGAAGCCGCGGCGGCCACATCAGCCGTCCCGATCCGCGCGATGCACGCCGCCGACGCGCAGGGCGCCACCAGTGCGCACGCGGCGCCACCGCAGCGCCTCGGCTTCTCCTCCCCCGCCGGCGTCTACCTGGCGCACGGCACCGCCCGTGCCACCTGA

>TuGRF2

ATGGCGATGCCCTTTGCCTCCCTGTCGCCGGCAGCCGACCACCACCGCTCCTCCCCCATCTTCCCCTTCTGCCGCTCCTCCCCTCTCTACTCGGCAGGGGAGGAGGCGGCGCAGCAGCAGCAGCAGCAGCAGCACGCGATGAGCGGCGCGAGGTGGGCGGCGGCGAGGCCGGCGACCTTCACGGCGGCGCAGTACGAGGAGCTGGAGCAGCAGGCGCTCATCTACAAGTACCTCGTCGCCGGCGTGCCCGTCCCGCCGGATCTCCTCCTCCCCATCCGCCGGGGCTTCGACTCCCTCGCCTCGCGCTTCTACCACCACCACGCCCTTGGGTACGGGTCCTACTTCGGGAAGAAGCTGGATCCGGAGCCGGGGCGGTGCCGGCGGACGGACGGCAAGAAGTGGCGGTGCTCCAAGGAGGCCGCCCAGGACTCCAAGTACTGCGAGCGCCACATGCACCGCGGCCGCAACCGTTCAAGAAAGCCTGTGGAAACGCAGCTCGTCTCCCACTCCCAGCAGCTGCAGCAGCACGGCCCCGCCGCCGCGTTCCACGGCCACTCGCCGTACCCGGCGATCGCCACTGGCGCCGGCGCGCCCGGCTCCTTCGCCCTGGGGTCTACTGCTCAGCTGCACATGGATAATGCTGCTGCGCCTTACGCGACCGCTGGCGCCGCCGGGAACAAAGATTTCAGGTATTCTGCCTATGGGTTTAGGACTTCGGCGATGGAGGAGCACAACCAGTTCATCAGTGCGGCCATGGACACCGCCATGGACAACTACTCATGGCGCCTGCTGCCGGCCCAGAACTCGTCCTTCTCACTCTCGAGCTACCCCATGCTGAGCACCCTGAGCGACCTGGACCAGAGCGCGATCTGCTCGCTGGCCAAGACGGAGAGGGAGCCGCTGTCCTTCTTCGGCGTGGGCGGCGGCTTCGACGACGACGAGTCGGCGGTGAAGCAGGAGAACCAGACGCTGCGGCCCTTCTTCGACGAGTGGCCCAAGGACAGGGACTCGTGGCCGGAGCTGCAGGACCATGACTCCAACCACAACAATGAGGCCTTCTCGGCCACCAAGCTGTCCATCTCCATCCCGGTGACCAGCTCCGATTTCTCCACCACCGCCGGCTCCCGCTCGCCCCACGGTATATACTCCCGGTGA

>TuGRF3

ATGGCGGCGGAAGGGGAGGACAAGAAGGATGCTAATTCCGTTGGAGGCGGCGGCGGCGGCGGCGAGAACACCCTGGAGGCGGCGGAGGAGGCGATTCTGCAGGCGGTAGGGCAAGAACCCGGTCAAGAATTGGAGGGCGAGGTGGAGGAGAGCGCAGATCGAGAGGGGAACGGCGACGACGCTGGGAAGGAAGATAGTGGGTGTAAAGATCTGGTCCTGGTAGAGGACCCTGTCCTGGTCGAGGATCCAGAGGAAGCGGTAGCAACTGCAGCACTTCAGGAAGAAATGAGAGCGCTTTTCGCGTCTGTCCCTGAAGGTGCTGGGGCATCATTTACTGCGATGCAGCTGCAGGAGCTAGAGCAGCAGTCTCGGGTATACCAGTATATGGCTGCCCGCGTGCCTGTGCCTACCCATCTCGTCTTCCCCATCTGGAAGAGTGTTACCGGTGCATCCTCTGAAGGCGCACAGAATTACCCTACATTGATGGGATTGGCAACACTCTGCTTGGACTTCGGGAAGAGCCCAGAACCAGAACCAGGAAGGTGCCGGCGAACAGATGGAAAAAAGTGGCGATGCTGGAGAAAAACAATCCCAAACGAGAAATATTGTGAACGCCATATGCATCGTGGTCGCAAGCGTCCTGTACAGGTTATTGTTGAGGATGACGAGCCTGATTCCGCATCAGGGTCAAAATCGTCATCTGGCAAAGTCACTGAAGGAGGCAAGAAGACTGACGACAAGAGTTCAAGTAGCAAGAAGCTTGCAGTGGCAGCACCAGCTGCTGTGGAGTTTACATGA

>TuGRF4

ATGGCCGACGAGAAAGAAGCCGACTCGCTGCAGCCGCCGTCCAAGCAGCCCCGCCTCTCCTCCGCCGACTCGAACGCCGGGGCGGTGACGATGGCGGTCTCGTCGCCGCTGGGTCTTGGCCTCGGCCTGGGGCTCGGTGGCGATAGCCGTAGCGAGCAGCAGGCCTTCGAAGCACGGGCGGCGGCGAAGTCGGCGCTGACGTTCATGCAGCAGCAGGAGCTGGAGCACCAGGTGCTCATCTACCGCTACTTCGCGGCGGGGGCGCCCGTGCCGGTGCACCTCGTGCTGCCCATCTGGAAGAGCGTCGCCGCCTCCTCCTTCGGCCCGCACCGCTTCCCCTCCCTGATTGGGCTGGGGAGCCTGTGCTTCGACTACCGGAGCAGCATGGAGCCGGAGCCCGGGCGGTGCCGCCGCACGGACGGCAAGAAGTGGCGGTGCTCCCGCGACGTGGTGCAGGGGCACAAGTACTGCGAGCGGCACGTCCACCGGGGCCGCGGCCGTTCAAGAAAGCCTGTGGAAGGAGCCCCCGCAGCCCCGGCGCACAGCGACAGCACCGCCACCGCCCCGCCCCGCGCCATCGGCTTCTCCCCCGCCGGCATCCTCCACGCCACCCACAGCGCCGCCGCGCGCGCCACCTGA

>TuGRF5

ATGATGATGATGGGCGGTCGCGCGGGGGCCGGCGGCGTCGGGGCAGGCGGCGGCCGGTGCCCGTTCACGGCGACGCAGTGGCAGGAGCTGGAGCACCAGGCGCTCATCTACAAGTACATGGCCTCCGGCGTGCCCATCCCCTCCGACCTCCTCCTCCCGCTCCGCCGCAGCTTCCTCCTCGACTCCGCCCTCGCCACCTCCCCCTCCCTCGCCTTCCCTCCCCAGGCCGCACTTGGGTGGGGTTGCTTTGGCATGGGGTTCGGCCGGAAGGCGGAGGACCCGGAGCCGGGGCGGTGCCGGCGGACGGACGGCAAGAAGTGGCGCTGCTCCAAGGAGGCGTACCCGGACTCCAAGTACTGCGAGAAGCACATGCACCGGGGCAAGAACCGTTCAAGAAAGCCTGTGGAAATGTCCTTGGCCACGCCCCCGCCGCCGCCTTCCTCCTCGGCCTCCTCTTCCTCCTCCAACGTCCACTCCGCCGTCAACGTCGCCACCACCACCACCTCCCCAGCGCCGTCCTACCACCGCCACGCCGCTGCCACTCACGACACGACGCCCTACCACGCCCTCTACGGCGGCCCCTACTCCTCCGCCGGCCGCCAGCAGCACGCCAGCGCCTACCACCACGCGGCGCAGGTCAGCCCGTTCCACCTGCACCTCGACACCACCCACCCGCACCCGCCGCCGTCCTACTACTCCACCATGGACCACAGCAAGGACAGCTACGCCTACGGGCACAGCGTCAAGGAGGTGCACGGCGGCGGCGAGCACGCCTTCTTCTCCTCCGACGTCAGCACCGACAGGGACCACCACCACCATCAGCACCAACACCACGCTAGCGCCGGCGGCAACGGCCAGTGGCAGTTCAAGCAGCTCGGCGGCATGGAGCCCAAGCAGCACAACCCCACGTCGCTCTTCCCCGGCTACGGCAACAACGCGGCGTACGCCATCGACCTGTCCAGCAAAGAAGAGGACGAGGAGAAGGAGAGGCGGCAGCAGCAGCAGCACTGCTTCCTGCTGGGCGCCGACCTGAGGCTCGACAAGCCGTCGTCGGGGCACGGCGACTCCGCCGACCAGAAGCCTCTCCGGCCGTTCTTCGACGAGTGGCCGCACGAGAAGACTGGCAGCAAGGGGTCGTGGATGGGGCTCGAGGGGGAGACGCAGCTCTCCATCTCCATCGCCAATGAACTCCCCATCACCACCACCTCCCGCTACCACCATGGTGAATGA

>TuGRF6

ATGATGCTGGGAGGGCACGGCGGCGGCGGCGGGAGGTGCCTGTTCACGGCGTCGCAGTGGCGGGAGCTGGAGCACCAGGCGCTCATCTACAAGTACATGGCCGCCGGCTCGCAGGTGCCCCACGAGCTGGTCCTCCCGCTCCGCCACCGCGACGCCGCCGCCTTCGCCGCCATCGACACCGCCCCCTCCCTCGCCTGCTACCCTCCTCCGCAGCCCTCCCTGGGGTGGGGGCTCTACGGGGCGGGGGCGCAGTACGCGCGGAAGCCGGAGGACCCGGAGCCCGGGCGGTGCCGGCGGACGGACGGCAAGAAGTGGCGCTGCTCCAGGGAGGCGTACGGGGAGTCCAAGTACTGCGACAGGCACATGCACCGCGGCAAGAACCGTTCAAGAAAGCCTGTGGAACCGATGAGCTCCTCCTCCTCCGTCTCCTCCCCGGCCGCCTCCTACCGCCAGACCACCCTCTCCATGTCGCCCCCCACGCCGGCCGACACGCCCAGCTACGGCCACGGCCACCTCCGCGCAGCTGCTTCTCAGAGCCAGATAAACCCTCTCCAGCTCCACCTCGACACCCCGTCGCCCCCGCCGTCCTACCACAGGTACGCGCCGGCGCAGCAGTACGGGGGCTCCTTCTTCCCGAGCAGGCAGCAGGTGCAGGAGGAGGCCGAGGCGGAGGCGAGGCGGCGGCAGCACTTCCTGGCTCTCGGCGCCGACCTGAGCCTGGACAAGCCGGACGCCACCACCGCGGCGTCCTCGACAACCGAGGAGAAGCCGCTGCGGCGCTTCTTCGACGAGTGGCCGCGCGACGGGAACGCCGTCGAGGGTCGGCCCTGGAATATGGGCCACCGGGACGAGACGCTGCTCTCCATGTCCATCCCCACGACGACGGCCTCGCACCCCGACCTCGCCGCCTACCGCCACCACAACGCAGATGAATAA
